# DNA methylation study of Huntington’s disease and motor progression in patients and in animal models

**DOI:** 10.1038/s41467-020-18255-5

**Published:** 2020-09-10

**Authors:** Ake T. Lu, Pritika Narayan, Matthew J. Grant, Peter Langfelder, Nan Wang, Seung Kwak, Hilary Wilkinson, Richard Z. Chen, Jian Chen, C. Simon Bawden, Skye R. Rudiger, Marc Ciosi, Afroditi Chatzi, Alastair Maxwell, Timothy A. Hore, Jeff Aaronson, Jim Rosinski, Alicia Preiss, Thomas F. Vogt, Giovanni Coppola, Darren Monckton, Russell G. Snell, X. William Yang, Steve Horvath

**Affiliations:** 1 Department of Human Genetics, David Geffen School of Medicine, University of California, Los Angeles, Los Angeles, CA 90095 USA; 2 Applied Translational Genetics Group, School of Biological Sciences, Centre for Brain Research, The University of Auckland, Auckland, 1010 New Zealand; 3 Center for Neurobehavioral Genetics, Jane and Terry Semel Institute for Neuroscience and Human Behavior, University of California, Los Angeles (UCLA), Los Angeles, CA 90095 USA; 4 Department of Psychiatry and Biobehavioral Sciences, David Geffen School of Medicine at UCLA, Los Angeles, CA 90095 USA; 5 CHDI Management/CHDI Foundation, Princeton, NJ 08540 USA; 6 Livestock and Farming Systems, South Australian Research and Development Institute, Roseworthy, SA 5371 Australia; 7 Institute of Molecular, Cell and Systems Biology, College of Medical, Veterinary and Life Sciences, University of Glasgow, Glasgow, G12 8QQ UK; 8 Department of Anatomy, University of Otago, Dunedin, 9016 New Zealand; 9 Department of Biostatistics, School of Public Health, University of California, Los Angeles, Los Angeles, CA 90095 USA

**Keywords:** DNA methylation, Epigenomics, Epigenetics, Huntington's disease, Huntington's disease

## Abstract

Although Huntington’s disease (HD) is a well studied Mendelian genetic disorder, less is known about its associated epigenetic changes. Here, we characterize DNA methylation levels in six different tissues from 3 species: a mouse *huntingtin* (Htt) gene knock-in model, a transgenic *HTT* sheep model, and humans. Our epigenome-wide association study (EWAS) of human blood reveals that HD mutation status is significantly (*p* < 10^−7^) associated with 33 CpG sites, including the *HTT* gene (*p* = 6.5 × 10^−26^). These *Htt/HTT* associations were replicated in the Q175 *Htt* knock-in mouse model (*p* = 6.0 × 10^−8^) and in the transgenic sheep model (*p* = 2.4 × 10^−88^). We define a measure of HD motor score progression among manifest HD cases based on multiple clinical assessments. EWAS of motor progression in manifest HD cases exhibits significant (*p* < 10^−7^) associations with methylation levels at three loci: near *PEX14* (*p* = 9.3 × 10^−9^), *GRIK4* (*p* = 3.0 × 10^−8^), and *COX4I2* (*p* = 6.5 × 10^−8^). We conclude that HD is accompanied by profound changes of DNA methylation levels in three mammalian species.

## Abstract (web summary)

Although Huntington’s disease (HD) is a well-studied genetic disorder, less is known about the epigenetic changes underlying it. Here, the authors characterize DNA methylation levels in tissues from patients, a mouse *huntingtin* (Htt) gene knock-in model, and a transgenic *HTT* sheep model, and provide evidence that HD is accompanied by DNA methylation changes in these three species.

## Introduction

Huntington’s disease (HD) is a dominantly inherited neurodegenerative disorder clinically characterized by a progressive movement disorder, cognitive dysfunction, and psychiatric impairment. HD gene-expansion carriers (HDGECs) have CAG-repeat lengths of 36 or greater on one of the alleles of the huntingtin (*HTT*) gene. HD is one of several polyglutamine disorders (including spinal cerebellar ataxias types 1, 2, 3, 6, and 7, spinal and bulbar muscular atrophy, and dentatorubral-pallidoluysian atrophy) that are caused by the expansion of unstable CAG trinucleotide repeats^1^.

Since these disorders exhibit distinct patterns of neuronal loss and clinical manifestation, the differential pathogenesis of polyglutamine disorders may be due to differences in polyglutamine protein context or the host protein function. This is despite nearly ubiquitous expression of these proteins, at least in the brain, and, in the case of *HTT*, expression throughout the body and during development. The age of onset of motor (AMO) symptoms is strongly and inversely correlated with the number of CAG trinucleotide repeats in *HTT*^2–4^. A recent study showed that *HTT* CAG-repeat length but not polyglutamine length determines the timing of HD motor onset^5^. HD patients are usually clinically diagnosed in their 40s, but the AMO ranges from younger than 10 years for individuals with high-repeat lengths to, in rare cases, over 80 years. Age plays an important role in HD; for example, the product of CAG-repeat length and chronological age (CAP score) relates to clinical progression in HD according to longitudinal studies of HDGEC cohorts^3^.

By exploiting DNA methylation (DNAm)-based biomarker of tissue age (referred to as the epigenetic clock), we have shown that HD is associated with epigenetic age acceleration (AgeAccel) and greatly disrupted changes in DNAm levels in brain tissues^6^. Recent studies have looked at methylation levels of *selected* genes in HDGECs^7^ and analyzed 13 human cortical samples^8^. DNA (de)methylation in an *HTT* gene context has also been investigated in transgenic animal models^9^. However, it is not yet known whether HD is associated with significant DNAm changes in other human tissues and in transgenic animal models.

Here we present, to our knowledge, the largest DNAm study of HD to date. We generate large methylation datasets from three species and from eight sources of DNA. We evaluate composite epigenetic biomarkers and individual CpGs in terms of their relationship with (a) HDGEC status and (b) HD progression in individuals with manifest motor symptoms. Our epigenetic clock study demonstrates that manifest HD is associated with increased epigenetic age in human blood DNA. Epigenome-wide association studies (EWAS) of HD mutation status reveal that *HTT* is the most significant locus in multiple tissues from three species. Blood DNAm levels of select genomic loci are associated with motor score progression among manifest HD patients.

## Results

Our methylation study datasets derive from three species: (I) a total of 2164 human samples across blood, lymphoblast, and fibroblast tissues collected from five datasets, (II) a total of 112 mouse tissues (cerebellum, striatum, blood, cortex, and liver) measured on two platforms (reduced representation bisulfite sequencing (RRBS) and a custom methylation array), and (III) 168 blood samples from sheep. All methylation datasets are described in Table 1 (for human) and Supplementary Table 1 (for mouse and sheep). Human blood samples were a subset of a larger collection from Enroll-HD^10^ and Registry-HD^11^ datasets; the longitudinal clinical measures from the subset of cases were used to perform the HD progression analysis.

**Table 1:** A total of 2164 methylation samples across blood, lymphoblastoid, and fibroblast tissues were used for human cross-sectional studies.

	Parameter				
	Enroll-HD data 1 (*N* = 910)	Enroll-HD data 2^a^ (*N* = 357 × 2)	Registry-HD (*N* = 376)	Registry-HD (*N* = 100)	CHDI (*N* = 64)
	DNA source				
	Buffy coat	Buffy coat	Whole blood	Lymphoblastoid	Fibroblast
No. of individuals					
Manifest HD	393	204	298	79	37
Pre-manifest	223	153	0	0	0
Control	294	0	78	21	27
Age					
HDGEC	49 [18, 76]	45 [18, 76]	53 [19, 84]	52 [19, 82]	43 [31, 58]
Control	51 [18, 76]	–	49 [20, 84]	52 [20, 82]	41 [31, 55]
CAG length					
HDGEC	43 [36, 51]	44 [37, 59]	45 [39, 67]	45 [39, 66]	44 [41, 50]
Controls	20 [16, 35]	–	21 [14, 33]	21 [17, 31]	–
Age of motor onset					
HDGEC	46 [16, 73]	44 [17, 75]	44 [8, 75]	43 [8, 73]	–
DNA methylation					
Array type	Illumina 450k	Illumina EPIC	Illumina 450k	Illumina 450k	Illumina 450k
Normalization method	Noob^57^	Noob^57^	Noob^57^	Noob^57^	Noob^57^
Data analysis	Clock, EWAS, regression	Clock, EWAS, regression	Clock, EWAS, regression	Clock, regression	Clock, regression

Columns 1–5 correspond to blood methylation data from Enroll-HD data 1, Enroll-HD data 2, and the Registry-HD, respectively. For each individual in the Enroll-HD data 2, two blood samples were collected (separated by roughly 7.9 years). Only the first sample is described in the table. The last row specifies the use of these data: epigenetic clock analysis, epigenome-wide association study (EWAS), and multivariate regression analysis. Both Enroll-HD^10^ and Registry-HD^11^ are large-scale longitudinal observational cohorts for studying the onset and progression of Huntington’s disease. The last two columns correspond to non-blood methylation data from Registry-HD and CHDI, respectively. The CHDI Foundation is a privately funded, not-for-profit biomedical research organization devoted to a single disease—Huntington’s disease.

Continuous parameters are presented in the format of mean [min, max].

HDGEC refers to HD gene-expansion carriers (combining pre-manifest and manifest individuals).

^a^HD status and age estimates reported for the first blood draw for the DNA methylation study. The dataset involves 714 methylation samples from two longitudinal measures across 357 individuals.

### Human data

We generated five human DNAm datasets using the Illumina Infinium array (*N* = 2164 samples; Table 1): (1) *N* = 910 blood samples from Enroll-HD study (data 1); (2) *N* = 714 longitudinal blood samples from 357 individuals from Enroll-HD (data 2); (3) *N* = 376 blood samples from Registry-HD; (4) *N* = 100 lymphoblastoid cell samples from Registry-HD; and (5) *N* = 64 fibroblast samples from the CHDI. The Enroll-HD study datasets (blood data 1 and 2) used in this study were a subset of a larger collection of 33,288 observations from 15,203 individuals (“Methods”; Supplementary Tables 2, 3). Apart from studying the effect of *HTT* mutation status on DNAm levels, we also tested the hypothesis that methylation levels in blood-derived DNA are predictive of motor progression in manifest HD cases. We analyzed large-scale longitudinal motor score assessments from (1) Enroll-HD data 1 after the initial blood draw (on average 3.1 years of follow-up; Supplementary Table 2), (2) the Enroll-HD data 2, which only involved HDGECs (on average 7.9 years apart from the first blood draw; Supplementary Table 3), and (3) the last visit in the Registry-HD cohort (Table 1). Our EWAS of HD progression involved 917 individuals with manifest HD for whom both DNAm profiles and longitudinal clinical assessments were available (“Methods”).

### HD motor progression in manifest HD cases

**Fig. 1:**
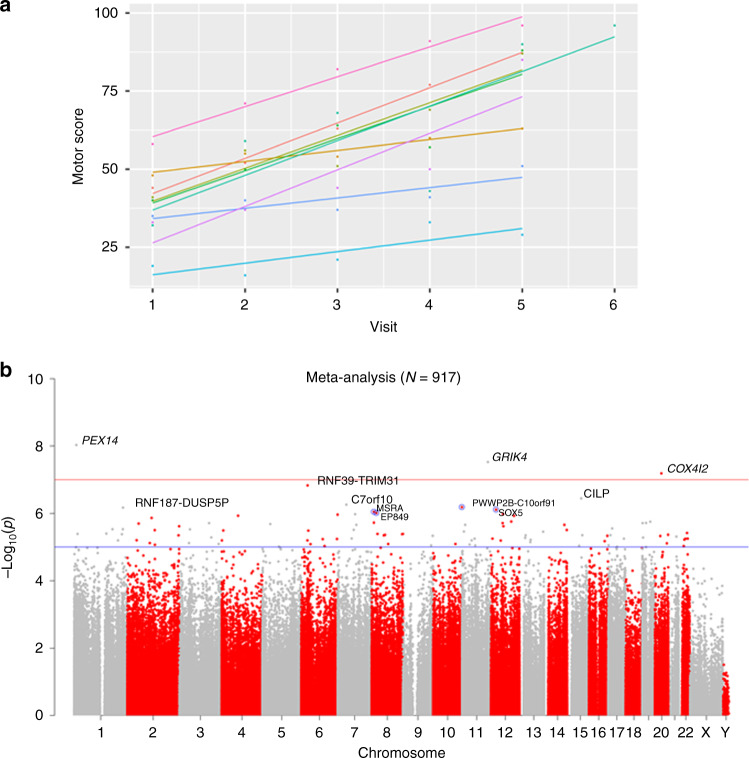
EWAS of motor progression in manifest HD cases. **a** The spaghetti plot illustrates how the motor score depends on the visit in Enroll-HD. To arrive at a measure of motor score progression (i.e., the rate of change in the motor score), we estimated the slope using a random-effects model with a random slope and intercept term. **b** The Manhattan plot visualizes the EWAS results of motor progression in manifest HD cases: log (base 10)-transformed meta-analysis *p* value (*y*-axis) versus the chromosomal location of each CpG (*x*-axis). The EWAS results were calculated with the biweight midcorrelation tests^50^. Fixed-effects meta-analysis (weighted by inverse variance) was performed to combine the EWAS results across blood data (*N* = 917) from Enroll-HD data 1, Enroll-HD data 2, and Registry data. All *p* values are two-sided and not adjusted for multiple comparisons. The blue and red horizontal lines correspond to suggestive significance (*α* = 1.0 × 10^−5^) and genome-wide significant levels (*α* = 1.0 × 10^−7^), respectively. Gene names are provided for CpGs (blue circles) with *p* < 1.0 × 10^−6^ with detailed summary statistics reported in Supplementary Data 3.

A linear mixed analysis was performed to define a measure of HD motor progression in manifest patients from Enroll-HD. For data 1, we used a large-scale Enroll-HD database comprising 14,850 longitudinal observations across 5204 manifest patients (“Methods”). Total motor scores were measured by the Unified Huntington’s Disease Rating Scale (UHDRS)^12^, a standard clinical assessment to quantify the severity of disease (Supplementary Note 1). A higher UHDRS motor score indicates a more severe disease progression. Figure 1a illustrates the heterogeneity of slopes of longitudinal motor scores versus visit, indicating the utility of modeling visit as a random effect in a linear mixed-effects model. A linear mixed-effects model was used to relate the longitudinal motor scores (dependent variable) to visit (random effect), age, sex, CAG length, age at motor onset, and educational attainment (“Methods”). Our measure of HD progression was defined by the random slope estimate after adjusting for the other covariates. The *adjusted* random slope estimate can be viewed as a measure of atypical HD progression not predicted by *HTT* mutation, age, and potential confounders. This measure is analogous to the one used in an earlier genome-wide association study (GWAS) of HD progression^13^.

After >2 years of follow-up, motor scores were substantially increased (on average 7.6 points; Supplementary Fig. 1). As expected, CAG-repeat length and AMO were significantly associated with an increase in motor score in a linear mixed model analysis (Table 2). The model revealed that motor scores were increased by 2.7 ± 0.09 (*p* = 5.3 × 10^−191^) per one unit increase in CAG-repeat length, increased by 2.2 ± 0.05 (*p* < 5.0 × 10^−300^) per year (of age), increased by 3.5 ± 0.08 (*p* < 5.0 × 10^−300^) per year of follow-up, and decreased by 1.54 ± 0.05 (*p* = 1.24 × 10^−201^) per year of AMO. Females were associated with higher motor scores (*p* = 1.16 × 10^−12^) and years of education were associated with lower motor scores (*p* = 3.31 × 10^−22^).

**Table 2:** Linear mixed model regression analysis for HD motor progression.

Parameter	Outcome: motor score				Outcome: random slope			
	Coef.	SE	*T* statistic	*P* value	Coef.	SE	*T* statistic	*P* value
Female	3.375	0.474	7.127	1.16 × 10^−12^	0.016	0.051	0.316	0.8
Age	2.217	0.045	48.878	<5.0 × 10^−300^	0.013	0.005	2.749	5.99 × 10^−3^
CAG length	2.743	0.089	30.757	5.27 × 10^−191^	0.071	0.010	7.449	1.09 × 10^−13^
Age at motor onset	−1.542	0.049	−31.687	1.24 × 10^−201^	−0.010	0.005	−2.006	4.49 × 10^−2^
Education	−1.877	0.193	−9.742	3.09 × 10^−22^	−0.083	0.021	−4.037	5.49 × 10^−5^
Visit	3.516	0.078	45.019	<5.0 × 10^−300^				

The table presents the coefficient estimates from two linear regression models: (1) a linear mixed model analysis of 14,850 longitudinal motor scores (dependent variable) across 5204 manifest HD cases from Enroll-HD data 1 and (2) a linear regression model analysis of the resulting random slope estimates (dependent variable) of the same 5204 cases. The linear mixed-effects model included two random-effects (a random intercept term and a random slope term with respect to visit) and several fixed-effect terms (sex, age at baseline, CAG-repeat length, age at motor onset, educational attainment). The empirical Bayes estimate of the random slope was used as a measure of HD motor progression for each of the *N* = 5204 manifest HD cases. We adjusted the random slope estimate for all fixed effects in our downstream EWAS analysis. The columns report the covariate name, regression coefficient, standard error, Student’s *T* statistic, and unadjusted two-sided Wald test *p* value.

A linear mixed analysis was analogously performed in Enroll-HD data 2 (1867 observations across *N* = 278 patients, with a longer follow-up period (“Methods” and Supplementary Table 4). The motor score in this group at the last visit showed a substantially larger increase (on average 27.43 compared to 7.6), due to the longer follow-up period (on average 7.8 years compared to 2 years; Supplementary Fig. 1). Both linear mixed-effects models reveal that our measures of HD motor progression (random slope) are significantly associated with age and CAG-repeat length in both Enroll-HD data 1 and 2 (Table 2 and Supplementary Table 4, respectively). CAG-repeat length exhibits particularly significant associations with HD progression (*p* = 1.1 × 10^−13^ in data 1 and *p* = 1.2 × 10^−7^ in data 2).

In the Registry-HD data, we used a different definition for motor progression because it differed from Enroll-HD in terms of the assessment of follow-up information. Short-term follow-up information (on average 1.18 ± 0.6 years) was available for 224 out of 287 manifest patients from the Registry-HD study. Similar to a previous study of motor progression in the Registry-HD data^13^, we defined progression as raw residual resulting from a linear model that regressed the motor score at the last visit (dependent variable) on age and other determinants of motor progression (“Methods”). The Registry-HD data showed comparable patterns for the associations of the fixed effects with motor scores in terms of directions of regression coefficients and effect sizes (Table 2 and Supplementary Table 5).

### HD manifest patients exhibiting accelerated blood epigenetic aging effects

We applied five human epigenetic biomarkers of aging (epigenetic clocks) that lend themselves to analyzing blood methylation data: (1) Horvath’s pan-tissue epigenetic age (referred to as DNAm age^14^); (2) Hannum’s blood-based DNAm age^15^; (3) skin and blood clock (DNAmAgeSkinClock)^16^; (4) DNAmPhenoAge^17^; and (5) the mortality risk estimator DNAmGrimAge^18^.

**Fig. 2:**
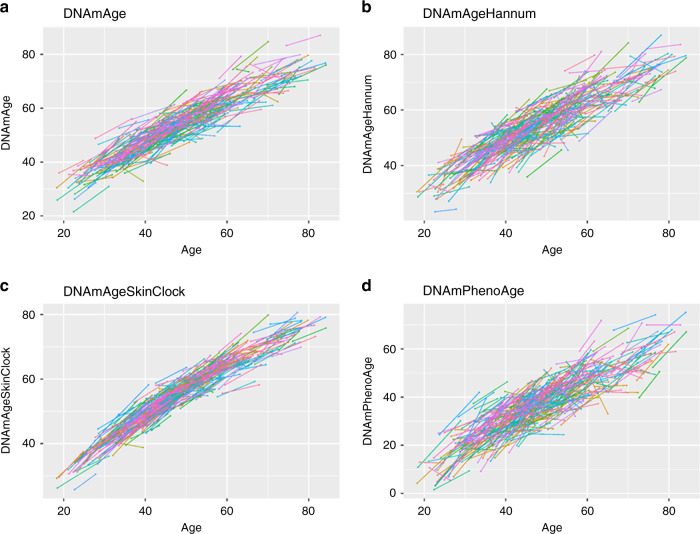
Spaghetti plots of DNA methylation age versus chronological ages in the longitudinal Enroll-HD data 2. Each spaghetti plot depicts DNAm age versus chronological age, using the Enroll-HD data 2 with two time-points per individual. Each line connects two blood draws from the same individual collected on average 7.9 years apart. The panels report results for different epigenetic clocks **a** Horvath’s pan-tissue clock^14^, **b** Hannum’s blood-based clock^15^, **c** the skin and blood clock^16^, and **d** and DNAm PhenoAge^17^. The same analysis was not performed on DNAmGrimAge because it is a mortality risk predictor that uses age in its definition.

Using the longitudinal Enroll-HD data 2, we found that all DNAm age estimators track the passage of study time (Fig. 2a–d). In the cross-sectional data, the correlation between DNAm age estimators and chronological ages were >0.84, led by DNAmAgeSkinClock (Fig. 2c) with correlation estimates ranging between 0.95 and 0.97 across all the three datasets (Supplementary Figs. 2e, f, 3c, and 4c).

**Fig. 3:**
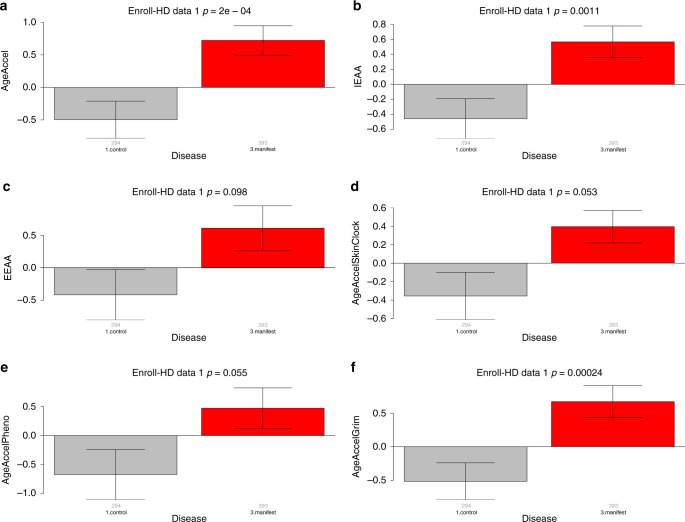
Epigenetic age acceleration in blood versus manifest HD disease status. Epigenetic age acceleration (*y*-axis) versus manifest HD status in *N* = 687 (294 controls and 393 manifest HD) blood samples from Enroll-HD data 1. Each panel corresponds to a different epigenetic clock. **a** Age-adjusted DNAm age based on Horvath’s pan-tissue clock (AgeAccelerationResidual [AgeAccel])^14^, **b** intrinsic epigenetic age acceleration (IEAA), which is independent of blood cell composition, **c** extrinsic epigenetic age acceleration, which is based on Hannum’s clock and does depend on blood cell composition^15^, **d** age-adjusted DNAm age based on the skin and blood clock (AgeAccelSkinClock)^16^, **e** age-adjusted DNAm PhenoAge (AgeAccelPheno)^17^, and **f** age-adjusted DNAm GrimAge^18^ (AgeAccelGrim). The title of the bar plots reports the nominal two-sided *p* value from a Kruskal–Wallis test. The *y*-axis of the bar plots depicts the mean and one standard error. Group sizes (i.e., number of individuals per disease category) can be found as small gray numbers underneath each bar.

To formally measure possible epigenetic AgeAccel effects due to HD disease status, we fit a regression model of DNAm age on chronological age and defined AgeAccel as the resulting raw residual. Thus, positive AgeAccel means the methylation state of the sample appears to be older than would be expected based on chronological age. We found that manifest HD (but not pre-manifest HD) had significantly higher epigenetic AgeAccel than controls (0.098 ≤ *p* ≤ 2.0 × 10^−4^, Fig. 3), led by the pan-tissue clock (AgeAccel, *p* = 2.0 × 10^−4^, Fig. 3a) and AgeAccelGrim (*p* = 2.4 × 10^−4^; Fig. 3f). The difference remained significant (*p* = 1.1 × 10^−3^; Fig. 3b) when studying the measure of intrinsic epigenetic AgeAccel (IEAA), which is independent of imputed blood cell counts^19^. Despite the low number of controls (*N* = 78), we managed to corroborate the finding for AgeAccelGrim in the Registry-HD data (*p* = 0.046; Supplementary Fig. 5L).

A multivariate model analysis demonstrates that the observed epigenetic AgeAccel effects are not due to confounders (Supplementary Table 6). In particular, we find that manifest HD is associated with a significant increase in DNAm age (*p* = 2.9 × 10^−3^) in the Enroll-HD data even after adjusting for the 10 principal components. However, this association could not be replicated in the Registry-HD data (Supplementary Table 7).

The pan-tissue clock also revealed a strong correlation between lymphoblastoid cells blood (*r* = 0.74, *p* = 2.1 × 10^−18^; Supplementary Fig. 6A), but the DNAm Age estimate was significantly lower than that of corresponding blood samples (mean difference = 3.3 years, *p* = 0.0017; Supplementary Fig. 6B), suggesting that the Epstein*–*Barr virus transformation used in generating lymphoblastoid cell lines decreases DNAm age.

Next, we tested whether epigenetic AgeAccel is associated with motor progression in manifest HD. The considered measures of epigenetic AgeAccel exhibit nominally significant positive correlations with motor progression (e.g., *r* = 0.08, meta-analysis *p* = 0.016 for the pan-tissue clock; Supplementary Table 8 and Supplementary Fig. 7).

### Human EWAS of HDGEC status

**Fig. 4:**
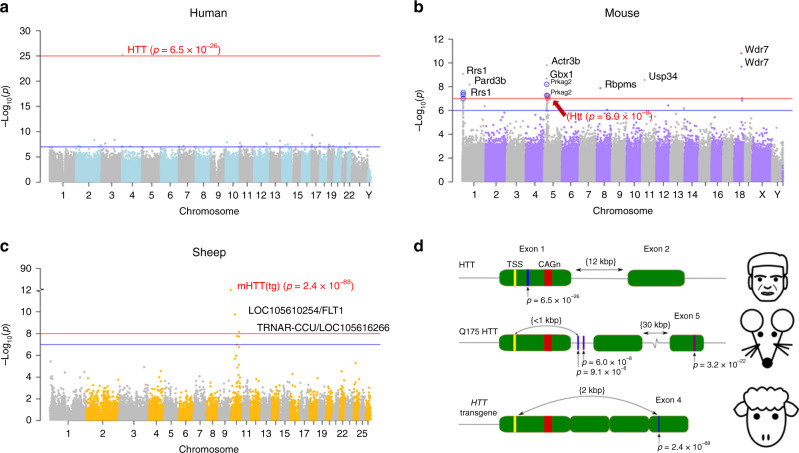
Epigenome-wide association study (EWAS) of HD status in three species. HDGEC status (carrier versus control) was related to DNA methylation data in three different species: **a** human blood, **b** mouse striatum/cerebellum, and **c** sheep blood. Each Manhattan plot reports minus log_10_-transformed *p* values (*y*-axis) versus chromosomal location. Each dot corresponds to a CpG. CpGs near *HTT* are marked in red. Human *HTT* is located in chromosome 4. Mouse Q175 knock-in in Htt is located on mouse chromosome 5. Sheep transgenic gene HTT is located on sheep chromosome 10. The blue horizontal lines correspond to genome-wide significance levels for **a**, **c** (*α* = 1.0 × 10^−7^) and a suggestive significance level for **b** (1.0 × 10^−6^); the red horizontal lines correspond to *p* < 1.0 × 10^−32^, *p* < 1.0 × 10^−7^, and *p* < 1.0 × 10^−8^ for **a–c**, respectively; the break in the *y*-axis indicates where the level of significance has been truncated for the purpose of visualization. **b** CpGs marked in blue on chromosome 1 are located in *Ryrs1*. CpGs marked in red and blue on chromosome 5 are located in *Htt* and *Prkag2*, respectively. **d** The alignment of significant CpGs (blue vertical lines) in the human (hg38), mouse (mm10) and sheep (oviAri4) *HTT* locus. The significant *HTT*/*Htt* CpGs (*p* < 1.0 × 10^−7^) are colored in blue with *p* values displayed below for human, mouse (RRBS methylation array), and sheep EWAS, respectively. The most significant CpG found using the mouse methylation array data are colored in magenta.

In our EWAS, we related HDGEC status to individual epigenetic markers (CpGs) using Enroll-HD data 1 and Registry-HD, then combined the results using the Stouffer’s method. To avoid technical confounding by chip effects in the Enroll-HD data 1, we used the aggregated Enroll-HD data for our EWAS analysis (Supplementary Table 9). The association between HD and individual methylation levels is strongly preserved across the two datasets (*r* = 0.31; Supplementary Fig. 8). The Manhattan plot reveals that CpG cg22982173 in exon 1 of the *HTT* gene on chromosome 4 was most significantly associated with HD status (Stouffer’s *p* = 6.5 × 10^−26^; Fig. 4a and Supplementary Fig. 9).

We set up a significance threshold at *α* = 10^−7^ to adequately control for false-positive rate in the DNAm array studies^20^. We found 33 genome-wide significant CpGs for HD status (Table 3 and Supplementary Data 1), including cg22982173 in *HTT* (*p* = 7.0 × 10^−26^), cg26892702 near *DVL2* (*p* = 5.3 × 10^−10^), cg23819669 near *DNAJB8* (*p* = 4.2 × 10^−9^), cg19759282 near *GPR155* (*p* = 4.4 × 10^−9^), cg16739503 near *RASA3* (*p* = 6.5 × 10^−9^), cg20684718 near *MCM10* (*p* = 1.4 × 10^−8^), cg22296756 near *HDAC4* (*p* = 6.9 × 10^−8^), and probe ch.3.75336R near *MLH1* (*p* = 2.4 × 10^−8^).

**Table 3:** Meta-analysis EWAS of HDGEC status in human blood.

Chr.	CpG	bp	CpG island	Gene	Gene location	Meta *p*	Meta *Z*
4	cg22982173	3,076,557	Island	*HTT*	First exon	6.5 × 10^−26^	10.53
17	cg26892702	7,132,489		*DVL2*	Body	5.3 × 10^−10^	−6.21
3	cg23819669	128,187,274		*DNAJB8*	TSS1500	4.2 × 10^−9^	−5.88
2	cg19759282	175,351,649	Island	*GPR155*	5′-UTR; 1st exon	4.4 × 10^−9^	5.87
13	cg16739503	114,862,324	S_Shore	*RASA3*	Body	6.5 × 10^−9^	−5.8
10	cg20684718	13,248,241		*MCM10*	Body	1.4 × 10^−8^	−5.68
12	cg04605980	117,471,700				1.8 × 10^−8^	−5.63
11	cg06993329	886,695	N_Shore	*CHID1*	Body	1.9 × 10^−8^	−5.63
17	cg04683330	80,041,239	Island	*FASN*	Body	1.9 × 10^−8^	−5.62
12	cg16320626	121,877,851	N_Shelf	*KDM2B*	Body	2.1 × 10^−8^	−5.6
3	ch.3.753362R^a^	37,048,044		*MLH1*	5′-UTR; body	2.4 × 10^−8^	5.58
16	cg09080920	928,338	Island	*LMF1*	Body	2.5 × 10^−8^	−5.57
22	cg14703589	31,644,558		*LIMK2*	First exon; 5′-UTR; body	2.6 × 10^−8^	−5.57
12	cg17859634	124,798,815	Island	*FAM101A*	Body	3.4 × 10^−8^	−5.52
14	cg04285477	74,194,516		*C14orf43*	Body	3.6 × 10^−8^	−5.51
17	cg23148992	3,558,246		*CTNS*	Body	4.1 × 10^−8^	−5.49
19	cg08117431	5,455,497	Island	*ZNRF4*	5′-UTR; 1st exon	4.2 × 10^−−8^	−5.48
2	cg15115604	241,069,379	Island	*MYEOV2*	Body	5.0 × 10^−8^	−5.45
14	cg10929299	50,334,860				5.1 × 10^−8^	5.45
19	cg10585870	14,530,314	Island	*DDX39*	TSS200	5.6 × 10^−8^	5.43
11	cg15746396	13,485,203	Island	*BTBD10*	TSS1500	6.2 × 10^−8^	5.41
7	cg14640310	44,058,913	Island	*POLR2J4*	TSS200	6.9 × 10^−8^	5.39
2	cg22296756	240,305,369		*HDAC4*	5′-UTR	6.9 × 10^−8^	−5.39
19	cg11543665	8,373,294	Island	*CD320*	TSS201	6.9 × 10^−8^	5.39
2	cg05054944	25,856,333		*DTNB*	Body	7.2 × 10^−8^	−5.39
16	cg00284420	87,311,948				7.5 × 10^−8^	−5.38
17	cg24965497	7,227,181	N_Shore	*NEURL4*	Body	7.6 × 10^−8^	−5.38
4	cg13731523	3,047,190	S_Shelf			8.0 × 10^−8^	−5.37
17	cg14517001	80,179,931	Island			8.2 × 10^−8^	−5.36
19	cg20635595	48,281,627	Island	*SEPW1*	TSS1500	8.4 × 10^−8^	5.36
11	cg16356630	14,683,035		*PDE3B*	Body	8.7 × 10^−8^	−5.35
4	cg01200289	77,227,751	Island	*STBD1*	First exon; 5′-UTR	9.0 × 10^−8^	5.35
12	cg11881599	92,814,084		*CLLU1OS*	3′-UTR; TSS1500	9.2 × 10^−8^	5.34

The table reports 33 genome-wide significant CpGs (meta-analysis *p* value <10^−7^). A positive (negative) value of the meta-analysis *Z* statistic indicates that the CpG is hypermethylated (hypomethylated) in human HDGECs. In this case–control study, we did not distinguish pre-manifest from manifest HD. The *Z* statistic is based on Stouffer’s meta-analysis method across the aggregated Enroll-HD samples and the Registry-HD data. Columns report the chromosome, CpG, position (hg19 assembly), the relative location to the nearest CpG island, nearest gene, location of the CpG with respect to the gene, meta-analysis (unadjusted, two-sided) *p* value, and *Z* statistic.

^a^The unusual probe name “ch.3.753362R” reflects a CpH probe (CA, CC, CT) as opposed to a CpG probe, which targets the reverse strand on chromosome 3 (hg19 coordinate 753362).

### HTT methylation across multiple brain regions

**Fig. 5:**
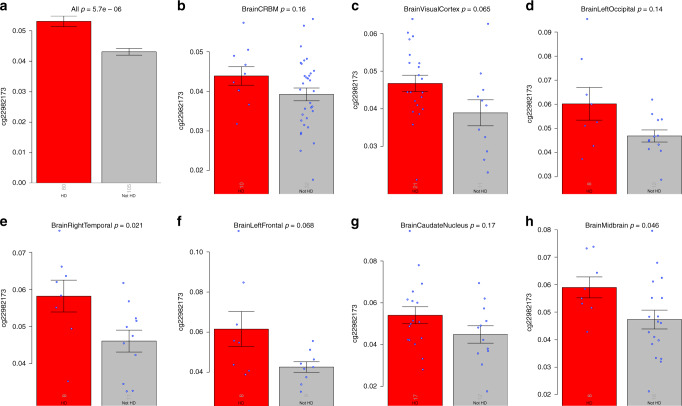
Human HTT methylation in different brain regions. The figure depicts the bar plots for the association of HD disease status with *HTT* cg22982173 in different brain regions (*N* = 67 from 26 HD versus 39 control individuals). We used *n* = 185 brain tissues (80 HD versus 105 controls) from seven brain regions: **a** bar plot based on all the seven brain regions combined. The results for **a** remain significant (*p* = 0.015) after adjusting for intra-individual correlations using a linear mixed-effects model. **b**–**h** Bar plots for each individual region: cerebellum (CRBM), visual cortex, left occipital, right temporal cortex, left frontal cortex, caudate nucleus, and midbrain, respectively. The blue dots correspond to individual observations that underlie the respective mean values. The *y*-axis of the bar plots depicts the mean of one standard error. The numbers of individuals per group are reported as gray numbers in each bar. The heading of each panel reports a nominal (unadjusted) two-sided *p* value (Kruskal–Wallis test).

We studied *HTT* cg22982173 using 475 brain methylation samples from our previous study (26 HD versus 39 control individuals; “Methods”)^6^. Figure 5 lists the bar plots for the seven selected brain regions, with right temporal cortex (*p* = 0.02; Fig. 5e) and midbrain (*p* = 0.046; Fig. 5h) reaching statistical significance for increased methylation at the *HTT* cg22982173 locus in HD cases compared to controls. Methylation levels of cg22982173 across all brain regions combined were significantly higher in the HD group (Fig. 5a: *p* = 5.6 × 10^−6^ and remained significant, *p* = 1.5 × 10^−2^, after adjusting for intra-subject dependence).

Our top EWAS hit in the blood (in the *HTT* locus) continues to be associated with HD status in several brain regions (Fig. 5), while the EWAS results in brain tissue are different from those of blood tissue on a global level (Supplementary Fig. 13). Supplementary Figures 11 and 12 report bar plots of cg22982173 stratified by study and tissue types: blood, lymphoblastoid cell, fibroblast, and a total of 14 brain regions. The CpG cg22982173 resides within a region of the genome with a high density of CpGs (i.e., in a CpG island) and therefore would be expected to have low levels of methylation; however, significant increases in methylation were observed in HD cases.

### Both CAG-repeat alleles correlate with human *HTT* methylation

CAG-repeat lengths were assessed for both long and short alleles (CAG.long and CAG.short) in the human *HTT* exon 1 locus. We correlated both alleles to human *HTT* methylation (at cg22982173) using all *N* = 2164 human samples. A series of multivariate regression models were tested to evaluate the joint effect of CAG.long and CAG.short in (i) all, (ii) controls only, and (iii) HD mutation carriers only, respectively (“Methods”).

Strikingly, our comprehensive model fitting analysis in different data and cell types demonstrated that both CAG.long and CAG.short were independently associated with *HTT* methylation (Supplementary Data 2). These significant associations were observed even after restricting the analysis to controls or to HD mutation carriers, respectively. Interestingly, the *product* of the two CAG-repeat lengths (CAG.product = CAG.long × CAG.short) showed the best relationship with *HTT* methylation according to the Bayesian information criterion (BIC) (Supplementary Data 2). The CAG.product model exhibited the best fit (lowest BIC value) when considering all *N* = 910 blood samples from the Enroll-HD data 1, all *N* = 376 blood samples from the Registry-HD, *N* = 294 controls from Enroll-HD data 1, *N* = 616 HD mutation carriers from Enroll-HD data 1, the *N* = 298 HD mutation carriers from Registry-HD, *N* = 80 mutation carriers from the lymphoblastoid data, and *N* = 37 mutation carriers from the fibroblast data.

The quadratic term in CAG.long was also significantly related to *HTT* methylation using polynomial modeling analysis. The multivariate linear models showed that age and sex only have negligible effects on the methylation levels of cg22982173 (Supplementary Data 2).

**Fig. 6:**
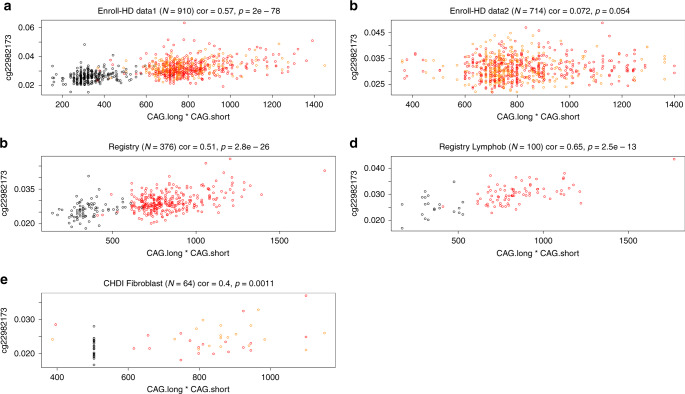
Human HTT methylation versus the product of CAG lengths. The product of the two CAG alleles (CAG.product = CAG.long × CAG.short) correlated with DNA methylation levels of the human *HTT* cg22982173 locus in **a** blood samples from Enroll-HD data 1, **b** blood samples from Enroll-HD data 2, **c** blood samples from the Registry-HD study, **d** lymphoblastoid lines from the Registry-HD study, and **e** fibroblast samples from the MTM study. The title of each panel reports the Pearson’s correlation coefficient and corresponding unadjusted *p* value. Fixed-effects meta-analysis (weighted by inverse variance) produced a meta-analysis *p* value of 9.1 × 10^−123^. Points in the scatter plots are colored by HD status: red = manifest HD, orange = pre-manifest HD, and black = control. Each plot reports a Pearson’s correlation coefficient and corresponding *p* value.

The moderately high correlation estimates (*r* ~ 0.4–0.65) between the CAG.product and *HTT* methylation levels were replicated in several datasets (meta-analysis *p* value of 9.1 × 10^−123^; Fig. 6). Significant correlations can be observed even when using only controls (meta-analysis *p* = 4.5 × 10^−8^) or only HD mutation carriers (*p* = 3.9 × 10^−27^; Supplementary Figs. 14 and 15).

The methylation levels of cg22982173 were low both in HD cases and controls: the *β* value (interpreted as the proportion of chromosome methylated at this locus) ranged from 0.015 to 0.063 (Supplementary Fig. 16).

### Human *HTT* methylation is not confounded by other trinucleotide tandem repeats

It is well known that the HD expansion is overrepresented on a subset of the haplotypes associated with non-disease-associated alleles. Notably, a potential methylation-modifying polymorphism exists in the form of the polymorphic CCG repeat, which lies immediately downstream of the CAG repeat^21^ and tags many of the most common *HTT* haplotypes.

We evaluated five multivariate linear regression models to examine if the association between *HTT* cg22982173 and CAG length could possibly arise as an artifact of linkage disequilibrium between the CAG repeat and some other methylation-modifying variants at the *HTT* locus (“Methods”). Especially, we examined the model (Model 5) that omitted all HD cases that carried an atypical *HTT* structure, defined as those that do not conform to the “typical” reference alleles (CAG)_Q1_CAACAGCCGCCA(CCG)_P2_(CCT)_2_ (where Q1 and P2 are numbers of pure CAGs and CCGs, respectively)^22^. We studied 372 individuals from the Registry-HD cohort, which revealed a marked association between CAG- and CCG-repeat lengths in the Registry-HD cohort (*r*^2^ = 0.16, *p* < 2 × 10^−16^; Supplementary Fig. 17A). However, several lines of evidence suggest that the most associations we have observed in *HTT* cg22982173 are not driven by CCG length or other HD-specific haplotype effects, but are a direct product of the CAG length (linear models 1–5 listed in Supplementary Table 10).

### Human EWAS of motor progression in manifest HD

To test whether *HTT* methylation matters for motor progression in manifest HD cases, we performed a meta-analysis that combined three separate EWAS of (adjusted) motor progression using the two Enroll-HD and the Registry-HD datasets, respectively (“Methods”). Interestingly, higher methylation of the *HTT* locus (cg22982173) was associated with slower HD progression in manifest HD cases according to the motor score (*p* = 0.05), Stroop tests for cognitive function assessment (color naming *p* = 0.01, word reading *p* = 0.07, and interference test *p* = 0.06), and functional assessment score (*p* = 0.009).

Our blood EWAS of HD progression in manifest HD cases found three genome-wide significant CpGs: cg26919387 (meta-analysis *p* = 9.3 × 10^−9^) in *PEX14*, cg12823408 near *GRIK4* (meta-analysis *p* = 3.0 × 10^−8^), and cg21497164 in *COX4I2* (meta-analysis *p* = 6.5 × 10^−8^; Fig. 1b, Supplementary Fig. 18, Supplementary Table 11, and Supplementary Data 3). The robust correlation (approximate *r* = −0.19) indicated that all three CpGs that were hypomethylated were associated with HD motor progression.

### The top EWAS hits were not confounded by single-nucleotide polymorphisms (SNPs)

CpG measurement can be compromised by neighboring SNPs. We addressed this concern using two approaches. First, we correlated the significant CpGs with SNPs that located near the CpG probes according to the Illumina annotation file. No significant correlations were observed.

Second, we applied the software Gaphunter^23^ and found only 1 CpG (cg04285477 near *Cl4orf43*) out of 33 EWAS CpGs associated with HD disease status exhibited weak evidence of confounding (i.e., a clustering pattern; “Methods”). None of the three CpGs (top hits) associated with motor progression showed evidence of confounding.

### EWAS of motor progression was not confounded by *treatment*

Tetrabenazine, a medication for symptomatic treatment of chorea, was used by ~23% of HD patients in Enroll-HD. Treated and untreated HD patients did not differ in terms of age, but treated patients exhibited a higher CAP score (*p* = 0.01 in Enroll-HD data 1 and *p* = 8.2 × 10^−8^ in Enroll-HD data 2) and a higher motor score (*p* = 3.2 × 10^−4^ in Enroll-HD data 1 and *p* = 2.1 × 10^−9^ in Enroll-HD data 2; Supplementary Figs. 19 and 20). Tetrabenazine was not significantly associated with any of the three motor progression-associated CpGs as depicted in Supplementary Fig. 21. Furthermore, no CpGs on the Illumina array were associated with tetrabenazine treatment at a genome-wide significance level of *p* = 1.0 × 10^−7^ (Supplementary Fig. 22). Overall, these results demonstrate that the EWAS of HD progression is not confounded by tetrabenazine treatment.

### Methylation data from *Htt* knock-in mice (Q175)

We generated two types of DNAm datasets (RRBS and custom methylation array) from a heterozygous (HET) *Htt* knock-in (KI) mouse model (Q175 versus Q20 CAG repeats or wild-type mice; “Methods”).

We conducted EWAS of mutant *HTT* gene (Q20 versus Q175) across the striatum and cerebellum in RRBS data. Focusing on CpGs in the murine *Htt* locus revealed a moderately strong correlation between the striatum and the cerebellum (*r* = 0.55 and *p* = 5.3 × 10^−11^; Supplementary Fig. 23). Strikingly, CpG sites falling within the *Htt* locus are among the top EWAS results for Q175 status in murine striatum and cerebellum (Supplementary Fig. 24). This finding is also illustrated by our meta-analysis EWAS across the striatum and cerebellum (Fig. 4b). Our EWAS identified two genome-wide significant CpGs in *Htt* region, chr5:34762314 (meta-analysis *p* = 6.0 × 10^−8^) and chr5:34762241 (*p* = 9.2 × 10^−8^) located between exons 1 and 2 (Fig. 4b, d and Supplementary Data 1). These CpGs were located ~1 kb in the 3′ direction of *HTT* exon1 (Fig. 4d), both were hypermethylated in the Q175 group.

Analyzing the same brain regions from another group of mice using our custom array platform revealed another highly significant CpG to be hypomethylated in the Q175 group (probe cg12389415 on the custom array, mouse chr5:34795955) also within the *Htt* locus (meta-analysis *p* = 3.2 × 10^−22^; Supplementary Fig. 25). Integrating the EWAS results across all the available tissue types: the two brain regions, blood, cortex, and liver, increased the statistical significance of the association (Stouffer meta-analysis *p* = 6.2 × 10^−45^). The *Htt* gene locus was the top EWAS hit in all five tissues profiled on the custom methylation array (Supplementary Data 1).

### Ovine EWAS of HD status

In addition to mice, we also studied a transgenic model of sheep with a human *HTT* cDNA transgenic sequence^24–28^. The transgene is integrated into a different location than the endogenous sheep *HTT* gene. Using a custom methylation array, we generated blood methylation data from 168 sheep: 84 HD transgenic sheep and age-matched controls; “Methods”). EWAS of HD transgenic status identified five genome-wide significant (*p* < 1.0 × 10^−7^) CpGs located on chromosome 10 including one CpG near the *HTT* transgene (Fig. 4c, according to oviAri4 assembly coordinates 1826–1903 bp). The most significant association was the CpG located in exon 4 of the *HTT* transgene with a striking *p* value of 2.4 × 10^−88^, followed by another CpG in the *FLT1* gene (*p* = 1.8 × 10^−10^; Supplementary Data 1).

### Only CpGs close to the CAG expansion are hypermethylated in HD

A comparison of the significant CpGs in the *HTT* locus across three species, human, mouse and sheep (Fig. 4d), revealed that significant CpGs for HD status are located within 2 kb of the CAG expansion in exon 1 of *HTT*/*Htt*. The comparison between human and sheep highlighted that the proximity between a CpG dinucleotide and the location of the CAG expansion in either the endogenous *HTT* or transgenic *HTT* (typically <2 kb) plays an important role (Fig. 4d). This is based on the following observations: (a) all genome-wide significant CpGs near the *HTT* locus are located within 2 kb of the CAG expansion, (b) unlike in humans, *HTT* exon 4 is close to the site of the CAG expansion in the sheep because the *HTT* transgene is a cDNA without any intronic genomic DNA, (c) unlike in humans, methylation at a specific CpG in exon 4 of an *HTT* cDNA gene fragment relates strongly to HD transgenic gene status in blood samples from the sheep (Supplementary Data 1).

Next, we investigated if HD patients tended to gain methylation in associated *HTT* CpGs. Interrogating the genomic regions surrounding *HTT* exon 1, all four CpGs were significantly hypermethylated in HD patients (Supplementary Data 4). Interestingly, all the 27 significant CpGs in *Htt* (meta-analysis *p* < 0.05) also exhibited the same hypermethylation pattern in Q175 mice (Supplementary Data 4).

### Enrichment analysis of EWAS results

To gain insights on the biological function associated with HD related CpGs, we used two different approaches for carrying out functional enrichment analysis: (1) the anRichment approach under HDinHD^29^ and (2) GOMETH enrichment analysis, which takes into account the different number of probes per gene present on the Illumina 450k array^30^. We studied the top 1000 CpGs that were *hypo*methylated in HD and another set of top 1000 CpGs that were *hyper*methylated in HD using the anRichment analysis (Supplementary Tables 12 and 13). The top 1000 hypomethylated CpGs were adjacent to genes (hypergeometric *p* = 3.6 × 10^−13^) implicated by a protein–protein interaction network analysis of polyglutamine disorders (Supplementary Table 14). The most significant enrichment according to the GOMETH analysis involved the overlap between the top 1000 hypermethylated CpGs and RNA binding (*p* = 3.4 × 10^−6^; Supplementary Table 15).

We carried out an enrichment analysis of CpGs associated with HD progression using the same two approaches. The anRichment analysis identified the HD progression related genes enriched in several known HD relevant gene sets (Supplementary Table 16) and the GOMETH analysis identified gene sets enriched in immune cell activation (Supplementary Table 17).

## Discussion

This is by far the largest DNAm study of HD to date. We generated large methylation datasets from seven sources of DNA across three species (human: 2000 blood, 100 lymphoblastoid cells, and 64 fibroblasts; mouse: 32 striatum, 32 cerebellum, 16 liver, 16 cortex, and 16 blood; sheep: 168 in the blood).

One of our major findings is that manifest (but not pre-manifest) HD cases exhibit accelerated epigenetic aging effects in blood, which is consistent with the epigenetic AgeAccel observed in various brain regions^6^. HD progression in manifest individuals was only weakly linked to epigenetic AgeAccel in blood.

A second major finding of our study was that EWAS (as opposed to GWAS) of the *HTT* mutation status consistently found CpGs in the *HTT* locus in all species and tissues. If nothing was known about the genetics of HD, then our human blood methylation data would have implicated the pertinent disease gene: a very significant CpG probe located in *HTT* exon 1. The *HTT* methylation effect was validated across seven sources of DNA (blood, lymphoblastoid cells, fibroblasts, striatum, cerebellum, cortex, and liver) and across three species. Our most significant human *HTT* cg22982173 was neither confounded by neighboring trinucleotide tandem repeats nor dominated by atypical *HTT* structure. We also analyzed custom methylation array data (providing >1000× coverage per CpG) to address concerns surrounding low sequence coverage (minimum 15× coverage) in the mouse RRBS data^31^. Both mouse RRBS and custom methylation array data implicated the *Htt* gene. A comparison of the significant CpGs in the *HTT*/*Htt* locus across three species—human, mouse, and sheep—revealed that significant CpGs for HD status were located in the vicinity of the CAG expansion in exon 1 of *HTT/Htt*. The methylation levels of cg22982173 exhibited only weak (but nominally significant) negative correlations with measures of motor progression and other clinical assessments (e.g., Stroop test) in manifest HD after adjusting CAG expansion, and other potential confounders. An intriguing and yet unexplained finding, based on our comprehensive model fitting analysis of the most significant CpG cg22982173 in the human *HTT* locus, was that the methylation level at this locus correlated with the allelic product of CAG lengths in both cases and controls, inferring that methylation at this locus was related to the length of the non-mutated allele as well. Our analysis of *HTT* methylation in human postmortem brain samples was consistent with that observed in blood samples.

Abundant caution needs to be exercised in linking *Htt* exon1 methylation to expression levels of the mutant *HTT/Htt* gene, since CpGs often have only weak effects on neighboring gene expression levels^32^. However, it is interesting that at least in the murine *Htt* allelic series KI mouse model, there is a robust *Htt* CAG-length-dependent reduction of mutant *Htt* RNA and protein levels in the striatum^29^. Caution is also warranted when interpreting the relationship between transgene expression and CpG methylation in the transgenic sheep model for the following reasons. When exogenous DNA is inserted into a host genome in order to generate a transgenic cell or animal model, copy number, integration site, and transgene composition (e.g., the inclusion of exogenous viral or bacterial sequences) could all significantly influence the status of transgene methylation and expression^33,34^. Moreover, transgenes can be considerably affected by the flanking genomic DNA sequences at its random integration site, hence susceptible to position effect variegation including gene silencing^35,36^.

Our finding on *HTT*/*Htt* locus methylation, especially identifying that the most significant HD-associated methylation sites were in close proximity to the repeat expansion mutation itself, may have general implications for understanding the role of epigenetics in repeat expansion disorders. First, similar findings of pathologically relevant abnormal DNAm in the proximity of expanded pathogenic repeats have been observed in other disorders such as fragile X syndrome, myotonic dystrophy type I, and Friedreich’s ataxia^37–39^. Second, a recent study showed 22 disease-associated tandem repeats, including those found within genes: *FMR1* (gene mutated in fragile X), *frataxin*, *HTT*, ataxin 1, and ataxin 3 were located in chromatin domain boundaries enriched with an ultra-high density of CpG islands and bound by methylation-sensitive chromatin factor *CTCF*^40^.

Another important area of investigation is whether methylation changes on *HTT* exon 1 have any impact on the somatic instability of the mutant CAG repeat itself. Recent large-scale human genetic studies revealed variants in mismatch repair enzymes (e.g., *MLH1*, *MSH2, MSH3*), which are known to modify the germline and somatic instability of expanded *HTT* CAG repeats in KI mouse models^41^, act as modifiers of the age of motor symptom onset in HD^5^.

Our EWAS of HDGEC status in human blood identified 33 loci at a genome-wide significance level. Besides *HTT* itself, the EWAS revealed the following genes known to modify mutant *HTT* aggregation or toxicities. DNAJB8 is a member of the DNAJ (Hsp40) chaperone family and inhibits polyglutamine mutant *HTT* aggregation and amyloid fibril formation^42,43^. HDAC4 binds to mutant *HTT* in a polyglutamine length-dependent manner and is localized to the cytoplasmic inclusions in striatal neurons in HD mouse models, and genetic reduction of murine *Hdac4* reduces cytoplasmic mutant *Htt* aggregation and restores cortico-striatal synaptic transmission in HdhQ150 KI mice^44^.

Another clinically relevant finding determined by blood EWAS of HD motor progression was that epigenetic modifiers were linked to symptom progression in HD. Our analysis focused on progression of motor symptoms in manifest HD cases discovered three CpGs: cg26919387 near *PEX14* (*p* = 9.3 × 10^−9^), cg12823408 near *GRIK4* (*p* = 3.0 × 10^−8^), and cg21497164 in *COX4I2* (*p* = 6.5 × 10^−8^). These findings were not confounded by medications used for treating movement disorders (tetrabenazine). Arguably, the most interesting gene identified was *GRIK4*, which is selectively expressed in the cortex and striatum. Studies of mice deficient in *Grik4* or elevated *Grik4* gene dosage have demonstrated the critical role of this molecule in sensory motor gating and protection against excitatory neurotoxicity^45^.

While our EWAS findings in blood were different to those observed in a previous analysis of brain tissue using a limited brain methylation dataset^6^, we found that several brain regions exhibited hypermethylation of the *HTT* cg22982173 locus in HD cases. In our two independent murine studies, we observed genome-wide significant hyper- and hypomethylation at the *Htt* locus in the striatum and cerebellum of Q175 KI mice. Furthermore, our top EWAS hit inside the *HTT* locus replicated in human lymphoblastoid cells and in fibroblasts.

Overall, our study provides new insights and directions of HD research in the areas of accelerated epigenetic aging, the role of *HTT* locus methylation and HD biology, and other methylation sites that may act as epigenetic modifiers that modulate HD progression.

## Methods

### Data sets

HD mutation carriers did not differ significantly from controls in terms of chronological age. However, pre-manifest HD samples tended to be significantly younger than manifest HD samples. Further, the study sample ascertainment induced a strong negative correlation between CAG length and age at enrollment (*r* = −0.5, *p* = 2.4 × 10^−40^ in Enroll-HD). Thus, manifest HD status (or equivalently pre-manifest HD status) was confounded by chronological age in our data. Unfortunately, we observed a strong association between HD disease status and Illumina chip ID in the Enroll-HD study (but not in the Registry-HD study). Most Illumina chips contained 12 manifest HD samples or 12 pre-manifest HD samples or 12 controls. Since chip effects probably confound the relationship between HD status and DNAm levels, we removed the chip effects from the DNAm data by averaging the DNAm levels of each CpG across the 12 samples on a given chip. The HD status of a chip corresponded to the shared HD status of the 12 samples. By replacing individual level data by “aggregated Enroll-HD data,” we effectively removed chip effects at the cost of reduced sample sizes (from *N* = 910 Enroll-HD samples to 76 corresponding chips). Illumina chips were not confounded by HD status in the Registry-HD study.

### Enroll-HD: a prospective registry study in a global HD cohort

We obtained phenotypic data (demographic data and clinical assessments) from the longitudinal Enroll-HD study based on 15,203 individuals. Only a subset of these 15,203 individuals was profiled on the DNAm array: (1) Enroll-HD data 1 were based on a single buffy coat sample from *N* = 910 individuals (controls, pre-manifest HD, and manifest HD) from Enroll-HD data 1. The Enroll-HD data 2 involved two buffy coat samples (from two separate blood draws) from 368 HTT mutation carriers. The first blood sample from each individual was on average collected 7.9 years before the second sample. We removed 11 of the 368 individuals from the analysis because (a) two individuals were severe outliers according to our epigenetic clock analysis and nine individuals already appeared in the Enroll-HD data 1. Thus, we only studied 357 individuals from the Enroll-HD data 2 study in our analysis.

Enroll-HD is a longitudinal, observational, multinational study that will integrate two existing HD registries, Registry-HD in Europe and COHORT in North America and Australia, while also expanding to include sites in Latin America and Asia. ClinicalTrials.gov Identifier: NCT01574053 (Principal Investigator: Bernhard G. Landwehrmeyer and Study Director: Joseph Giuliano).

With indefinite and ongoing annual assessments, the goal of Enroll-HD is to build a large and rich database of longitudinal clinical information and biospecimens. This database will serve as a basis for future studies aimed at developing tools and biomarkers for progression and prognosis, identifying clinically relevant phenotypic characteristics, and establishing clearly defined endpoints for interventional studies. Core datasets are collected annually on all research participants as part of this multi-center longitudinal observational study of HD. Data are monitored for quality and accuracy using a risk-based monitoring approach. All sites are required to obtain and maintain local Ethics Committee approvals.

The primary objective of Enroll-HD is to develop a comprehensive repository of prospective and systematically collected clinical research data (demography, clinical features, family history, genetic characteristics) and biological specimens (blood) from individuals with manifest HD, unaffected individuals known to carry the HD mutation or at risk of carrying the HD mutation, and control research participants (e.g., spouses, siblings, or offspring of HD mutation carriers known not to carry the HD mutation). Enroll-HD is conceived as a broad-based and long-term project to maximize the efficiencies of non-clinical research and participation in clinical research. With over 200 sites in roughly 30 countries, Enroll-HD is the largest database available for HD researchers. Our Enroll-HD data 1 involved blood samples from Northern America (81%), Europe (12.8%), Latin America (1.1%), and Oceania (5.1%). All individuals were 18 years or older. Both sexes were eligible for the study as were healthy volunteers. A non-probability sampling method was used.

Informed consent from the potential participant or legal representative is a pre-requisite for study participation. Participants’ IRB consent is an unconditional pre-requisite for patient participation in the Enroll-HD, approved by the Scientific Review Committee [https://enroll-hd.org/for-committees/]. More details of the ethics oversight are listed in Enroll-HD protocol [https://www.enroll-hd.org/enrollhd_documents/Enroll-HD-Protocol-1.0.pdf].

Below, we describe the study population from Enroll-HD. Patients with HD and their family members are recruited from speciality clinics (Human Genetics, Neurology, Psychiatry) that advise and treat people affected by HD. In addition, in some areas, community clinics and neurologists who see HD patients will recruit participants for this study. More details for the study population including inclusion and exclusion criteria are listed in Supplementary Note 2.

### Registry-HD data

The European Huntington’s Disease Network (EHDN) provided us with whole DNA samples from the Registry-HD core research project. We analyzed 376 samples from whole blood and 100 samples from lymphoblastoid cell lines. Registry-HD is a multi-center, multinational observational study with no experimental treatment. It forms part of the Huntington Project, a worldwide collaboration dedicated to finding treatments that make a difference for HD. Registry-HD is sponsored by the High Q Foundation, a non-profit organization that supports a variety of research projects seeking to find treatments for HD. The study description of Registry-HD is listed in NIH ClinicalTrial database [https://clinicaltrials.gov/ct2/show/NCT01590589]. Participants IRB consent was obtained at the time of the potential participant’s visit an explanation of the study, approved by the Scientific and Bioethical Advisory Committee of the EHDN. Details of the consent are listed the section of “Participant informed consent” of the study protocol [http://www.ehdn.org/wp-content/uploads/2018/06/registry-protocol-2.0.pdf].

### Human fibroblasts

We obtained *N* = 64 fibroblast samples from the CHDI Multiple Tissue Monitoring (MTM) study. The study involved 37 HD mutation carriers and 27 age-matched controls. The mean age was 42.7 years and the proportion of males was 45%. DNAm arrays were profiled in fibroblast tissues. The mean CAG lengths in the HD mutation group were 44 and 19 for the long and short allele, respectively. The CAG lengths were not available in the control group and were set to 28 and 18 for the long and short allele, respectively. The data are described in Table 1.

### DNAm data

DNA was extracted from the Registry-HD and the Enroll-HD samples using the Miller salting out procedure and the QIAGEN QIAamp DNA Midi Kit. DNA was eluted in Buffer AE (a TE buffer). Bisulfite conversion was performed using the Zymo EZ DNA Methylation Kit (Zymo Research, Orange, CA, USA) as well as subsequent hybridization to the Illumina DNAm array, and scanning (iScan, Illumina) were performed according to the manufacturer’s protocols by applying standard settings. DNAm array data were profiled in HumanMethylation450 Bead Chip for Enroll-HD data 1 and Registry-HD, and profiled on the EPIC array (also known as 850k array) for Enroll-HD data 2. DNAm levels (*β* values) were determined by calculating the ratio of intensities between methylated (signal A) and un-methylated (signal B) sites. Specifically, the *β* value was calculated from the intensity of the methylated (M corresponding to signal A) and un-methylated (U corresponding to signal B) sites, as the ratio of fluorescent signals *β* = Max(M,0)/[Max(M,0) + Max(U,0) + 100]. Thus, *β* values range from 0 (completely un-methylated) to 1 (completely methylated). We used the “noob” background normalization method^46^ to account for technical variation in background fluorescence signal. The *noob* approach capitalizes on a new use for the Infinium I design bead types to measure nonspecific fluorescence in the color channel opposite of their design (Cy3/Cy5).

### DNAm age and epigenetic clock

DNAm levels give rise to particularly promising biomarkers of aging since chronological age (i.e., the calendar years that have passed since birth) has a profound effect on DNAm levels in most human tissues and cell types. To study the association of epigenetic AgeAccel with HD, we used (1) Horvath’s pan-tissue DNAm age on the basis of 353 CpGs^14^, (2) Hannum’s DNAm age on the basis of 71 CpGs^15^, (3) DNAmAgeSkinClock on the basis of 391 CpGs^16^, (4) DNAm PhenoAge on the basis of 513 CpGs^17^, and (5) DNAm GrimAge on the basis of 1030 CpGs^18^. The first three DNAm ages were developed as a measure of chronological age, while the last two were developed as a measure of mortality risk. However, all the biomarkers are associated with mortality and morbidity^17,18,47^.

All of the DNAm age models were established through elastic net regression models. For example, the pan-tissue clock is based on methylation levels of 353 dinucleotide markers known as cytosine phosphate guanines or CpGs^14^. The weighted average of these 353 epigenetic markers gives rise to an estimate of tissue age (in units of years), which is referred to as “DNAm age” or as “epigenetic age.” The pan-tissue epigenetic clock method applies to any tissue or cell type that contains DNA (with the exception of sperm), including individual cell types (helper T cells, neurons, glial cells) and complex tissues and organs (blood, brain, bone, breast, kidney, liver, lung)^14^.

To study the association between the DNAm-based biomarkers and HD, we used a measure of epigenetic AgeAccel that has been widely used in our previous studies of aging. We regressed DNAm age on chronological age using a linear regression model and defined AgeAccel as the corresponding raw residuals. By definition, AgeAccel has no linear dependence on chronological age (correlation *r* = 0). Thus, a positive (or negative) value of AgeAccel indicates that the DNAm Age is higher (or lower) than expected based on age. In an analogous manner, we defined the acceleration measures, AgeAccelSkinClock, AgeAccelPheno, and AgeAccelGrim, based on DNAmAgeSkinClock, DNAmAgePheno, and DNAmGrimAge, respectively. We further examined two widely used acceleration measures: IEAA derived from Horvath’s pan-tissue DNAm Age, which is independent of age-related changes in blood cell composition, and extrinsic epigenetic age acceleration based on Hannum’s DNAm Age, which up-weights the contribution of blood cell count measure.

Mathematical details and software tutorials for the epigenetic clock can be found in the Supplements of the relevant publications^14^. An online age calculator can be found at our webpage [https://dnamage.genetics.ucla.edu].

### EWAS of HD disease status

We adjusted the CpG data for sex using a linear model, then related each CpG to HD status using the robust biweight midcorrelation. We used the “estlambda” function in the GenABEL R package to calculate the inflation factors^48^.

We stratified our EWAS analysis by pre-manifest/manifest HD disease status because each stratum lends itself for addressing different biological questions: studying time to HD onset may involve different pathways from those that determine motor progression in manifest HD samples. Because sex can have an effect on DNAm levels, we adjusted the DNAm levels for sex by forming residuals. Since HD germline mutation status was not related to chronological age, there was no need to adjust for chronological age.

Our analysis methods make extensive use of meta-analysis. A simple yet powerful meta-analysis method, known as Stouffer’s method, relies on combining the *Z* statistics from individual datasets (the two blood datasets (aggregated Enroll-HD and the Registry-HD study). Specifically, for each CpG *i* and dataset *a*, one obtains a *Z* statistic *Z*_*ia*_, for example, by the inverse normal transformation of the *p* value. Next, a meta-analysis *Z*_*i*_ statistic for each CpG is calculated as

1 $$Z_i = \frac{1}{{\sqrt {N_{\mathrm{sets}}} }}\;\mathop {\sum }\limits_{a = 1}^{N_{\mathrm{sets}}} Z_{ia}$$

The meta-analysis statistic *Z*_*i*_ is approximately normally distributed with mean 0 and variance 1; the corresponding *p* value is then calculated using the normal distribution.

### Human brain methylation data in multiple regions

We studied the association of HD with brain methylation levels of *HTT* CpG using our previous study data^6^. Postmortem brain samples from HD (*N* = 26) and non-HD (*N* = 39 including 18 Alzheimer’s disease cases and 21 neurologically normal controls) were collected at UCLA and University of Auckland. DNAm arrays were profiled in following brain regions: caudate nucleus (*N* = 29 arrays), cingulate gyrus (*N* = 33), cerebellum (*N* = 42), hippocampus (*N* = 33), parietal cortex (*N* = 64), frontal lobe (*N* = 70), occipital cortex (*N* = 43), temporal cortex (*N* = 37), midbrain (*N* = 26), motor cortex (*N* = 33), sensory cortex (*N* = 33), and visual cortex (*N* = 32). In our association analysis for HD disease status, we removed the samples from left temporal cortex (*N* = 0 for HD), right frontal cortex (*N* = 0 for HD), and right occipital regions (*N* = 2 for HD), yielding a total of 14 brain sub-regions available for our analysis.

### Allelic effects of CAG length alleles on HTT CpG

We studied the joint effect of CAG.long and CAG.short on human *HTT* methylation (at cg22982173) in *N* = 1807 individuals (2164 samples) across five datasets: Enroll data 1 (blood samples), Enroll data 2 (two blood samples for each individual), Registry-HD blood samples, Registry-HD lymphoblastoid cells, and CHDI fibroblast. We tested eight multivariate regression models to evaluate the joint effect of CAG.long, CAG.short, and a quadratic term in CAG.long. The first four multivariate regression models involved the following covariates: (1) additive model with two covariates CAG.long and CAG.short, (2) a single covariate defined as the sum of CAG lengths (=CAG.long + CAG.short), (3) a single covariate defined as the product of CAG lengths (=CAG.long × CAG.short), and (4) a model with three covariates CAG.long, CAG.short, and their interaction term. Polynomial regression models 5–8 evaluated the quadratic effect of CAG.long. Models 5–7 extended models 1–3, respectively, by adding the square term of CAG.long. Model 8 evaluated three covariates square of CAG.long, CAG.short, and their interaction term. Each model also included age at blood draw and sex. These models were evaluated in three subsets: all individuals, only controls, and only HD mutation carriers. We could not evaluate controls in the Registry-HD lymphoblastoid cells and the MTM fibroblast data due to low sample size (*n* < 25 controls).

### CCG-repeat length versus *HTT* methylation

We evaluated five multivariate linear regression models for studying the effect of CCG-repeat length on cg22982173 methylation (dependent variable) in the Registry-HD cohort (up to 372 observations). Model 1 involved two covariates: the two alleles measuring CCG-repeat length. Model 2 extended model 1 by adding two alleles measuring CAG-repeat length. Model 3 is the same as model 1, but limited to the analysis to 209 individuals with homozygosity for CCG_7_ (where 7 equals the CCG-repeat length). Model 4 involved both alleles for CAG length plus HD disease status. Finally, we re-examined model 5 (*N* = 350 observations) that included both alleles of CAG length, but omitted all HD cases that carried an atypical *HTT* structure as defined in ref. ^22^.

### Htt Q175 mouse

In the RRBS study, we analyzed HET Htt KI mouse lines Q175 and Q20 mice^49^. The HET *Htt* KI line expressed one wild-type endogenous *Htt* allele and a second *Htt* allele with KI of human m*HTT* exon 1 with either ~190 CAG repeats (Q175) or 20 CAG repeats (Q20). Male HET mice were crossed with C57BL/6J (B6J.zQ175 KI, JAX stock number: 370476, https://www.jax.org/strain/370476) female mice at the Jackson Laboratory (Bar Harbor, ME)^29^. Animals born within 3–4 days from litters having four to eight pups were identified by ear tags, tail sampled for genotyping, and weaned at ~3 weeks of age. HET mice were selected based on the CAG repeat sizing to allow a Gaussian distribution of CAG repeats in the experimental cohort to avoid skewed distributions. Best Gaussian fit was judged by eye. Body weight cut off: experimental animals had to weigh >11 g (females) and >13 g (males) by 5 weeks of age. Animals presenting any anomaly were excluded. Unacceptable anomalies were cataracts, malocclusion, missing/small eye, ear infection, unreadable, or missing tag. Mice were housed in cages enriched with two play tunnels, a plastic bone and enviro-dri® (Shepherd Specialty Papers). Animal cage changes occurred weekly. The cages were maintained on a 12:12 h light/dark cycle. Water and food were freely available at all times. This study was carried out in strict accordance with the recommendations in the Guide for the Care and Use of Laboratory Animals, NRC (2010). The protocols were approved by the Institutional Animal Care and Use Committee of PsychoGenics, Inc., an AAALAC International accredited institution (Unit #001213).

The mouse methylation array study was conducted at UCLA. HD KI Q175 mice and wild-type littermate control mice were obtained from The Jackson Laboratory (JAX stock number: 370476). Animals were housed in standard mouse cages under conventional laboratory conditions, with constant temperature and humidity, 12 h/12 h light/dark cycle (7.00 a.m./7.00 p.m.) and food and water ad libitum. All animal studies were carried out in strict accordance with National Institutes of Health guidelines and approved by the UCLA Institutional Animal Care and Use Committees.

### Mouse methylation data

For the custom methylation array study (HorvathMammalMethylChip40), we profiled eight tissue samples for each tissue and group defined by disease status (Q175 and Q20). We used the Illumina array for five tissues: cerebellum, striatum, cortex, liver, and blood. In addition, we used another platform RRBS analysis to generated additional methylation data for the cerebellum and striatum (again eight samples per group and tissue). We limited the CpGs with at least 15 observations, yielding 2,896,456 and 2,965,343 CpGs available for the cerebellum and striatum EWAS, respectively. We performed Student’s *t* tests for association tests and combined the results across the tissues based on the Stouffer’s method.

RRBS is a widely used technology because it is cost-effective at measuring cytosine methylation data at hundreds of thousands (or even millions) of locations at a single-nucleotide level. Compared to whole-genome sequencing it is cost effective because it combines restriction enzymes and bisulfite sequencing to enrich for areas of the genome with a high CpG content. Its limitations include a bias towards regions with a high CpG content and relatively low coverage of individual CpGs (compared to an Illumina array).

The mammalian methylation array is attractive because it provides very high coverage (over thousand X) of highly conserved CpGs in mammals, but it focuses on only 37k CpGs that are highly conserved across mammals.

### An ovine transgenic HD model

We analyzed a total of 168 sheep (57% females): 84 HD transgenic sheep age matched with 84 sheep as controls. The age of sheep ranged from 2.9 to 7.0 years with mean ± SD = 4.1 ± 0.8. HD transgenic sheep generated from a new large-animal HD transgenic ovine model^24^. Sheep (*Ovis aries* L.) were selected because the developmental pattern of the ovine basal ganglia and cortex (the regions primarily affected in HD) is similar to the analogous regions of the human brain. Microinjection of a full-length human HTT cDNA containing 73 polyglutamine repeats under the control of the human promotor resulted in six transgenic founders varying in copy number of the transgene^24^.

To isolate genomic DNA, thawed blood samples (300 μl) were treated twice with red cell lysis buffer (300 mM sucrose, 5 mM MgCl_2_, 10 mM Tris pH 8, 1% Triton X-100), for 10 min on ice; each incubation was followed by centrifugation at 1800 r.c.f. for 10 min at 4 °C, and removal of supernatant. Final pellet was incubated in cell digestion buffer (2.4 mM EDTA, 75 mM NaCl, 0.5% sodium dodecyl sulfate) and proteinase K (final concentration 500 μg/ml) for at least 2 h at 50 °C. An equal volume of phenol:chloroform:isoamyl alcohol (25:24:1; pH 8) was added and samples mixed by inversion, followed by centrifugation at 14,000 r.pm. for 5 min at room temperature. This step was repeated if required. Supernatant was collected and mixed with 2× volume 100% ethanol to precipitate DNA. Following removal and evaporation of residual ethanol, genomic DNA samples were resuspended in 50 μl TE buffer (pH 8). Sample concentrations were initially measured on nanodrop to inform further dilution of samples to range of 100–1000 μg for measurement on Qubit. All protocols used were approved by the University of Auckland Animal Ethics Committee (New Zealand) and the SARDI/PIRSA (South Australian Research and Development Institute/Primary Industries and Regions South Australia) Animal Ethics Committee (Approval number 19/02). Moreover, all work involving OVT73 Sheep was approved by PIRSA Animal Ethics Committee with oversite from the University of Auckland Animal Ethics Committee. DNAm arrays were profiled using a custom Illumina methylation array (HorvathMammalMethyl40) based on 38,000 CpG sites in highly conserved regions in mammals. EWAS was performed using the R function “standardScreeningBinaryTrait” from the “WGCNA” R package^50^.

### HD motor progression in the Enroll-HD data 1

To establish a measure of HD motor progression, we used the large-scale Enroll-HD database comprising 14,850 longitudinal observations across 5204 manifest HD individuals. Of the 5204 patients, 312 were initially in pre-manifest then converted to manifest disease status, in which we only used the visits since manifest phase. We applied linear mixed models under R/nlme 3.1–148 with random effects to longitudinal measures of UHDRS total motor scores, adjusted for age, sex, CAG-repeat length, age at motor onset, education attainment, and visit time as fixed effects. The UHDRS total motor score is a sum of 31 items across oculomotor function, dysarthria, chorea, dystonia, gait, and postural stability. Each item is a scale from 0 to 4 indicating no abnormalities (score as 0) to the most severe impairment (score as 4). The possible range of the total motor score is 0–124. We restricted our analysis for the observations with motor scores ≥5. The random effects included a random intercept with respect to individuals and a random slope with respect to visit to account for heterogeneous changes in motor scores across individuals that could not be captured by the fixed effect of visit. Therefore, we computed the empirical Bayes estimates of the random slopes as a “raw” measure of HD motor progression. Next, we regressed the random slopes on sex, age at baseline, CAG-repeat length, age at motor onset and education attainment, and used the residuals as the measure of HD progression for our downstream analysis. The mean (SD) of follow-up estimates were 2.1 (1.03) years in all 5204 manifest individuals and 3.13 (1.27) years in the subset of 354 individuals available with DNAm array data.

### HD progression in Enroll-HD data 2

In an analogous manner, we performed the linear mixed analysis to establish the measure of HD motor progression, using the manifest individuals in the Enroll-HD data 2. The data 2 aimed to profile two longitudinal measures of DNAm array with approximately a 7-year gap; thus, most of the first blood samples were collected during visits by the individuals in the Registry-HD study. We removed the individuals already appearing in the Enroll-HD data and the visits with motor scores <5, leaving 275 manifest individuals (including 73 converting to manifest) remaining in our progression analysis. In establishing the measure of HD progression, there were >1860 observations across the 275 individuals available for our analysis, with a mean follow-up period of ~7.9 years, from the visits aligned with the first DNAm methylation profiles through to the most recent visits. The model framework of the linear mixed analysis was similar to the one used for Enroll-HD data 1, whereas the age at fixed effect was based on the visit aligned with the first methylation profiles. The random slopes with respect to visits were also adjusted for sex, age, CAG-repeat length, age at motor onset, and education attainment for the downstream analysis.

### Registry-HD data

To establish the HD motor progression scores, we used the cross-sectional phase motor scores based on the last visits, similar to what other authors had done for the Registry-HD data^13^. We applied linear regression analysis to the motor scores, adjusted for sex, age at the last visit, age at motor onset, age at DNAm profiles, CAG length and education attainment, and used the residuals as the measure of the progression for downstream analysis.

### Assessment of HD progression in *HTT* cg22982173

We studied the implication of *HTT* cg22982173 in HD progression across different clinical assessment domains. While our primary measure of HD progression was based on motor score assessments across several visits following the blood draw, we also defined analogous measures of progression based on other clinical assessments: functional assessment score, and the Stroop color and word tests.

### Detailed visualization of regional EWAS coMET

We applied the R package comet (version 3.1)^51^ to visualize the genomic regions of interest from our EWAS results.

### EWAS of HD motor progression

We conducted the EWAS of motor progression scores based on the adjusted random slopes on Enroll-HD data 1 and data 2, respectively, and based on the adjusted last motor scores on the Registry-HD data. EWAS was performed with the function “standardScreeningNumericTrait” in the WGCNA R package under R 3.4.3. Effect sizes were based on biweight midcorrelation (bicor) for robust correlation estimates^50^. We combined the results across the three studies via fixed-effect models weighted by inverse variance under METAL (released 2011-03-25)^52^ and only used the overlap CpGs between the Illumina 450k and EPIC array.

### Gaphunter analysis to detect confounding by SNPs

The Gaphunter hunter software allows one to identify CpG probes that are confounded by adjacent SNPs^23^. We applied minfi/Gaphunter (3.6) under R to our blood DNAm data from Enroll-HD data 1, Enroll-HD data 2, and Registry-HD, respectively. The analysis was performed using the R minfi/gapfunction function, which identifies CpG probes with a gap in a (methylation) beta signal greater than or equal to the defined threshold and reports number of clusters constituting the gap. We used the default threshold of 0.05 and a default cut off value of 0.01. Evidence for SNP confounding exists if the CpG exhibits at least two distinct clusters that involve >20 individuals each. Gap patterns were examined in the 33 CpGs associated with HD disease status and the three CpGs associated with motor progression manifest HD cases.

### Functional enrichment analysis

The anRichment software is implemented in the online tool HDinHD^29^ (www.HDinHD.org), which includes published gene sets, including GO (gene ontology) terms^53^, KEGG (Kyoto Encyclopedia of Genes and Genomes) pathways^54^, Molecular Signatures Database gene sets^55^, and curated literature gene sets included in the “userListEnrichment” function^56^ of the WGCNA (3.6.3) R package as well as transcriptomic co-expression network modules identified in analyses of publicly available expression data from HD patients as well as mouse models.

The GOMETH enrichment analysis properly takes into account the different number of probes per gene present on Illumina array (450k in our analysis) by using Wallenius’ noncentral hypergeometric test^30^. We used the GOMETH R function (R version 3.6), which includes GO terms and KEGG pathways.

### Reporting summary

Further information on research design is available in the Nature Research Reporting Summary linked to this article.

## Supplementary information

Supplementary Information

Description of Additional Supplementary Files

Supplementary Data 1

Supplementary Data 2

Supplementary Data 3

Supplementary Data 4

Supplementary Data 5

Reporting Summary

## Data Availability

Our data are available from two data repositories. First, Gene Expression Omnibus (Superseries GSE147004 and subseries GSE146917, GSE147002, GSE147003, and GSE72778 (human brain methylation). Second, from Enroll-HD [https://www.enroll-hd.org/]. Please direct inquiries to info@chdifoundation.org with the words “Enroll-HD Methylation data” in the subject line. All other relevant data supporting the key findings of this study are available within the article and its Supplementary information files or from the corresponding author upon reasonable request. A reporting summary for this Article is available as a Supplementary information file.

## Code availability

This article does not use any custom code or mathematical algorithm. Relevant code or software has been published in previous articles (“Methods”). In addition, software code is available upon request.
