# Supplementary material for: DNA methylation study of Huntington’s disease and motor progression in patients and in animal models
Source: Nat Commun. 2020 Sep 10;11:4529. doi: 10.1038/s41467-020-18255-5 (PMC7484780; doi:10.1038/s41467-020-18255-5)
Supplement: Supplementary file 1 — Supplementary Information [file 41467_2020_18255_MOESM1_ESM.pdf]

Supplementary Material

Lu *et al.*

Contents

Supplementary Material 1

Supplementary Notes 4

Supplementary Note 1: The Unified Huntington's Disease Rating Scale 4

Supplementary Note 2: Study Population from Enroll-HD 4

Supplementary Figures 7

Supplementary Fig. 1 Histograms of UHDRS total motor scores. 7

Supplementary Fig. 2. Epigenetic clock analysis 8

Supplementary Fig. 3 DNA methylation based biomarkers versus chronological age in the first array profiles of Enroll-HD data 2 9

Supplementary Fig. 4 DNA methylation based biomarkers versus chronological age in the second array profiles of Enroll-HD data 2 10

Supplementary Fig. 5 Association of HD with epigenetic clock acceleration measures 11

Supplementary Fig. 6 DNAm age in lymphoblast cells vs DNAm age in blood 12

Supplementary Fig. 7 Association of Huntington disease motor progression with epigenetic age acceleration measures 13

Supplementary Fig. 8 Reproducibility of EWAS results between Enroll-HD and Registry-HD 15

Supplementary Fig. 9 Regional association results of top CpG associated with HD disease status 16

Supplementary Fig. 10 Quantile-quantile plots of EWAS results in blood 17

Supplementary Fig. 11 Association of HD with *HTT* cg22982173 across multiple studies and tissue types 18

Supplementary Fig. 12 Association of HD with *HTT* cg22982173 across brain regions 19

Supplementary Fig. 13 EWAS results in blood vs EWAS results in brain. 20

Supplementary Fig. 14 Detailed analysis of *HTT* cg22982173 versus CAG lengths in non-HD individuals. 21

Supplementary Fig. 15. Detailed analysis of *HTT* cg22982173 versus CAG lengths in HD mutation carriers 22

Supplementary Fig. 16. Histograms of *HTT* cg22982173 in human samples. 23

Supplementary Fig. 17 Non-random association between *HTT* CAG and CCG repeat length. 24

Supplementary Fig. 18 Regional association results of top CpG associated with HD progression 26

Supplementary Fig. 19 Characters of patients with tetrabenazine medications in Enroll-HD data 1 29

Supplementary Fig. 20 Patient characteristics of treated/untreated individuals in Enroll-HD data 2 30

Supplementary Fig. 21 Association of tetrabenazine medications with DNA methylation levels 31

Supplementary Fig. 22 Epigenome-wide association study (EWAS) of tetrabenazine medication 32

Supplementary Fig. 23 Mouse EWAS in *Htt* gene in brain data 1 33

Supplementary Fig. 24 Mouse epigenome-wide association study (EWAS) in brain data 1 34

Supplementary Fig. 25 Mouse studies based on the custom methylation array. 35

Supplementary Tables 37

Supplementary Table 1. DNA Methylation samples mice and sheep 37

Supplementary Table 2. Data used for estimating motor progression in the Enroll-HD study 38

Supplementary Table 3. Characteristics of Enroll-HD data 2 participants with longitudinal DNA methylation profiles 39

Supplementary Table 4. Linear mixed and regression analysis for HD motor progression using Enroll-HD data 2 40

Supplementary Table 5. Linear regression analysis for HD motor progression using Registry-HD data.... 41

Supplementary Table 6. Multivariate linear models of DNAm age in Enroll-HD. 42

Supplementary Table 7. Multivariate linear model of DNAm age in Registry-HD. 43

Supplementary Table 8. Association of Huntington disease motor progression with epigenetic age.......... 44

acceleration measures 44

Supplementary Table 9. Characteristics of aggregated Enroll-HD data 46

Supplementary Table 10 Associations of HTT methylations with CCG repeat length 47

Supplementary Table 11. Meta-analysis EWAS of HD progression 49

Supplementary Table 12. Enrichment analysis results of top 1000 HD related CpGs versus reference gene sets 50

Supplementary Table 13. Enrichment analysis results of top 1000 HD related CpGs versus reference gene sets 52

Supplementary Table 14. Intersection between top 1000 hypomethylated genes in HD and genes that were found from a protein-protein interaction network analysis of polyglutamine disorders 54

Supplementary Table 15. GOMETH Enrichment analysis results of top 1000 HD related CpGs versus reference gene sets. 55

Supplementary Table 16. Enrichment analysis results of top 500 HD progression related CpGs versus reference gene sets. 56

Supplementary Table 17. GOMETH Enrichment analysis results of top 500 HD progression related CpGs versus reference gene sets. 59

Supplementary References 61

Supplementary Notes

Supplementary Note 1: The Unified Huntington's Disease Rating Scale

The Unified Huntington's Disease Rating Scale (UHDRS) was developed as a clinical rating scale to assess four domains of clinical performance and capacity in HD: motor function, cognitive function, behavioral abnormalities, and functional capacity, developed by Huntington Study Group (HSG) [1]. The HSG has extensively assessed the internal consistency and the intercorrelations of the four domains and examined changes in ratings over time. The UHDRS is used to quantify the severity of disease in Huntington patients for clinical assessment. The UHDRS total motor score is a sum of 31 items across oculomotor function, dysarthria, chorea, dystonia, gait, and postural stability. Each item is on a scale from 0 to 4 where 0 indicates no abnormalities while 4 indicates the most severe impairment. The total motor score ranges from zero to 124.

Supplementary Note 2: Study Population from Enroll-HD

Patients with HD and their family members are recruited from specialty clinics (Human Genetics, Neurology, Psychiatry) that advise and treat people affected by HD. In addition, in some areas community clinics and neurologists who see HD patients will recruit participants for this study. Participants may also receive information about the study through a website, clinical practices, support groups, advocacy newsletters, etc. and place a direct request to be considered for participation in the study. Community controls are identified, using advertisements, flyers and newsletters, by study site staff with the support of the Enroll-HD operational staff.

Inclusion Criteria:

Carriers: This group comprises the primary study population and consists of individuals who carry the HD gene expansion mutation.

Controls: This group comprises the comparator study population and consists of individuals who do not carry the HD expansion mutation.

These two major categories can be further subdivided into six different subgroups of eligible individuals:

- Manifest/Motor-manifest HD: Carriers with clinical features that are regarded in the opinion of the investigator as diagnostic of HD.
- Pre-Manifest/-Motor-manifest HD: Carriers without clinical features regarded as diagnostic of HD.

Genotype Unknown: This group includes a first or second degree relative, i.e., related by blood to a carrier, who has not undergone predictive testing for HD and therefore has an undetermined carrier status.

Genotype Negative: This group includes a first or second degree relative, i.e., related by blood to a carrier, who has undergone predictive testing for HD and is known not to carry the HD expansion mutation.

Research Genotyping

Ten ml of peripheral blood were collected in a yellow topped acid citrate dextrose solution (ACD) tube and shipped by a fast courier service to the central biorepository facility. The central biorepository facility processed blood for DNA extraction. Routine quality control studies were conducted to estimate the quality and integrity of the DNA. CAG repeat sizing was carried out using two sets of primer pairs[2]. DNA genotyping was performed in a research lab and therefore, the results should be interpreted as experimental data. To ensure high data quality, an independent Data Safety Monitoring Committee (DSMC) monitored the CAG testing procedure and compared the results to locally available for individual participants. Individual results were not reported to the local sites or to the participants More details can be found in Enroll-HD protocol, https://www.enroll-hd.org/enrollhd_documents/Enroll-HD-Protocol-1.0.pdf.

Family Control: Family members or individuals not related by blood to carriers (e.g., spouses, partners, caregivers).

Community Controls: Individuals unrelated to HD carriers who did not grow up in a family affected by HD. Data collected from community controls are used for generation of normative data for sub-studies.

Participant status is captured in the study database using 2 variables: 1) Investigator Determined Status: this will be based on clinical signs and symptoms and genotyping performed as part of medical care, and is updated at every visit and 2) Research Genotyping Status: this is based on genotyping conducted as part of Enroll-HD study

procedures. Based on research genotyping, participants are reclassified under this variable from Genotype Unknown to 'Carriers' or 'Controls'. Investigators and participants are blinded to this reclassification.

Exclusion Criteria: Individuals with chorea movement disorders in the context of a negative test for the HD gene mutation. For Community Controls: those individuals with a major central nervous system disorder are also excluded (e.g. stroke, Parkinson disease, Multiple Sclerosis, etc.).

Supplementary Figures

Supplementary Fig. 1 Histograms of UHDRS total motor scores.

We present the histograms of Unified Huntington's Disease Rating Scale (UHDRS) total motor scores using Enroll-HD data1 (N=5035), Enroll-HD data2 (N=265), and Registry-HD (N=287), respectively. We only used the individuals with HTT mutations $[(CAG)_{n>36}]$ and restricted our analysis to observations with motor scores ≥ 5. Panels A, C, & E display the histograms of motor scores at baseline; the visit aligned with first DNA methylation profiles, and the visit aligned with DNAm methylation for the three datasets, respectively. Panels B, D & F display the histograms of motor scores at the last visits for the three datasets, respectively. Sample size, mean and standard deviation (SD) are displayed on top of each plot.

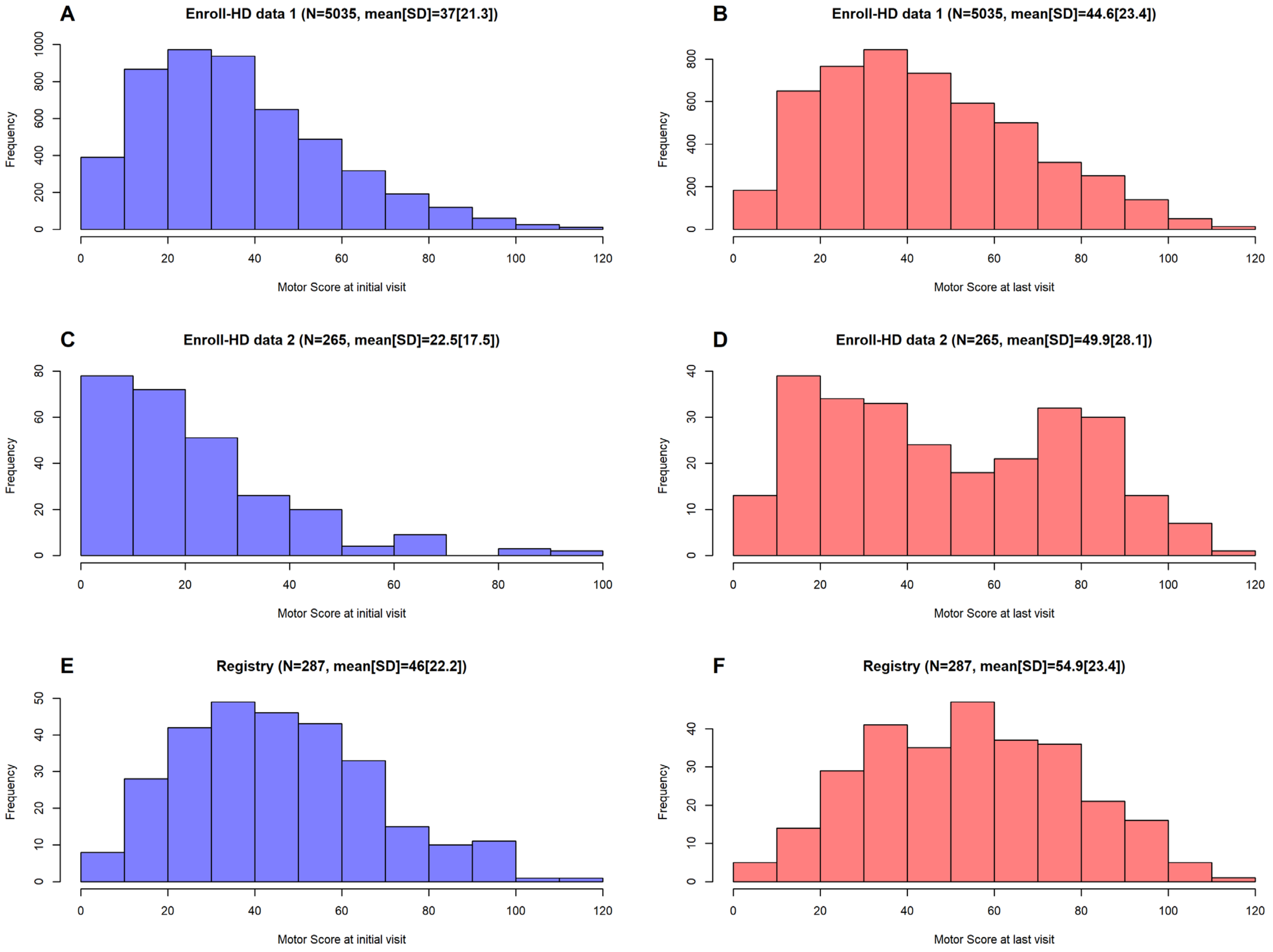

Supplementary Fig. 2. Epigenetic clock analysis

The figure depicts the scatter plots of DNA methylation (DNAm) based biomarkers versus chronological age, using 1) the Enroll-HD data 1 and 2) the Registry-HD data, respectively. We list (A-B) Horvath's DNAm Age [3], (C-D) Hannum's DNAm age [4], (E-F) Horvath's DNAmAgeSkinClock [5], and (G-H) Levine's DNAmPhenoAge [6]. Each plot displays the Pearson correlation coefficient and corresponding unadjusted p-value.

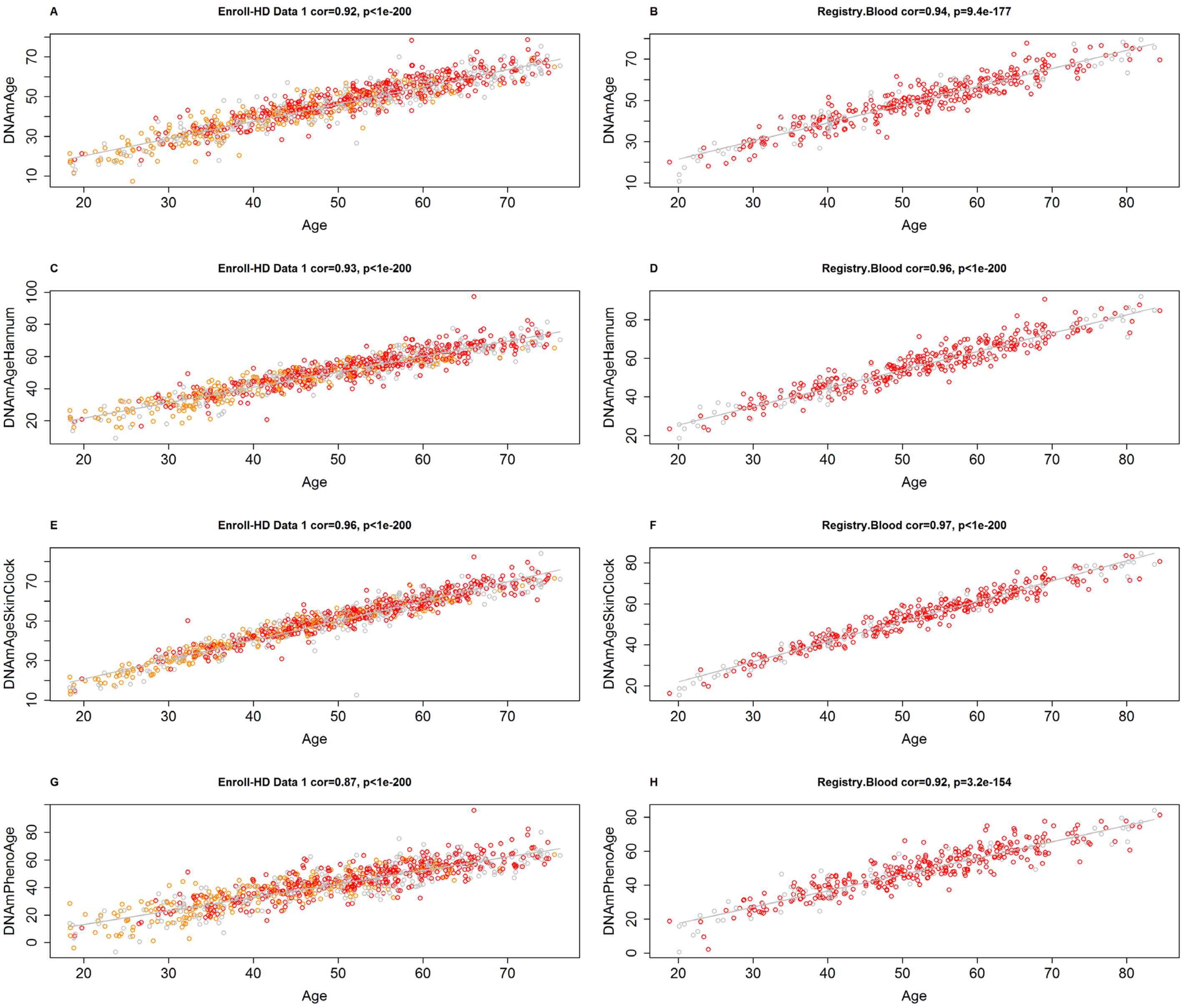

Supplementary Fig. 3 DNA methylation based biomarkers versus chronological age in the first array profiles of Enroll-HD data 2

The figure depicts the scatter plots of DNA methylation (DNAm) based biomarkers versus chronological age, using the first array profiles from the Enroll-HD data 2. The samples (points) are colored by disease status: red for manifest HD (N=204) and orange for pre-manifest (N=153). We list (A) Horvath's DNAm Age [3], (B) Hannum's DNAm age [4], (C) Horvath's DNAmAgeSkinClock [5], and (D) Levine's DNAmPhenoAge [6]. Each plot displays the Pearson correlation coefficient and corresponding unadjusted p-value.

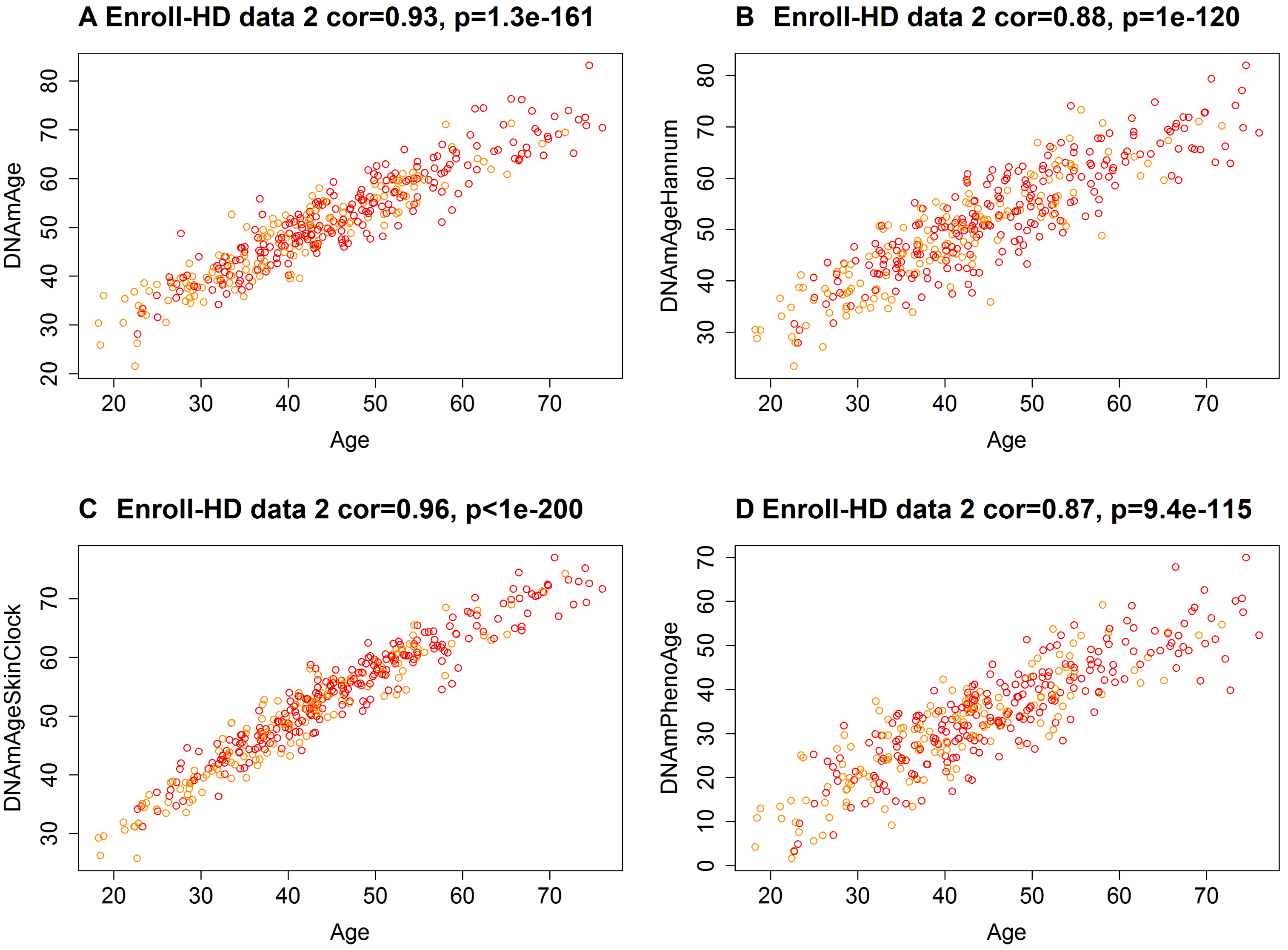

Supplementary Fig. 4 DNA methylation based biomarkers versus chronological age in the second array profiles of Enroll-HD data 2

The figure depicts the scatter plots of DNA methylation (DNAm) based biomarkers versus chronological age, using the second array profiles from the Enroll-HD data 2. The samples (points) are colored by disease status: red for manifest HD (N=279) and orange for pre-manifest (N=77). We list (A) Horvath's DNAm Age [3], (B) Hannum's DNAm age [4], (C) Horvath's DNAmAgeSkinClock [5], and (D) Levine's DNAmPhenoAge [6]. Each plot displays the Pearson correlation coefficient and corresponding unadjusted p-value.

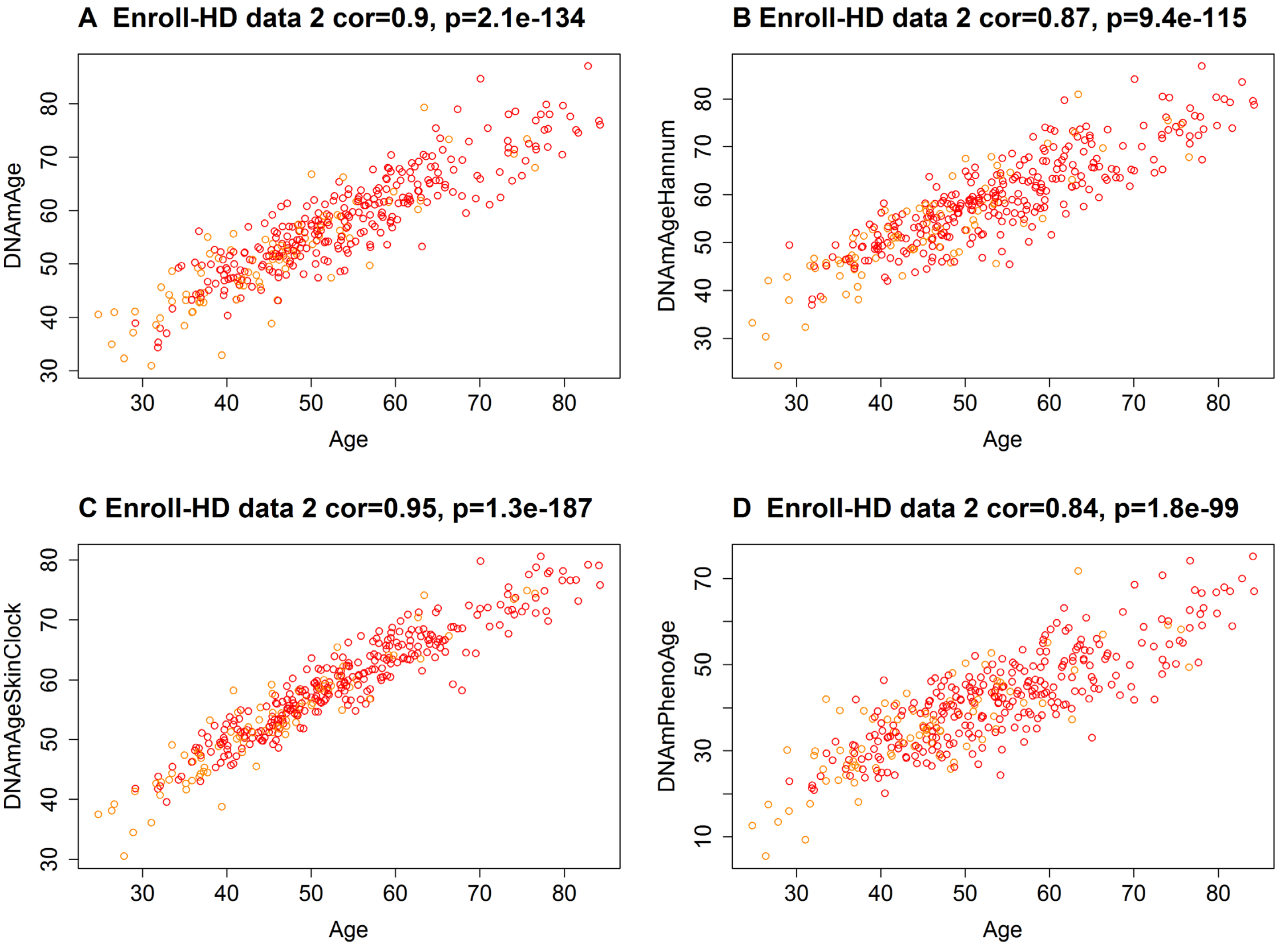

Supplementary Fig. 5 Association of HD with epigenetic clock acceleration measures

The figure depicts the bar plots for the associations of HD disease status with DNA methylation (DNAm) based biomarkers using 1) the Enroll-HD data 1 (N=910)and 2) the Registry-HD data (N=376), respectively. Epigenetic measures were adjusted for age including (A-B) age adjusted DNAm age based on Horvath (AgeAccelerationResidual [AgeAccel]) [3], (C-D) intrinsic epigenetic age acceleration (IEAA) derived on the basis of Horvath's DNAm age, (E-F) extrinsic epigenetic age acceleration derived on the basis Hannum's DNAm age [4], (G-H) age adjusted DNAmAgeSkinClock (AgeAccelSkinClock) [5], (I-J) age-adjusted DNAm PhenoAge (AgeAccelPheno) [6], and (K-L) age-adjusted DNAm GrimAge [7] (AgeAccelGrim). The bar plots report the p-value of a non-parametric group comparison test (Kruskal-Wallis). The y-axis of the bar plots depicts the mean and one standard error. Group sizes (i.e. number of individuals per disease category) can be found as small grey numbers underneath each bar.

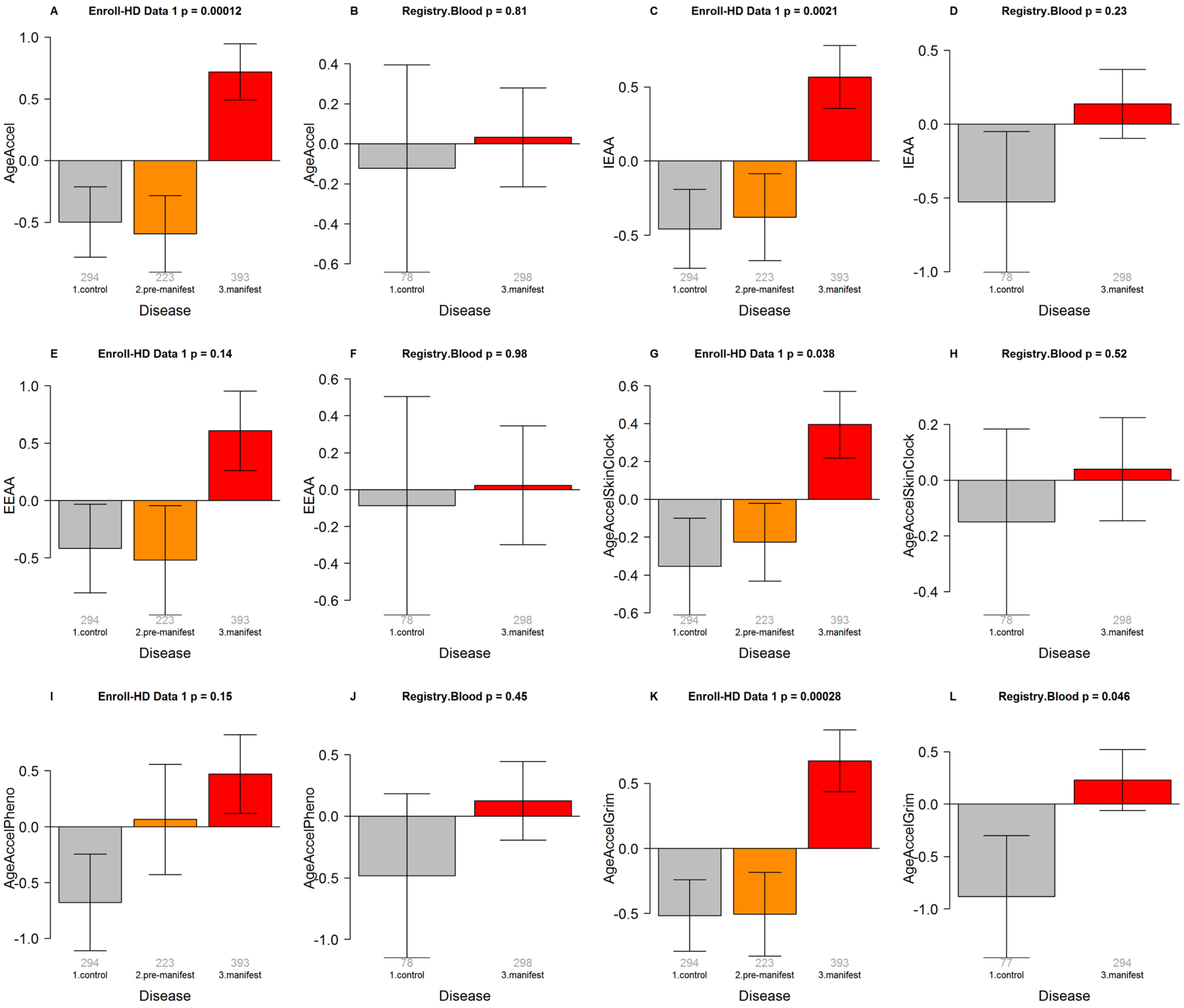

Supplementary Fig. 6 DNAm age in lymphoblast cells vs DNAm age in blood

For n=100 participants of the Registry-HD study, we had both blood samples and corresponding lymphoblastoid samples. A) Scatter plot of DNAm age of lymphoblastoid cells vs DNAm age of the corresponding blood sample is shown. Samples (dots) are colored by HD status (red=HD mutation carrier, black=control). B) This box plot depicts the difference between DNAmAge in blood and DNAm age in lymphoblastoid cell lines. The notch around the horizontal line depicts the 95% confidence interval around the median difference. The difference is significantly different from zero (mean difference= 3.32, 95 % confidence interval [1.28, 5.37], Student T test for paired samples, two-sided p-value = 0.0017). C) DNAm age in lymphoblastoid cells versus chronological age in these 100 samples. D) DNAm age in blood versus chronological age in these 100 samples. Panels A, B and D display Pearson correlation coefficients and corresponding nominal (unadjusted) two sided p values.

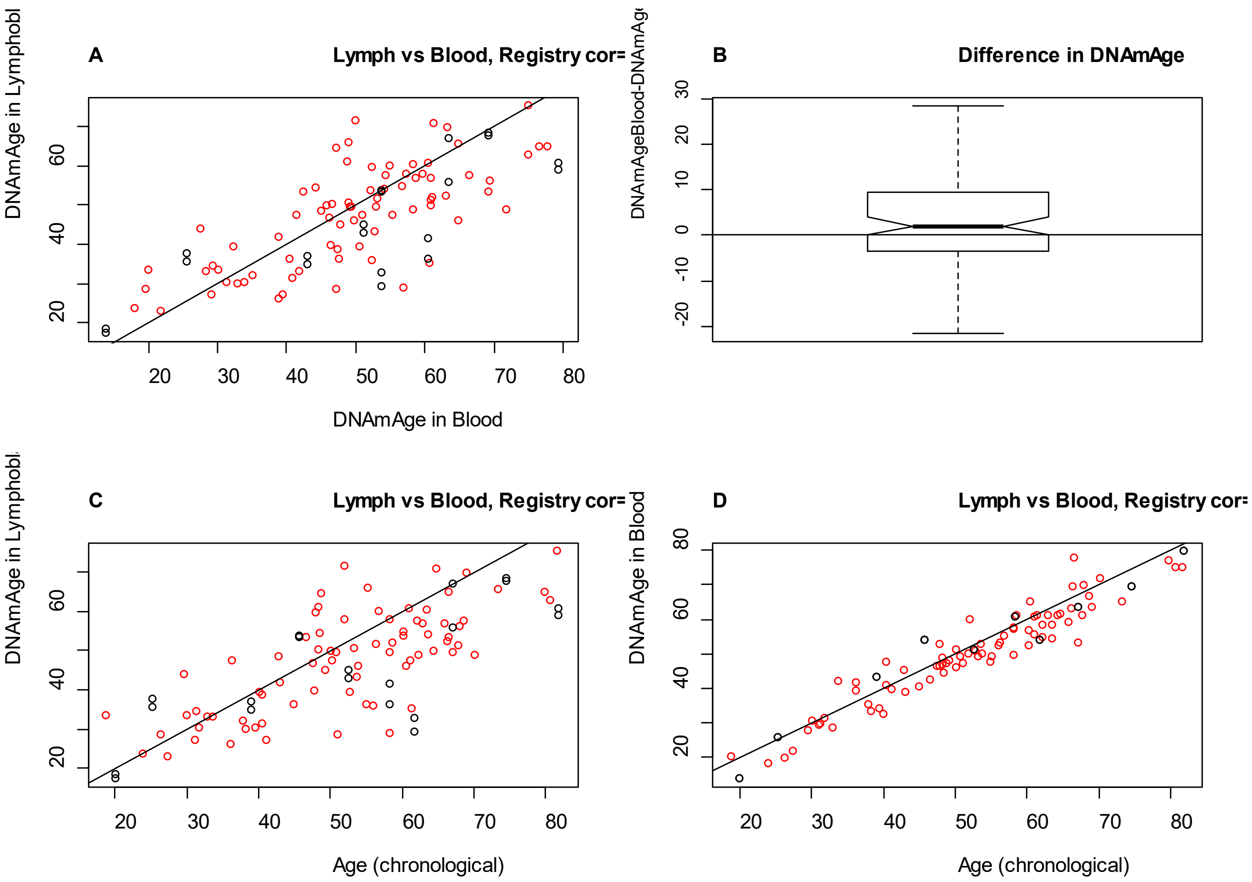

Supplementary Fig. 7 Association of Huntington disease motor progression with epigenetic age acceleration measures

We present the bar plots to depict the associations of adjusted motor progression with age-adjusted epigenetic measures. The analysis was conducted in a total of 917 HD manifest patients from A) Enroll HD data 1 (N=354) associated with short term follow-up (median ~ 3 years, B) Enroll HD data 2 (N=275) associated with longer term follow-up (median ~8 years), and C) Registry cohort (N=288). The panel D lists the meta-analysis results combining across the three studies (panels A-C) by fixed effect models weighted by inverse variance. In Enroll HD, motor progression measures were based on the random slopes in linear mixed model analysis, adjusted for age, CAG length, age onset of HD disease, and educational attainment. In Registry, motor progression measures were based on the motor score evaluated in the last visit adjusted for age, CAG length, age onset of HD disease and educational attainment. Epigenetic measures were adjusted for age including 1) age adjusted DNAm age based on Horvath (AgeAccelerationResidual [AAR]) [3], 2) intrinsic epigenetic age acceleration (IEAA) derived on the basis of Horvath's DNAm age, 3) extrinsic epigenetic age acceleration derived on the basis Hannum's DNAm age [4], 4) age adjusted DNAmAgeSkinClock (AgeAccelSkinClock [AgeAccelSkin]) [5], 5) age-adjusted DNAm PhenoAge (AgeAccelPheno) [6], and 6) age-adjusted DNAm GrimAge [7] (AgeAccelGrim). The height of each bar corresponds to the statistical significance level of an association test between the motor progression and the age-adjusted epigenetic biomarker. More precisely, the y-axis displays minus logarithm (base 10) transformed p-values. The titles of the panels report robust (biweight midcorrelation) coefficients and corresponding nominal (unadjusted) two sided correlation test p-values.

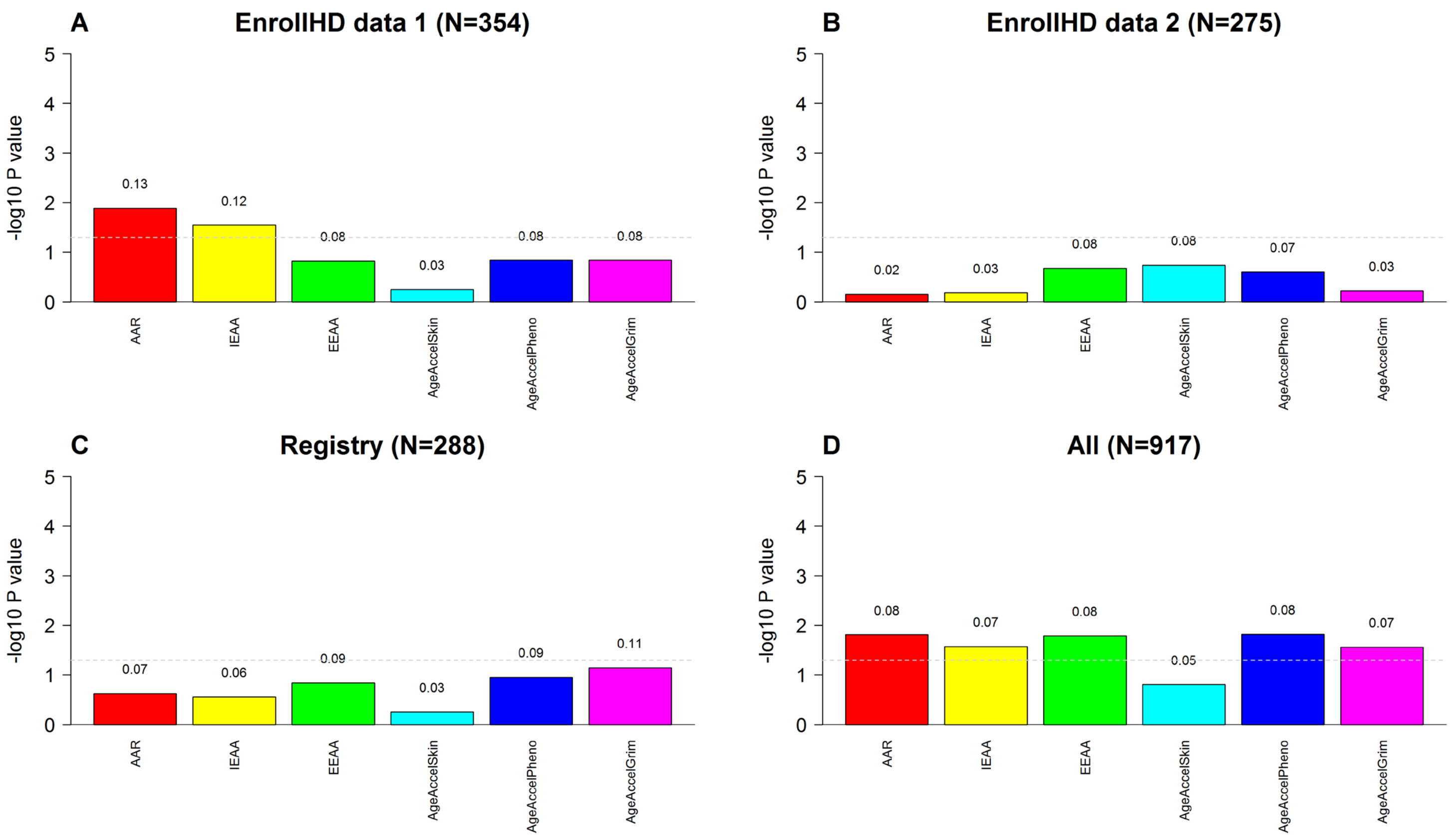

A
EnrollHD data 1 (N=354)
B
EnrollHD data 2 (N=275)
C
Registry (N=288)
D
All (N=917)
-log10 P value
0
1
2
3
4
5
AAR
IEAA
EEAA
AgeAccelSkin
AgeAccelPheno
AgeAccelGrim
0.13
0.12
0.08
0.03
0.08
0.08
0.02
0.03
0.08
0.08
0.07
0.03
0.07
0.06
0.09
0.03
0.09
0.11
0.08
0.07
0.08
0.05
0.08
0.07

Supplementary Fig. 8 Reproducibility of EWAS results between Enroll-HD and Registry-HD

Each axis reports a Z statistic for correlating DNA methylation data with HD mutation status. The y-axis reports the results in the aggregated Enroll-HD study. The x axis reports the results in the Registry-HD data. Each dot corresponds to a CpG. A positive value indicates that the CpG tends to be hypermethylated in HD mutation carriers. The Pearson correlation coefficient and corresponding nominal (unadjusted) two sided correlation test p-value can be found in the title.

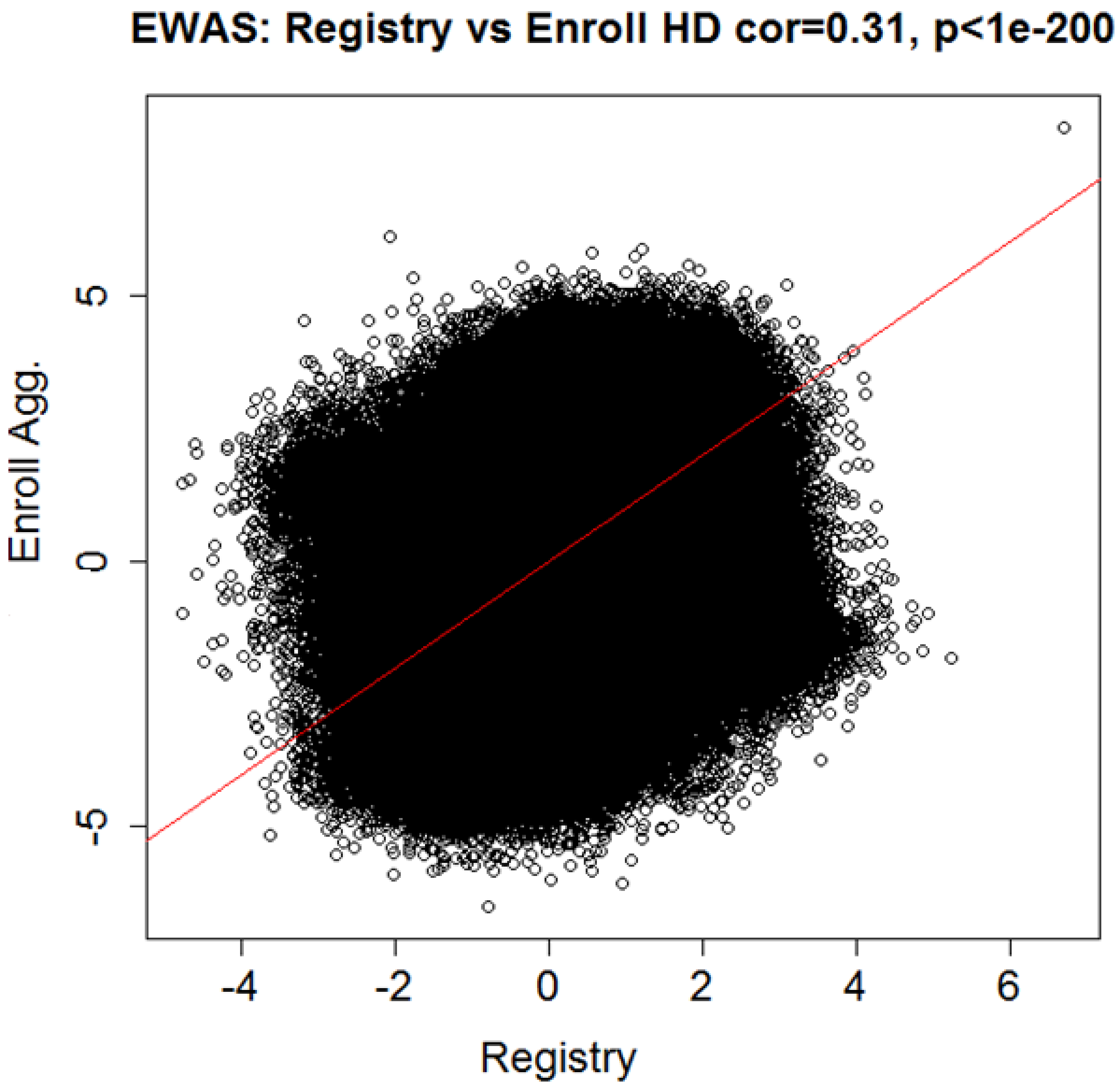

Supplementary Fig. 9 Regional association results of top CpG associated with HD disease status

The plot depicts the association results surrounding the leading CpG cg22982173 in the *HTT* gene. We used the R package coMET [9] to display Genomic annotations including ENCODE data, gene tracks, reference CpG-sites, and the correlation patterns of CpG methylation levels among our Enroll-HD data1 individuals.

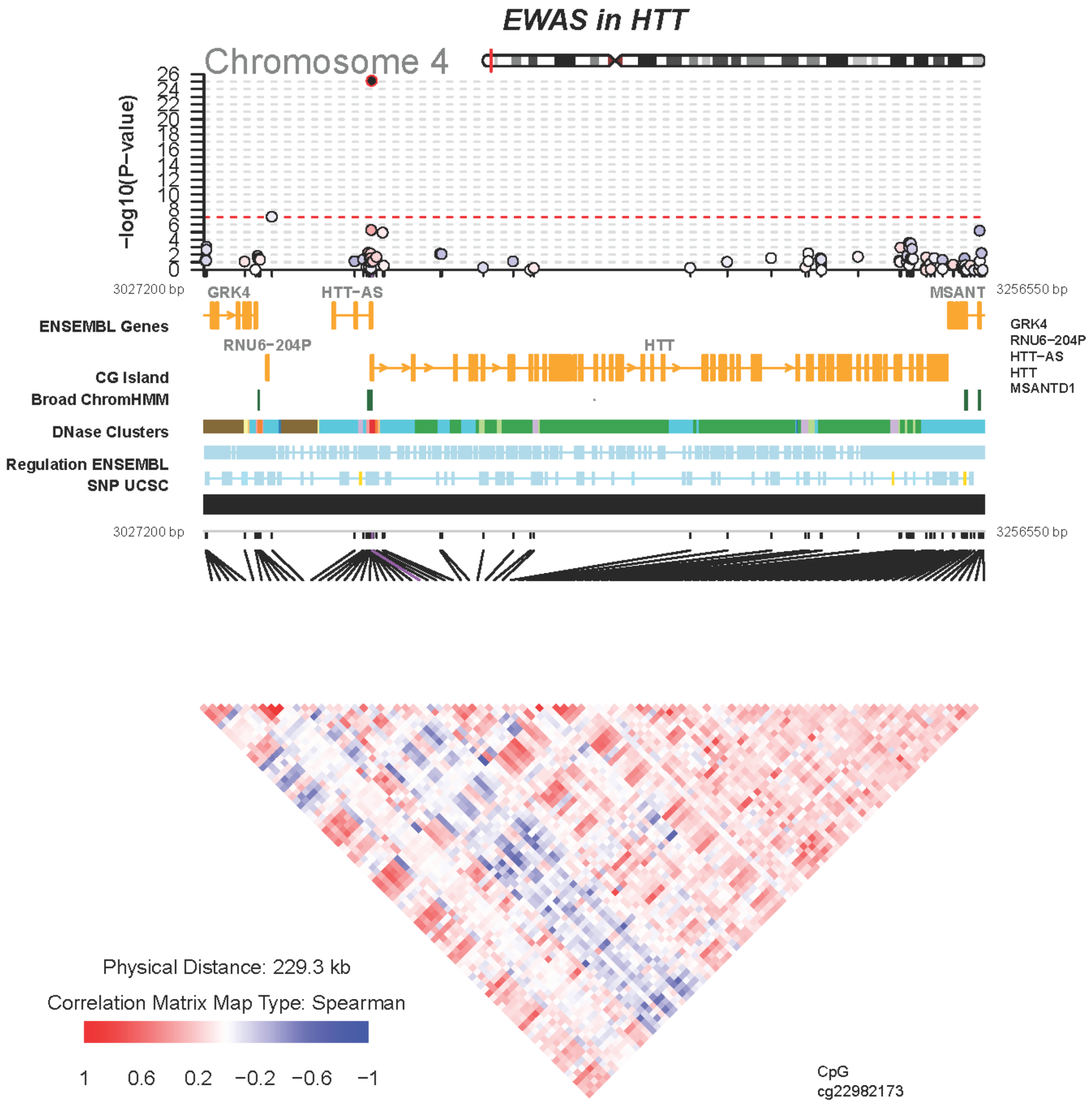

Supplementary Fig. 10 Quantile-quantile plots of EWAS results in blood

The QQ plots reveal significant genomic inflation in our EWAS studies (estimated by the lambda value). Expected chi-square statistics (y-axis) versus expected chi-square statistics (x-axis). A) Results for the meta-analysis statistics that combined aggregated Enroll-HD and Registry-HD data. B) Results for the aggregated Enroll-HD data only, C) Results for the Registry-HD data only.

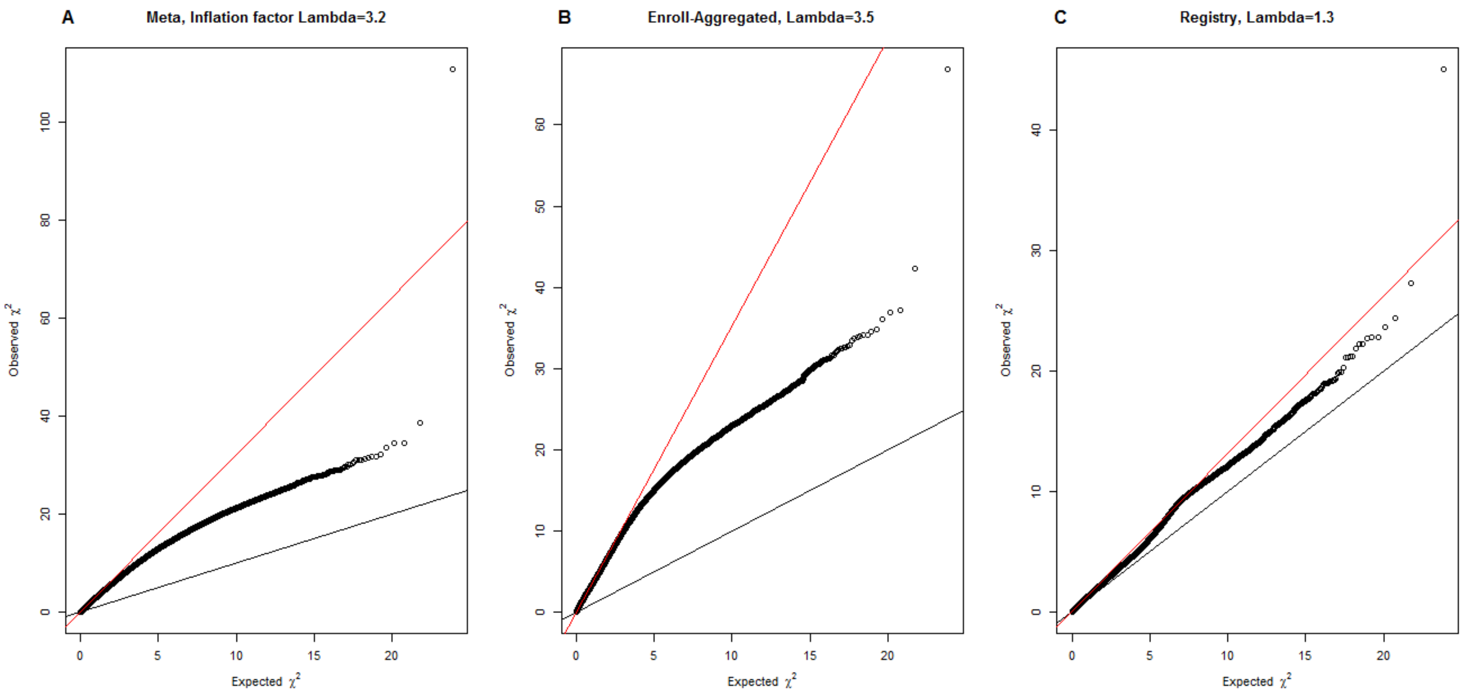

Supplementary Fig. 11 Association of HD with *HTT* cg22982173 across multiple studies and tissue types

These bar plots depict the associations of HD disease status with HTT cg22982173 methylation in A) the Enroll-HD data 1 blood samples (N=910), B) the Registry-HD blood samples (N=376), C) the Registry-HD lymphoblastoid cells (n=100), and D) CHDI fibroblast tissues, respectively (N=64). The bar plots report Kruskal-Wallis test two sided nominal p-values. The y-axis of the bar plots depicts the mean and one standard error. Group sizes (i.e. number of individuals per disease category) can be found as small grey numbers underneath each bar.

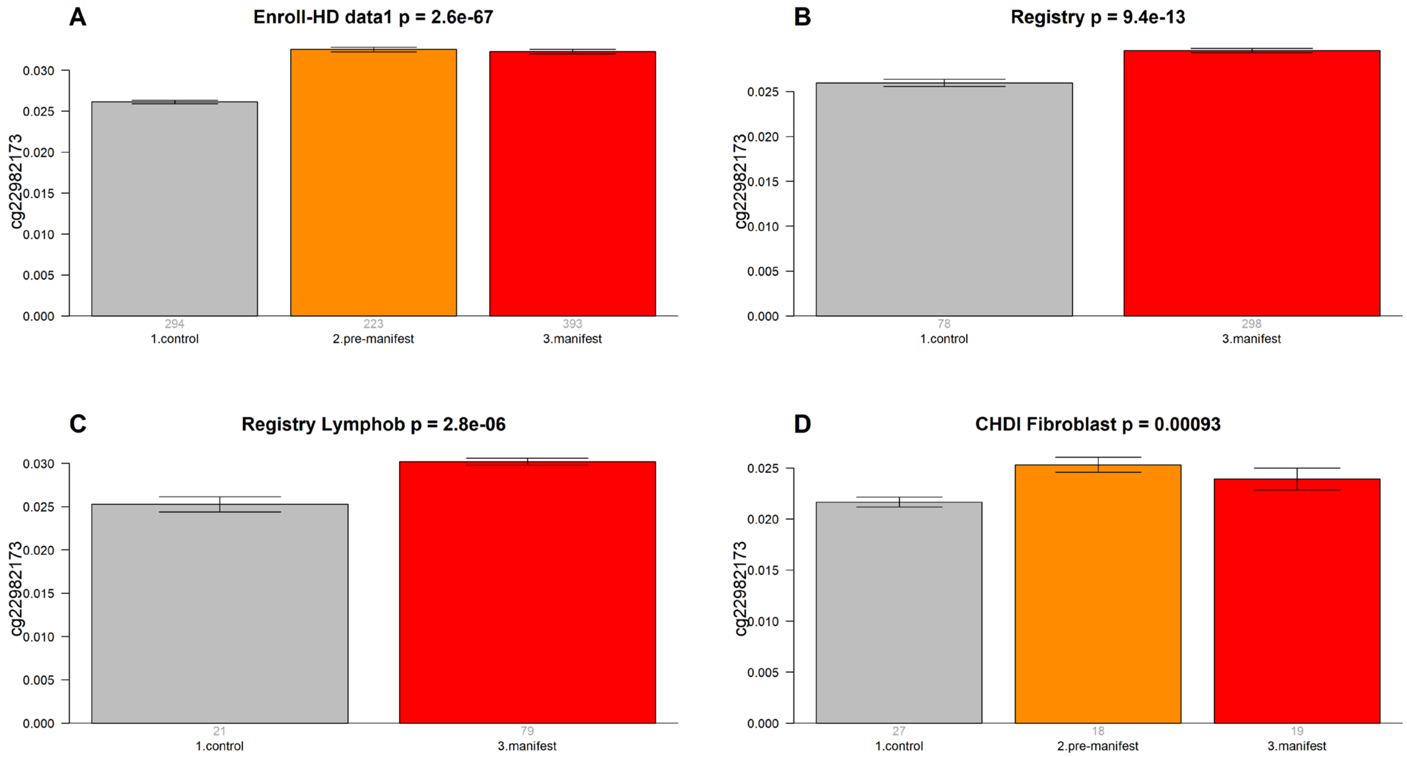

Supplementary Fig. 12 Association of HD with *HTT* cg22982173 across brain regions

The bar plots depict the associations between HD disease status and *HTT* cg22982173 methylation using brain methylation data from our previous study (26 HD versus 39 not HD individuals) [10]. These data were generated using 475 samples from 17 different brain regions. We removed three brain regions from the analysis due to limited sample sizes in the HD group ($n \leq 2$). The bar plots display a total of 14 brain regions and report Kruskal-Wallis nominal two sided p-values. The blue dots correspond to individual observations that underlie the respective mean values. The y-axis of the bar plots depicts the mean of one standard error. The numbers of individuals per group are reported as grey numbers in each bar.

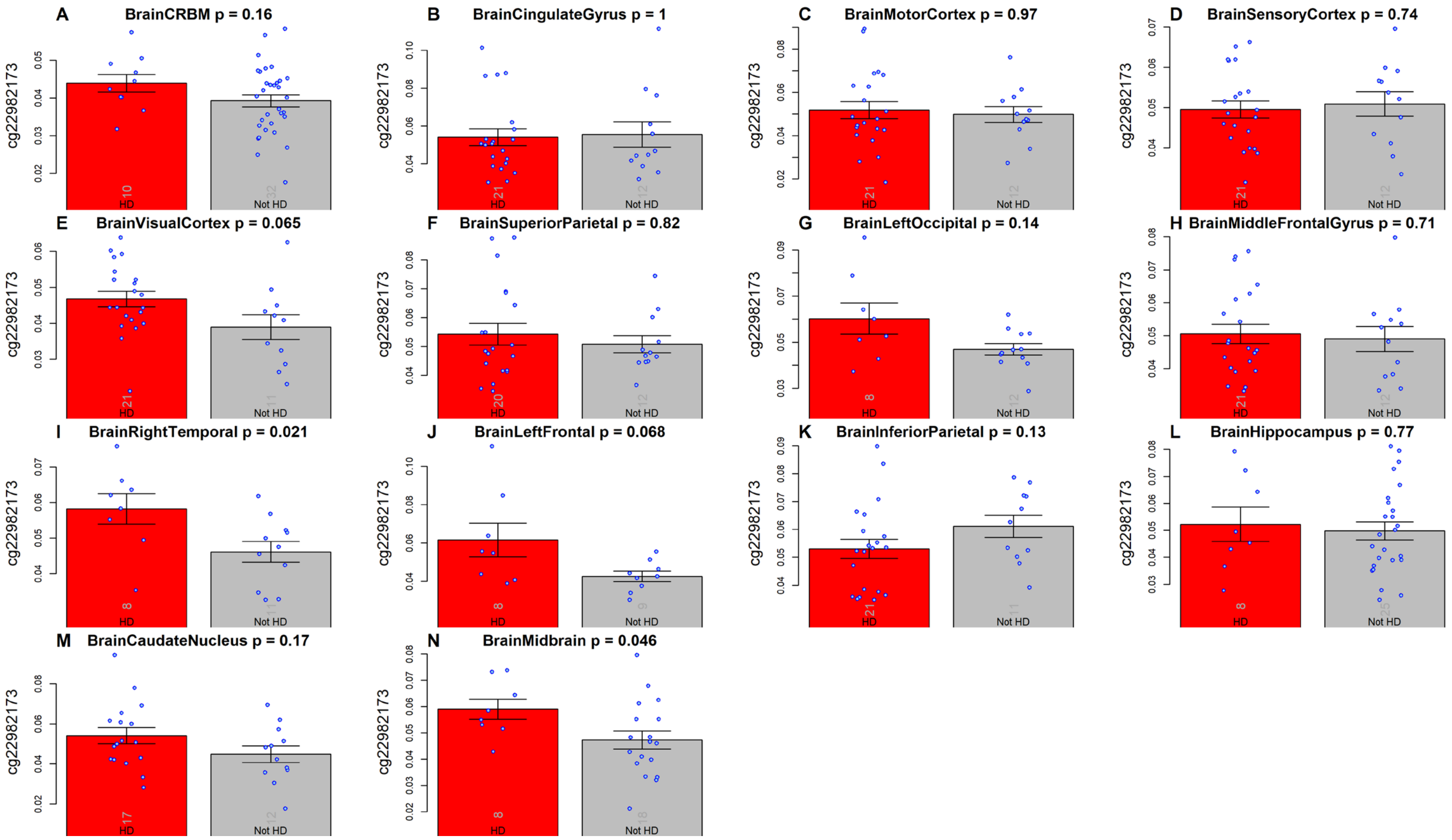

Supplementary Fig. 13 EWAS results in blood vs EWAS results in brain.

Each axis reports a Z statistic for relating individual CpGs to HD status. Thus, under the null hypothesis of no association with HD status, each Z statistic follows a standard normal distribution. A positive (negative) value for the Z statistic indicates that the CpG is hypermethylated (hypomethylated) in HD cases. The x-axis depicts the meta-analysis Z statistic resulting from our EWAS analysis of HD in blood (meta-analysis Z statistic based on aggregated Enroll-HD and Registry-HD data). The y-axis of each plot reports Z statistics for relating HD status to brain methylation data from A) all 3 lobes combined, B) frontal lobe only, C) occipital lobe only, and D) parietal lobe only. The Pearson correlation coefficient and corresponding (unadjusted) two sided p-value can be found in the title of each panel. The y-axis of panel A reports Stouffer's meta-analysis Z statistic across the 3 lobes, i.e. Z.meta=(Z.Frontal+Z.Occipital+Z.Parietal)/sqrt(3). This analysis has a major limitation: the EWAS results from the different brain regions are based on a small sample size (multiple brain regions from only 26 HD cases and 39 controls from [11]). It would be important to revisit this analysis with EWAS findings based on hundreds of postmortem brain samples. Note that our most significant CpG in blood (inside *HTT*, right most dot) is not significantly associated with HD status (absolute Z statistic is less than 2).

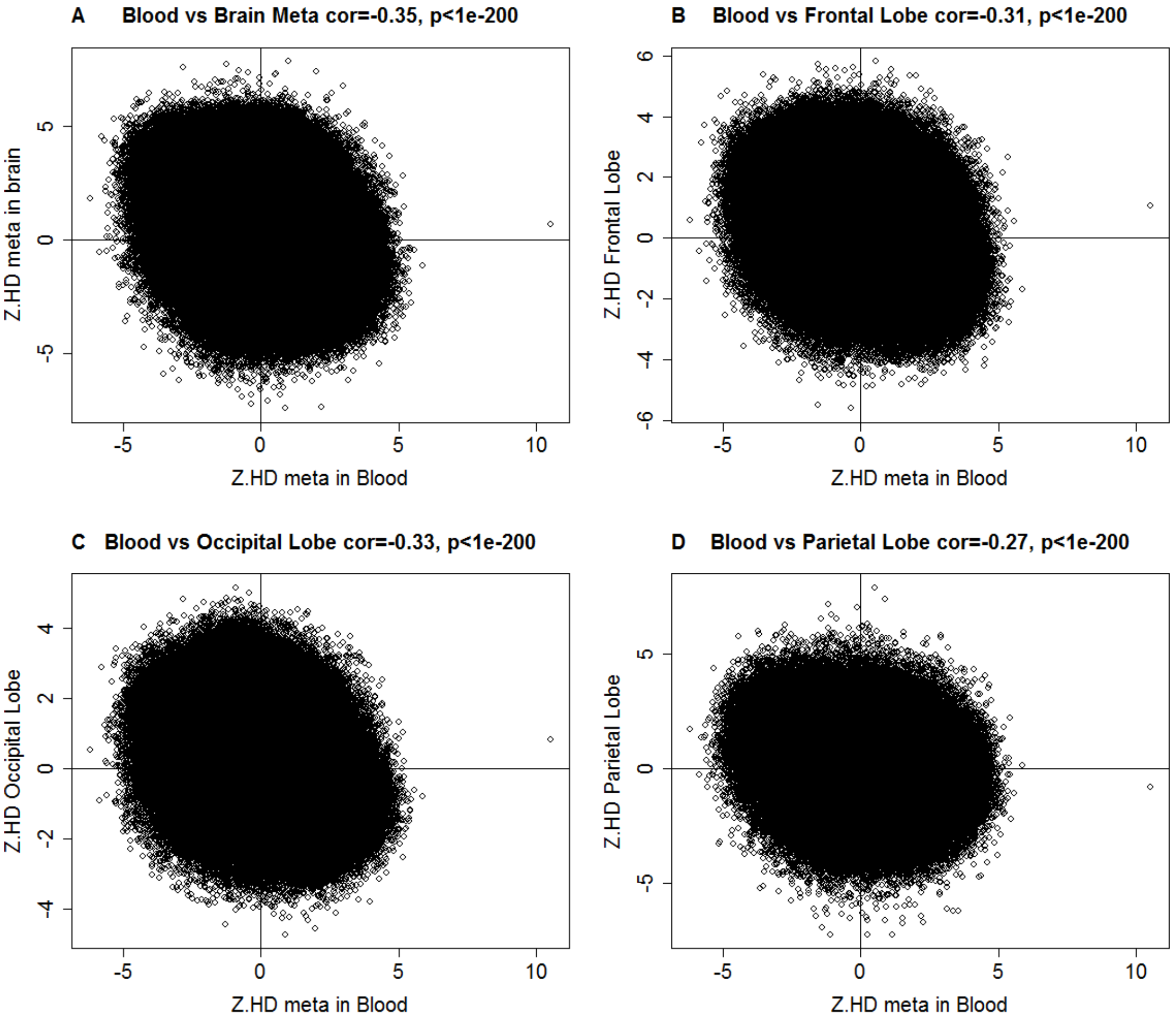

Supplementary Fig. 14 Detailed analysis of *HTT* cg22982173 versus CAG lengths in non-HD individuals. The scatter plots show the product of CAG expansion lengths on two alleles (x-axis) versus DNA methylation levels of *HTT* cg22982173 across A) Enroll-HD data1 blood, B) Registry-HD blood, and C) Registry-HD lymphoblastoid, respectively. To combine the results across studies, we applied linear regression analysis of cg22982173 on the product of CAG lengths and used the corresponding beta values as effect sizes in our meta-analysis. Fixed effect model weighted by inverse variance indicates a very significant p-value of 4.5×10^{-8}. The plots report the corresponding Pearson correlation coefficient and nominal (unadjusted) p-value.

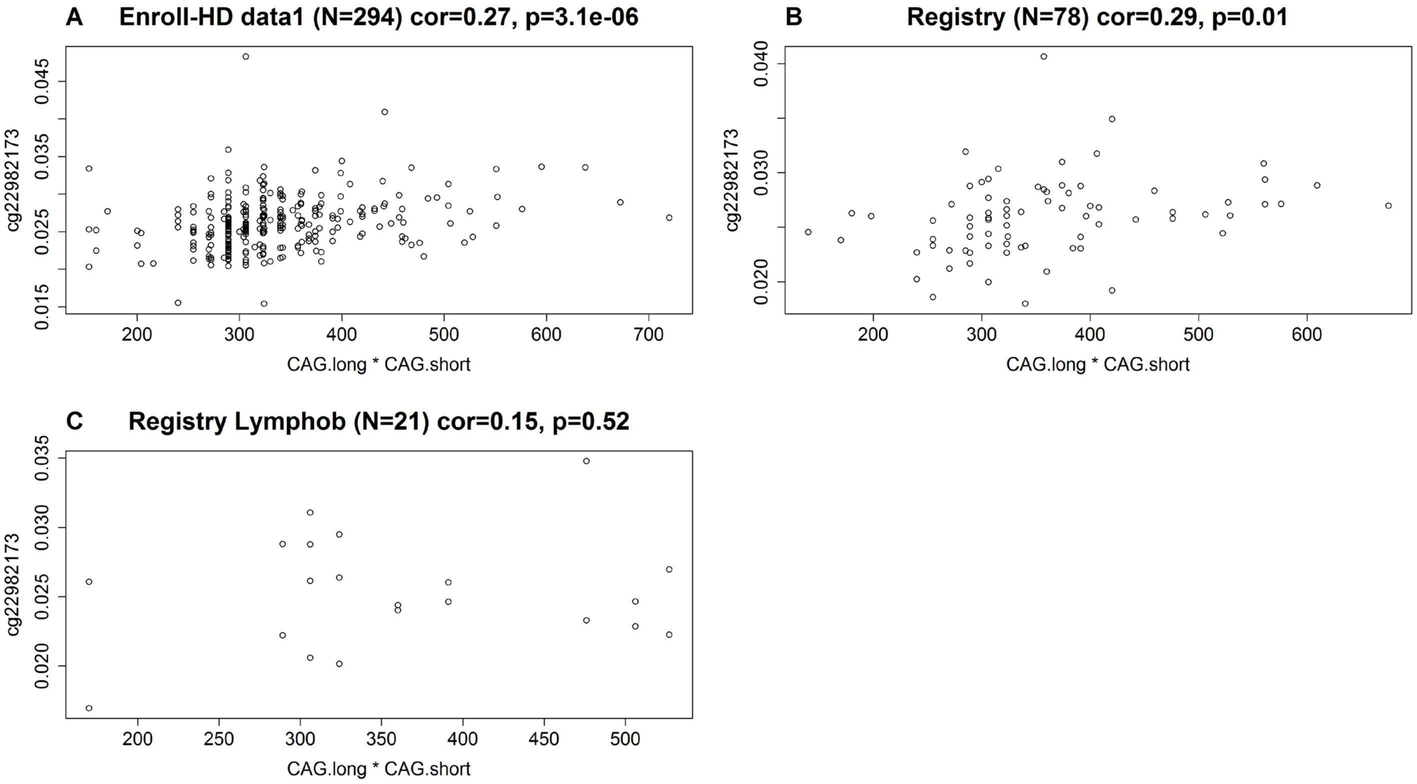

Supplementary Fig. 15. Detailed analysis of *HTT* cg22982173 versus CAG lengths in HD mutation carriers.

The scatter plots depict the product of CAG expansion lengths of two alleles (x-axis) versus DNA methylation levels of *HTT* cg22982173 using the HD mutants across A) Enroll-HD data1 blood, B) Enroll-HD data 2 blood, C) Registry-HD blood, D) Registry-HD lymphoblastoid, and E) CHDI MTM fibroblast samples, respectively. To combine the results across studies, we applied linear (mixed) regression analysis of cg22982173 on the product of CAG lengths and used the beta values as effect sizes in our meta-analysis. Fixed effect model weighted by inverse variance indicated a striking p-value of $3.9\text{x}10^{-27}$. Points in the scatter plots are colored according to HD status: red=manifest HD, orange=pre-manifest HD, black=control. The plots report the corresponding Pearson correlation coefficient and nominal (unadjusted) two sided p-value.

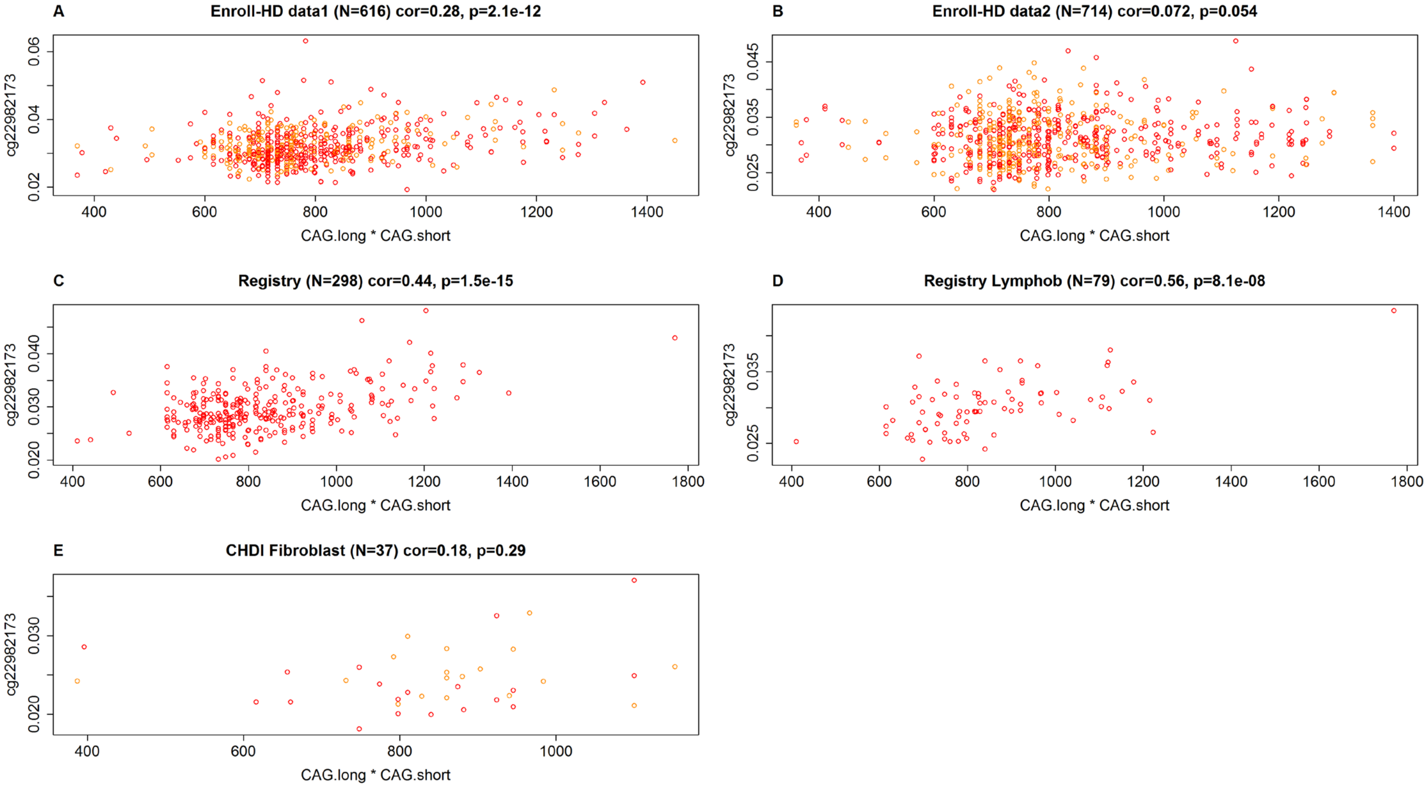

Supplementary Fig. 16. Histograms of *HTT* cg22982173 in human samples.

We present the histograms of cg22982173 (*HTT*) gene stratified by disease group and datasets: A-C, Enroll-HD data1 blood (total N=910); D-E Registry-HD blood (total N=714 longitudinal samples from 357 individuals); F-G Registry-HD blood (total N=376); H-I Registry-HD lymphoblastoid (total N=100); J-L CHDI fibroblast samples (total N=64), respectively. Histograms are colored according to HD status: red=manifest HD, orange=pre-manifest HD, grey=control. The title of each panel reports the sample size, mean value, and standard deviation (SD) of cg22982173. The color represents HD status: red=manifest HD, orange=pre-manifest HD, grey=control without HD CAG expansion.

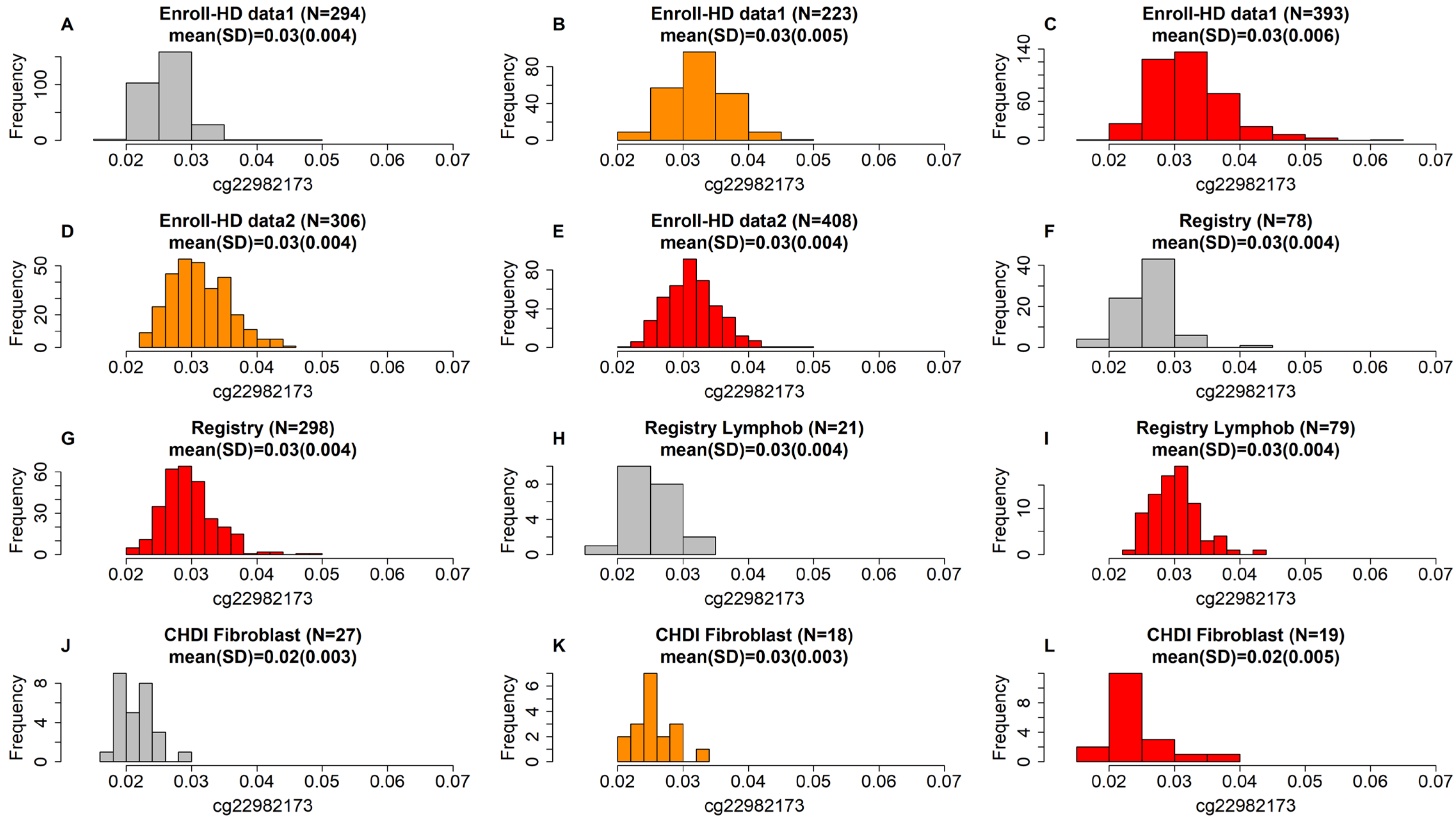

Supplementary Fig. 17 Non-random association between *HTT* CAG and CCG repeat length.

This figure shows the association analysis between CCG repeats and CAG repeats using 372 individuals from the Registry-HD dataset. Panel (A) depicts a scatterplot showing the relationship between *HTT* CAG and CCG repeat length. The line of best fit is shown ($r^2 = 0.16$, $p < 2 \times 10^{-16}$, $n = 744$ alleles). Panel (B) depicts histograms showing the number of alleles observed with each CCG repeat length on either non-disease associated (green) or HD mutant chromosome (CAG ≥ 36) (red) in the Registry-HD cohort ($n = 297$).
Panel A indicates a marked association between CAG and CCG repeat length in the Registry-HD cohort ($r^2 = 0.16$, p<2 E-16). Moreover, panel B shows that the distribution of CCG alleles is markedly shifted between HD and non-HD chromosomes (P= 0.0005). This effect is primarily driven by the over-representation of the most common CCG allele (CCG_7, where 7 denotes length of CCG repeat) on mutant expanded HD chromosomes. Conversely, the second most common CCG allele (CCG_{10}) is highly underrepresented on mutant expanded HD chromosomes. Additionally, it has recently been determined that individuals with rare atypical *HTT* repeat haplotypes (on which the intermediary sequence between the pure CAG and CCG repeats is altered) can have unusually late or early onset HD, relative to the total number of polyglutamines encoded by the CAA and CAG repeats inherited [12-14].

(A)

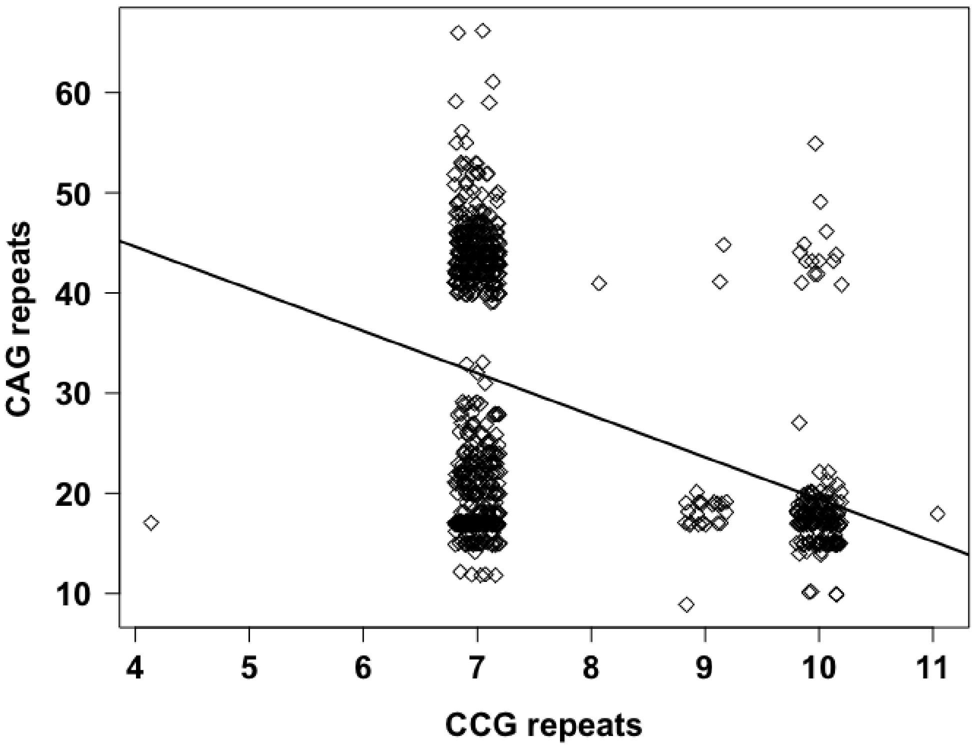
CAG repeats
60
50
40
30
20
10
4
5
6
7
8
9
10
11
CCG repeats

(B)

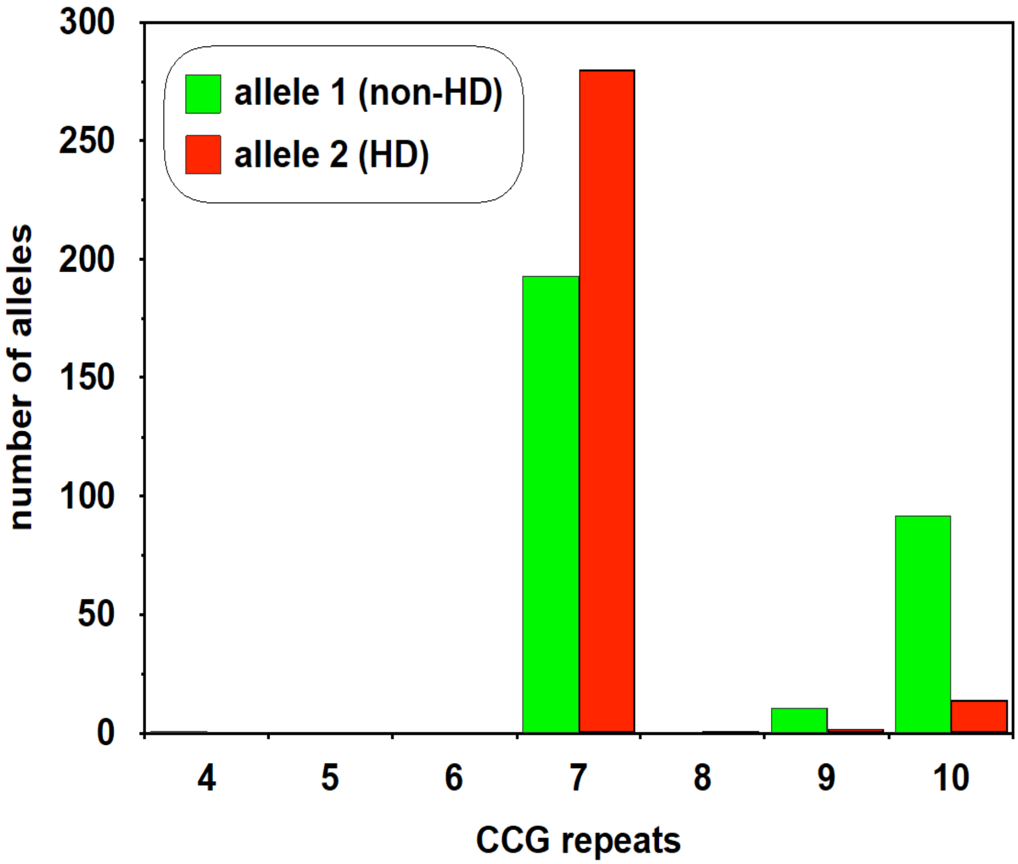
allele 1 (non-HD)
allele 2 (HD)
number of alleles
300
250
200
150
100
50
0
4
5
6
7
8
9
10
CCG repeats

Supplementary Fig. 18 Regional association results of top CpG associated with HD progression

The plot depicts EWAS of HD progression near A) cg26919387, B) cg12823408 and C) cg21497164, respectively. All of the three CpGs were associated with P<1.0E-7 We used the R package coMET [9] to display genomic annotations including ENCODE data, gene tracks, reference CpG-sites, and the correlation patterns of CpGs methylation levels among our Enroll-HD data 1 individuals.

(A)

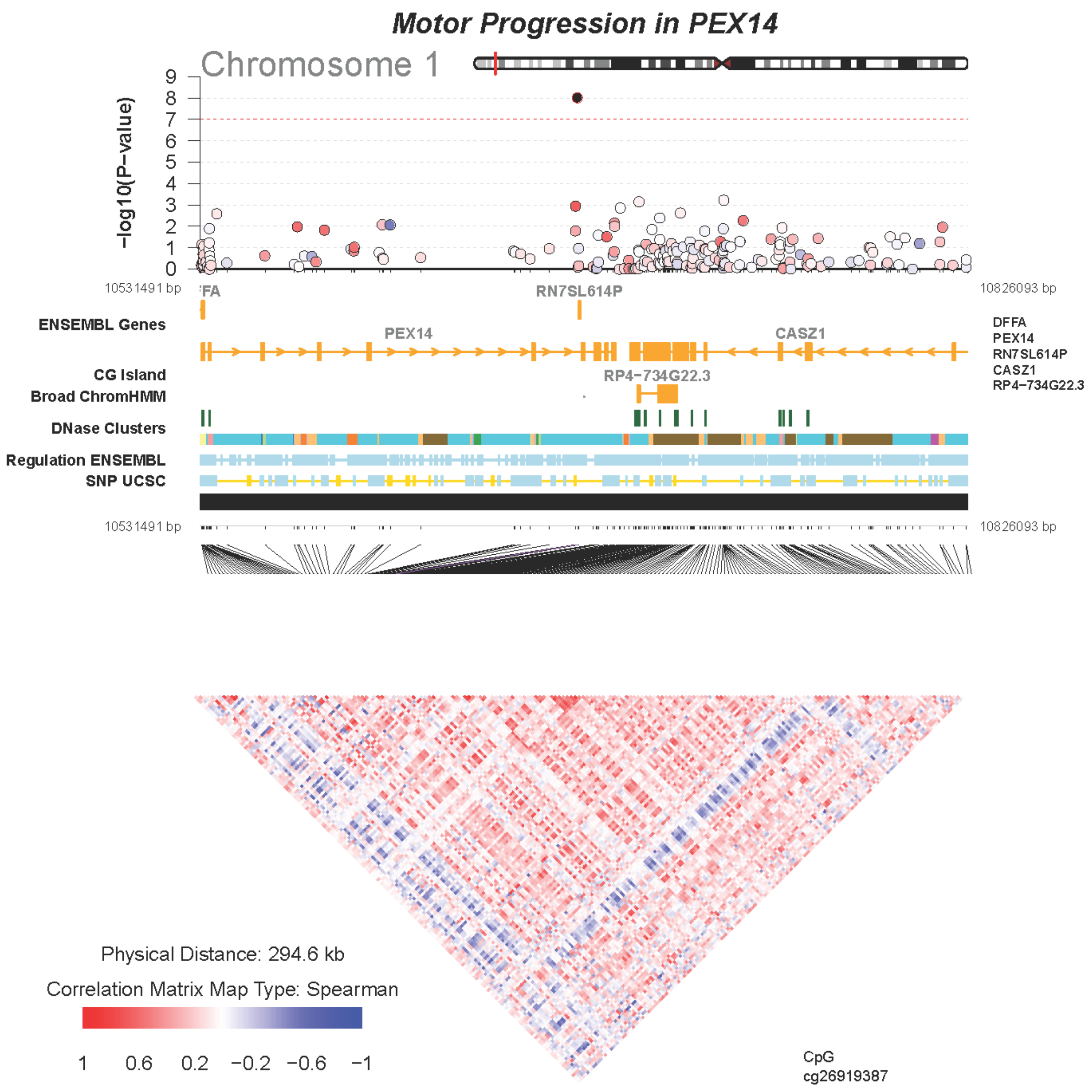

(B)

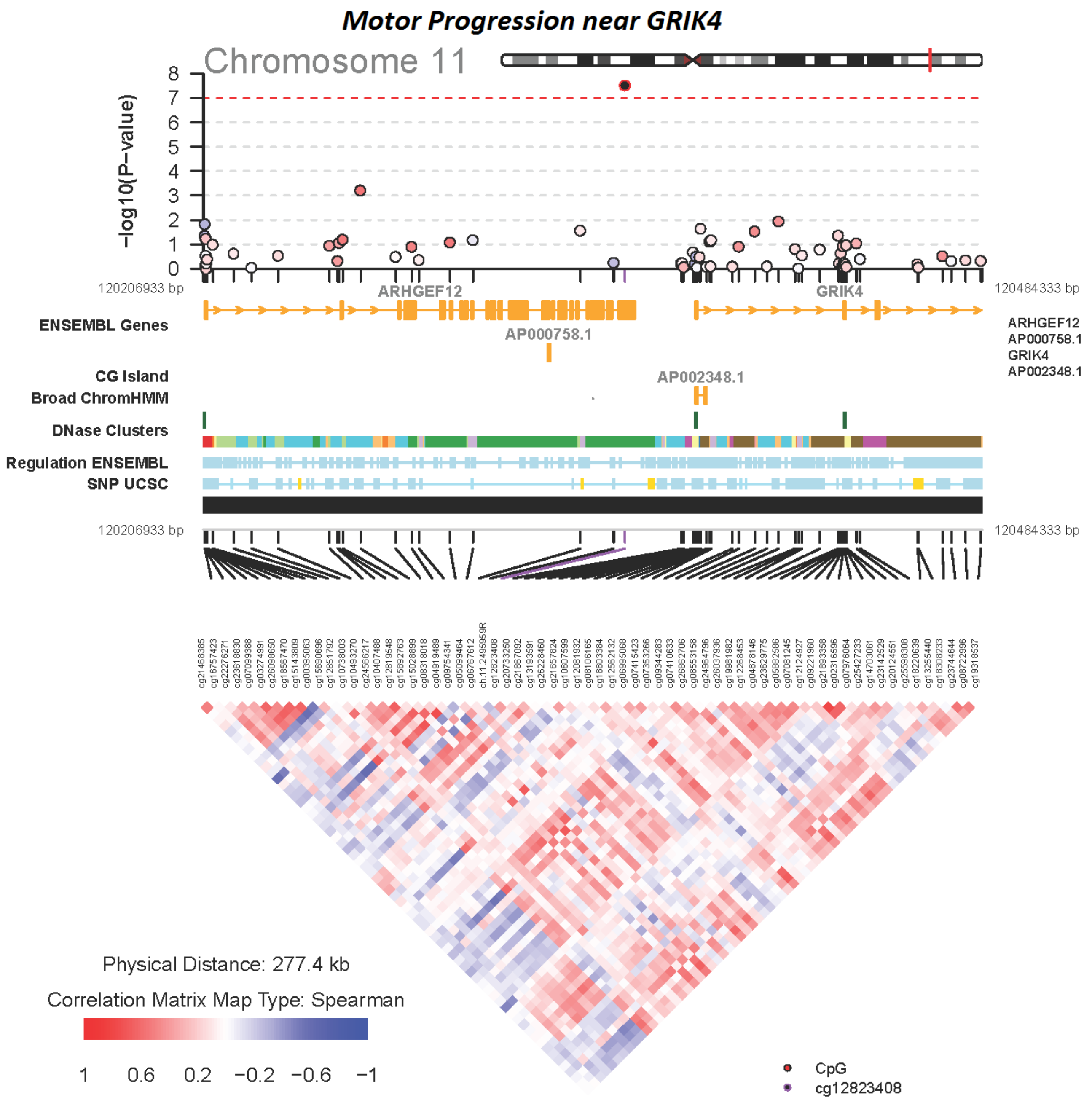

Motor Progression near GRIK4
Chromosome 11
-log10(P-value)
120206933 bp
120484333 bp
ARHGEF12
GRIK4
AP000758.1
AP002348.1
ENSEMBL Genes
CG Island
Broad ChromHMM
DNase Clusters
Regulation ENSEMBL
SNP UCSC
ARHGEF12
AP000758.1
GRIK4
AP002348.1
Physical Distance: 277.4 kb
Correlation Matrix Map Type: Spearman
1 0.6 0.2 −0.2 −0.6 −1
CpG
cg12823408

(C)

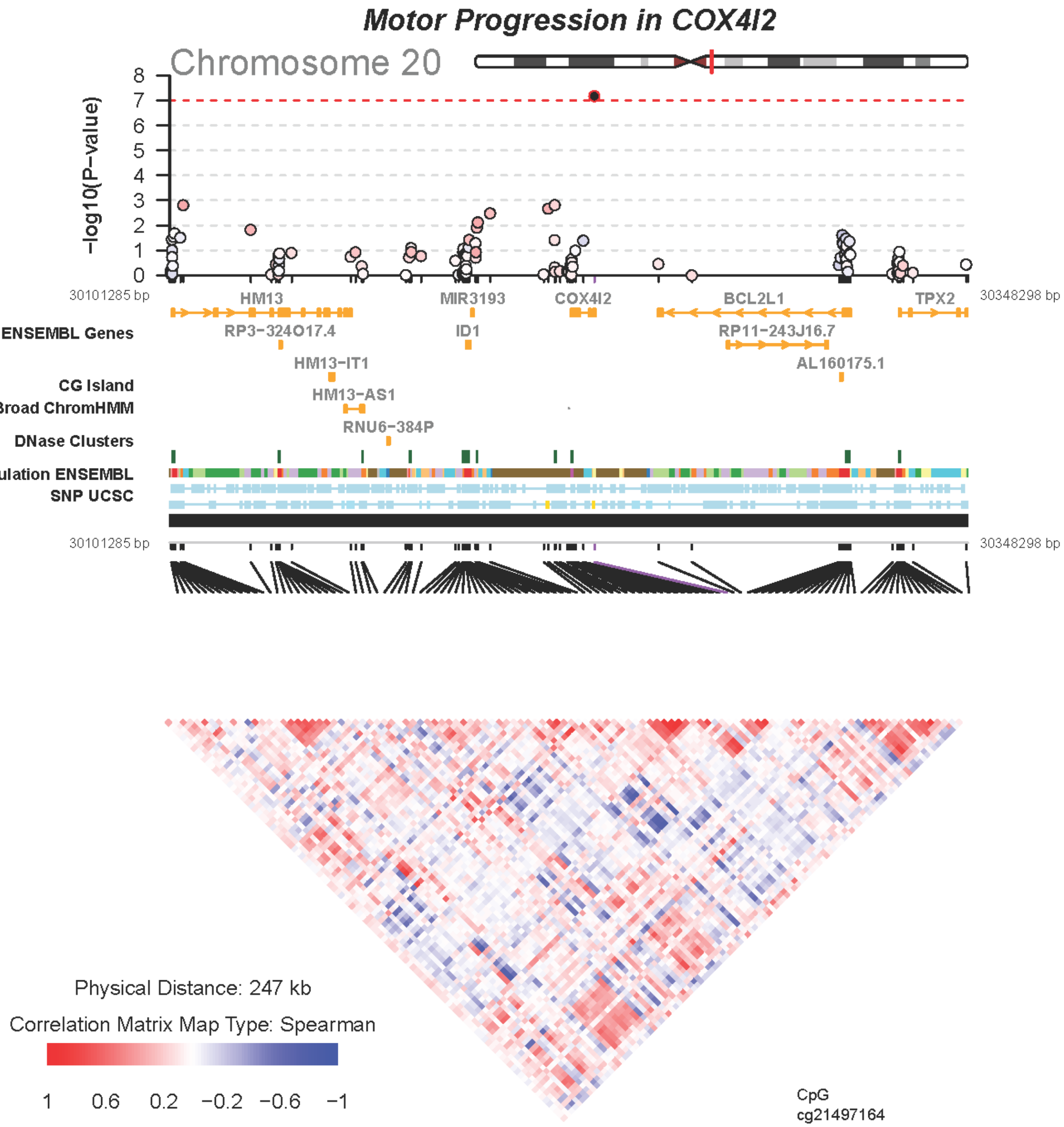
Motor Progression in COX4I2
Chromosome 20
-log10(P-value)
30101285 bp
30348298 bp
HM13
MIR3193
COX4I2
BCL2L1
TPX2
RP3-324O17.4
ID1
RP11-243J16.7
HM13-IT1
AL160175.1
HM13-AS1
RNU6-384P
ENSEMBL Genes
CG Island
Broad ChromHMM
DNase Clusters
Regulation ENSEMBL
SNP UCSC
Physical Distance: 247 kb
Correlation Matrix Map Type: Spearman
1 0.6 0.2 −0.2 −0.6 −1
CpG
cg21497164

Supplementary Fig. 19 Characters of patients with tetrabenazine medications in Enroll-HD data 1

The bar plots depict HD manifest patients who were treated with tetrabenazine compared to those without treatment in Enroll-HD data 1 (N=354). Comparisons are shown for: (A) CAP score, (B) CAG length, (C) age at methylation profile, and (D) motor score at methylation profile, respectively. The bar plots report the p-value of a non-parametric group comparison test (Kruskal-Wallis). The y-axis of the bar plots depicts the mean and one standard error. The vertical grey numbers underneath each bar report the number of individuals per treatment group.

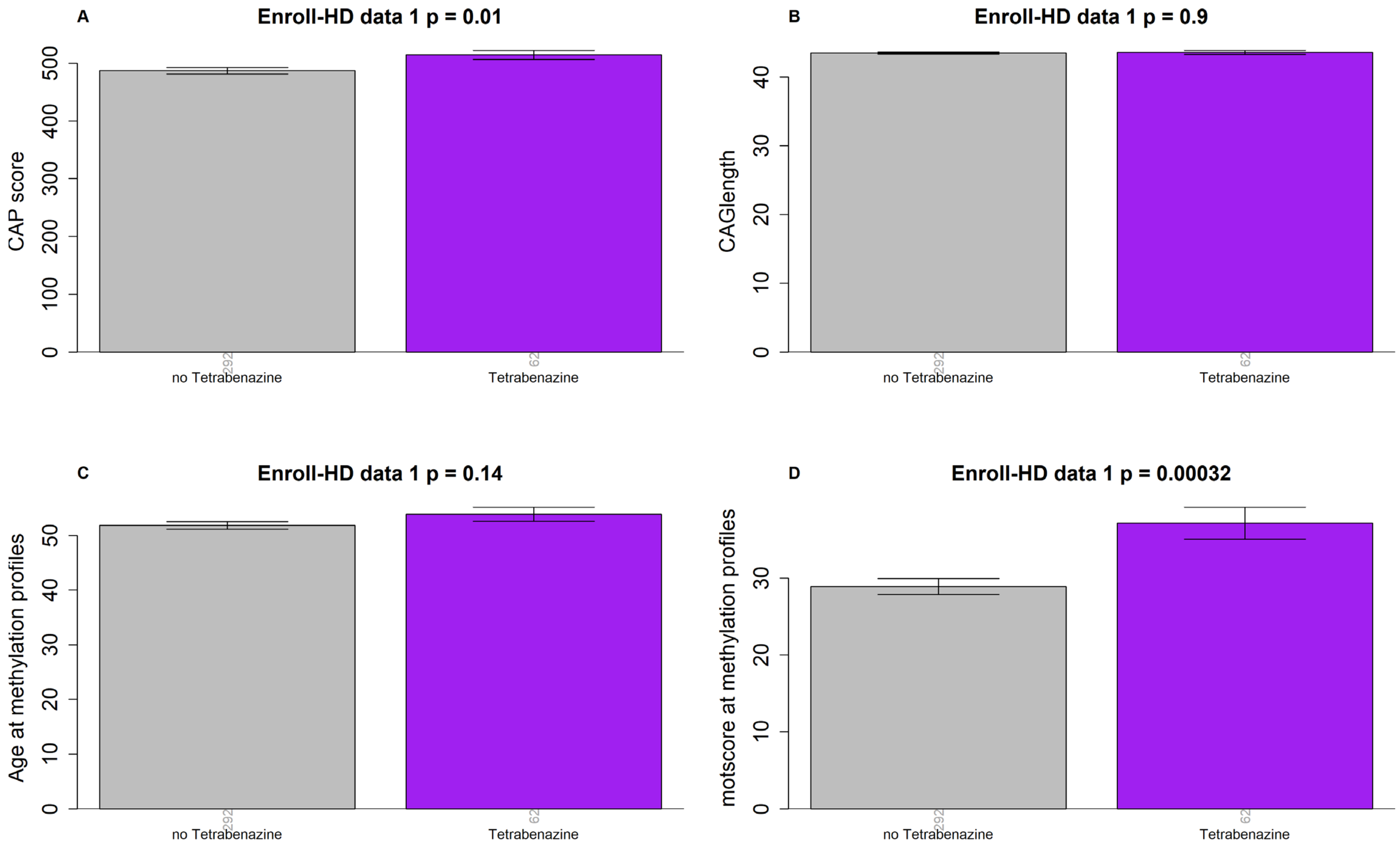

Supplementary Fig. 20 Patient characteristics of treated/untreated individuals in Enroll-HD data 2

The bar plots depict HD manifest patients who were treated with tetrabenazine compared to those without treatment in Enroll-HD data 2 (N=277). Comparisons are shown for: (A) CAP score, (B) CAG length, (C) age at the first methylation profile, and (D) motor score at the first methylation profile, respectively. The bar plots report the nominal (unadjusted) two sided p-value of a non-parametric group comparison test (Kruskal-Wallis). The y-axis of the bar plots depicts the mean and one standard error. The vertical grey numbers underneath each bar report the number of individuals per treatment group.

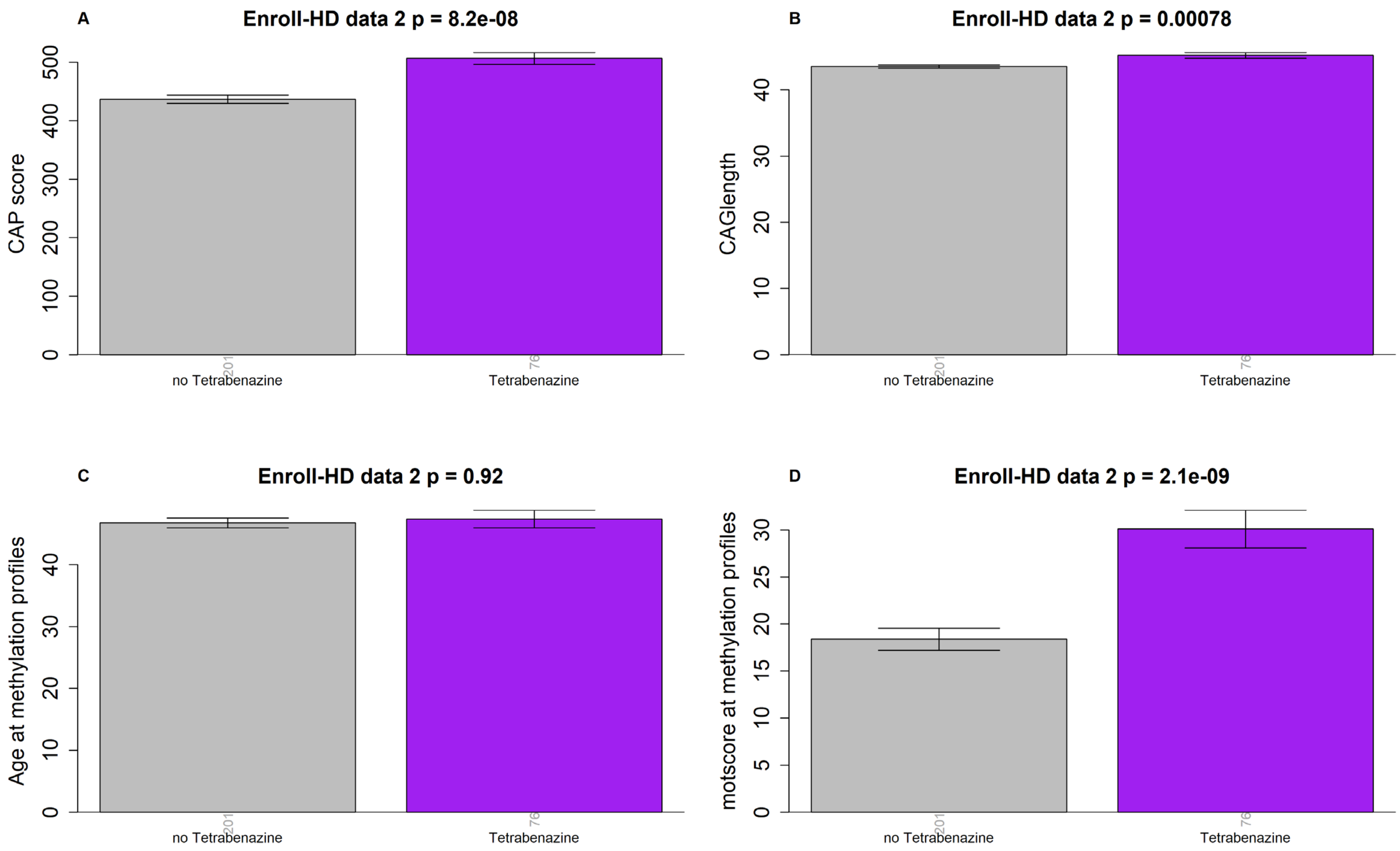

Supplementary Fig. 21 Association of tetrabenazine medications with DNA methylation levels

The bar plots depict associations between tetrabenazine treatment and DNA methylation levels in individuals from the Enroll-HD study. The methylation levels of the three genome-wide significant CpGs associated with motor progression (cg26919387 in the *PEX14* gene, cg12823408 near *GRIK4*, and cg21497164 in *COX4I2*) were examined. Panels (A-C) present the results using Enroll-HD data 1 (N=354) and panels (D-F) show the results using Enroll-HD data 2 (N=277) , respectively. The bar plots report the p-value of a non-parametric group comparison test (Kruskal-Wallis). The y-axis of the bar plots depicts the mean and one standard error. The vertical grey numbers underneath each bar report the number of individuals per treatment group.

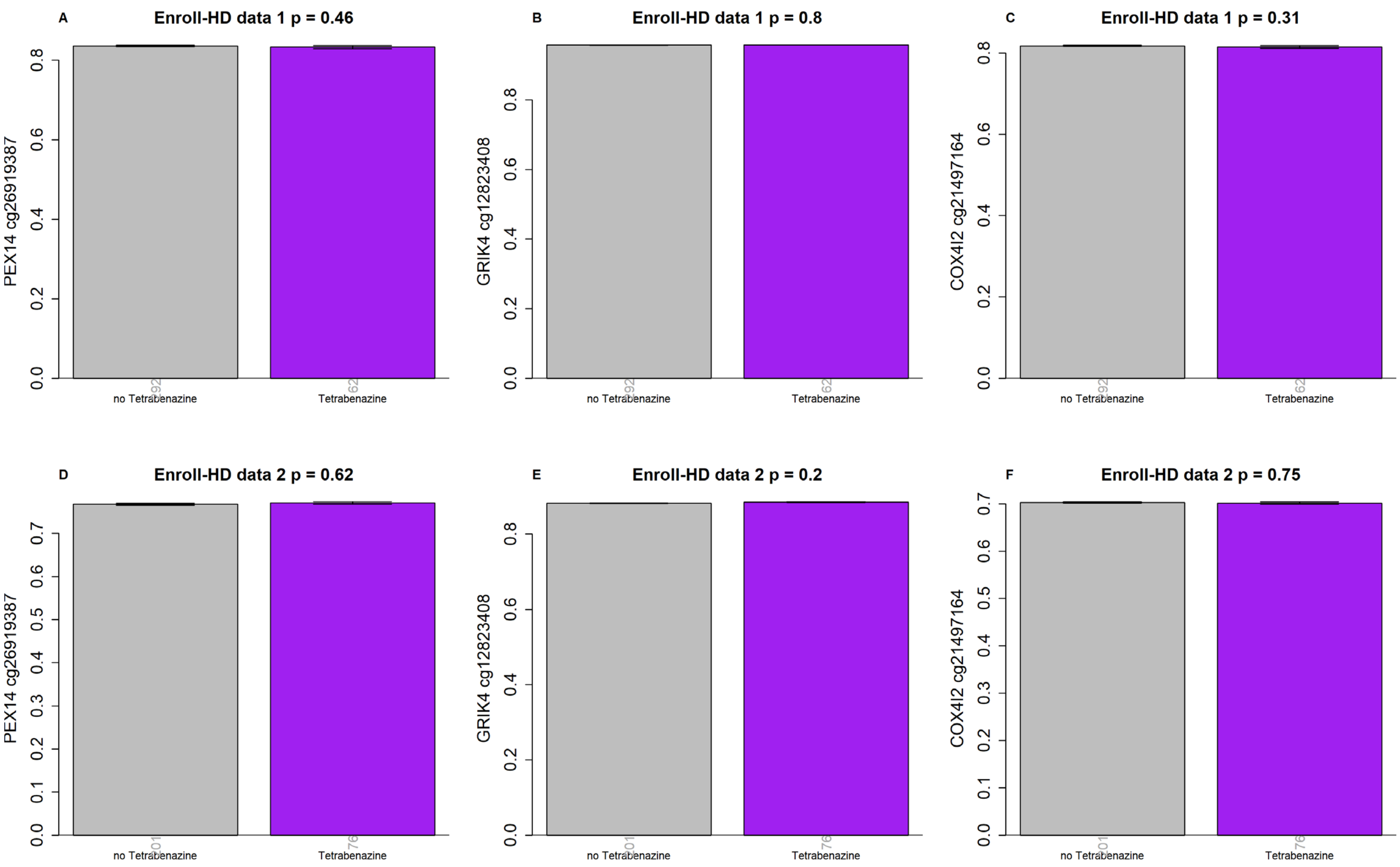

Supplementary Fig. 22 Epigenome-wide association study (EWAS) of tetrabenazine medication

Manhattan plots reports minus log(base)10-transformed P values versus chromosomal location for association with tetrabenazine medications in Enroll-HD data. We limited the analysis applied to the manifest patients included in our EWAS of HD progression study. Of the HD manifest patients, 18% and 27% have used tetrabenazine in data 1 (N=354) and data 2 (N=275), respectively. We performed EWAS of tetrabenazine medication in Enroll-HD data 1 and data 2, respectively size. The Manhattan plot below present the meta-EWAS that combined the association results across two Enroll-HD datasets based on Stouffer's method weighted by sample size. The blue lines correspond to suggestive level of significance (1.0E-5); the red lines correspond to genome-wide significant level P<1.0E-7. The most two significant CpGs are cg14647957 in Chr17 *RPTOR* (meta P=7.6E-6) and cg14647957 in Chr11 *PAX 6* (meta P=8.2E-6).

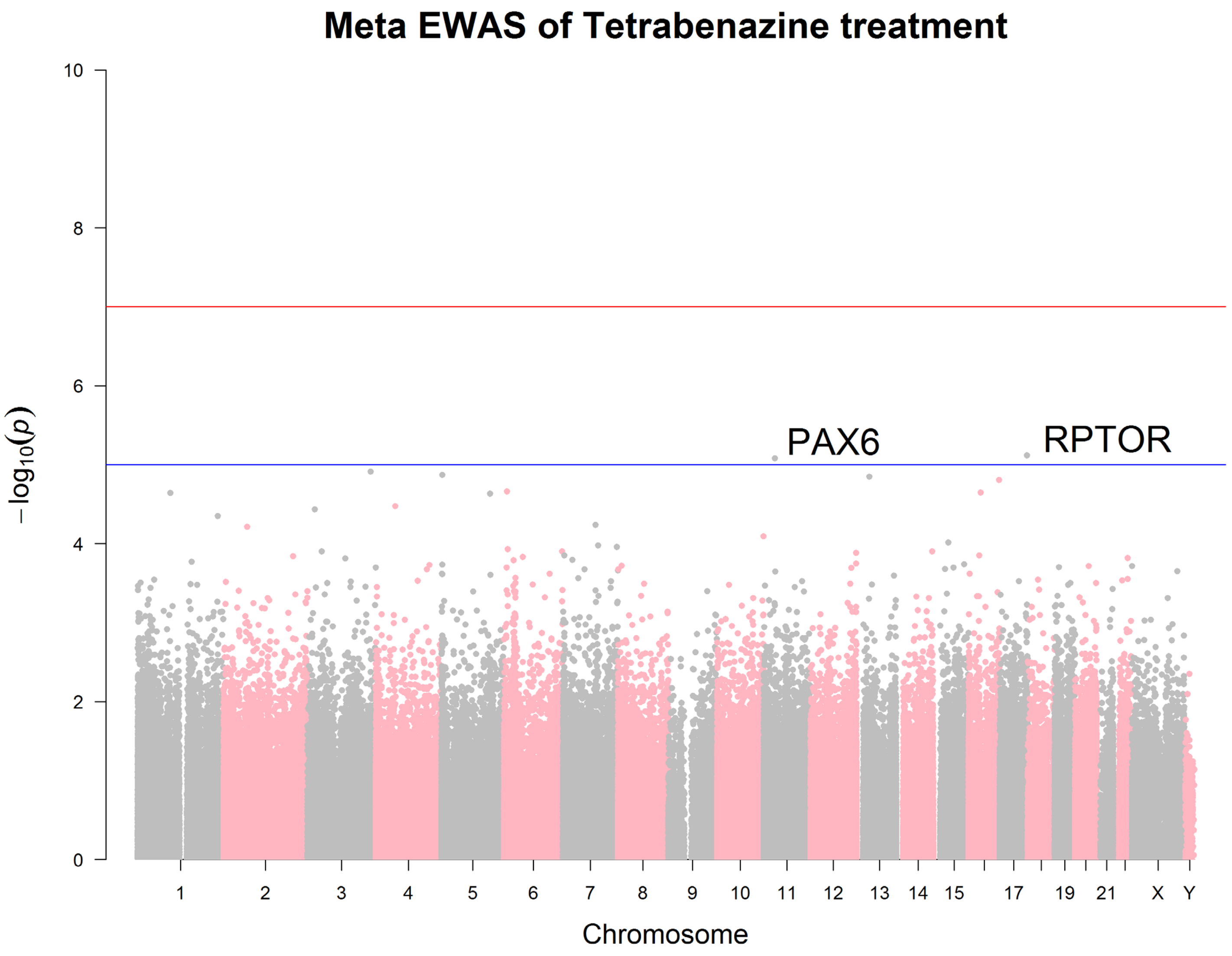

Supplementary Fig. 23 Mouse EWAS in *Htt* gene in brain data 1

We present mouse brain EWAS of the *Htt* gene based on Q175 (n=8) and Q20 mice (n=8). Methylation array profiles were based on reduced representation bisulfite sequencing (RRBS) analysis. Panels A & B depicted the association results in *Htt* gene (n ≥15) for cerebellum and striatum, respectively. Each point represents the association results of a CpG site with positive (negative) t-statistics marked in red (blue). Panel C) Scatter plot involving CpGs in the *Htt* gene region. Student T test statistic (for Q175 genotype status) in cerebellum (x-axis) versus Student T test statistic in the striatum. The dots (CpGs) are colored in red if the t-statistics have the same sign across the two brain regions. Panel D depicts the correlation of the t-statistics between cerebellum and striatum EWAS across whole genome (number of CpGs approximately 2.8 million). The CpGs in *Htt* are marked in red and text label ('Htt'). Panels C& D display the Pearson correlation coefficients and p values. The reported p-values in each plot are two sided and have not been adjusted for multiple comparisons.

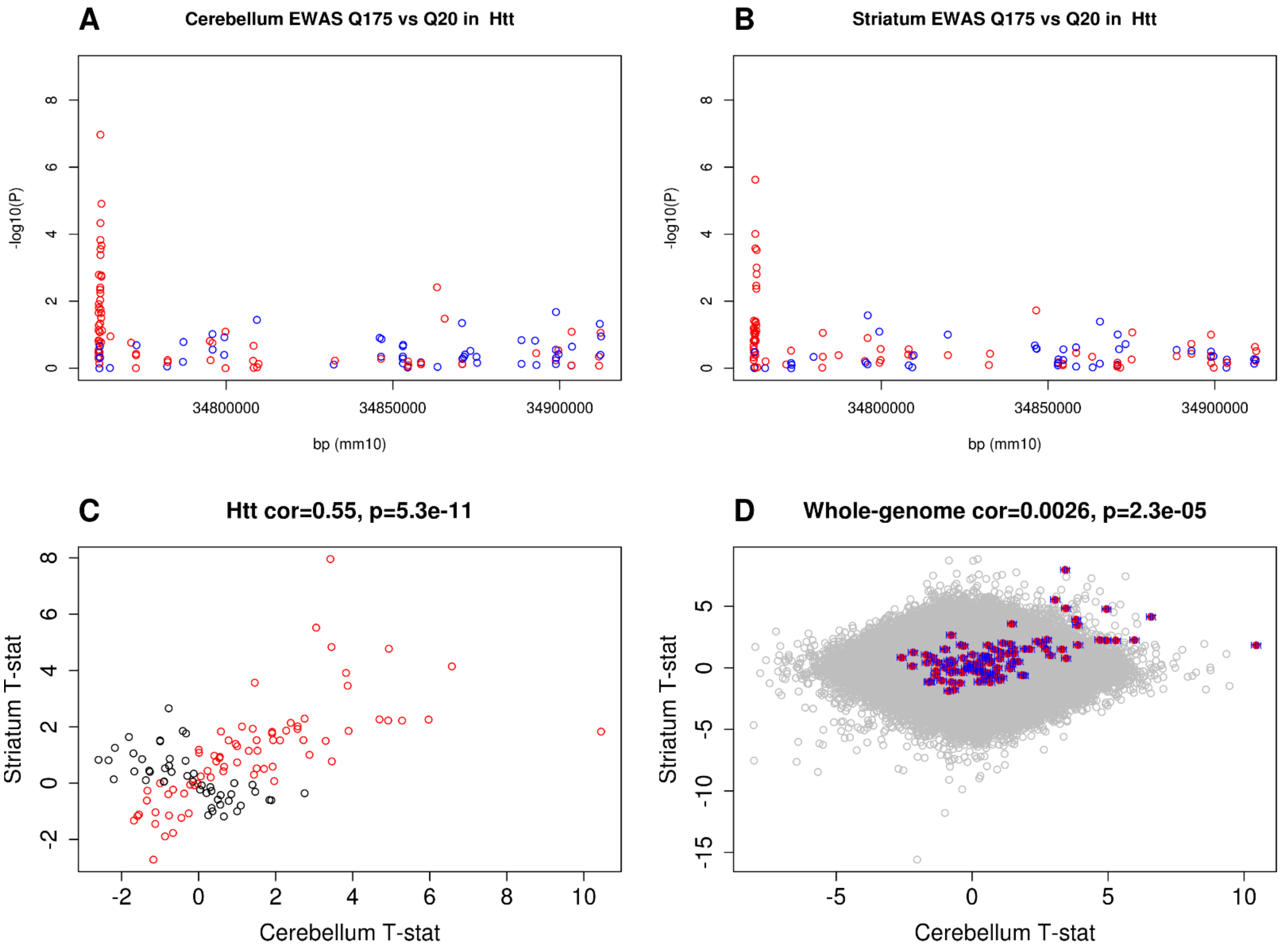

Supplementary Fig. 24 Mouse epigenome-wide association study (EWAS) in brain data 1

The Manhattan plots depict minus log(base)10-transformed P values versus chromosomal location for association with HD status using A) mouse cerebellum and B) mouse striatum. The nominal (unadjusted) two sided p values result from a Student T test. The blue lines correspond to suggestive level of significance (p<5.0E-6) in each panel; the red lines correspond to genome-wide significant level (p< 1.0E-7). In each panel, we tested the CpGs with at least 15 observations. DNA methylation arrays were profiled based on reduced representation bisulfite sequencing analysis (**RRBS**). In the cerebellum, the most significant (p=1.01E-7) CpG was located at 5:34762314 in Htt gene . In the striatum, two CpGs exhibited genome-wide significance (p<1.0E-7) and the most significant association in Htt was CpG 5:3476224 (p=2.4E-6).

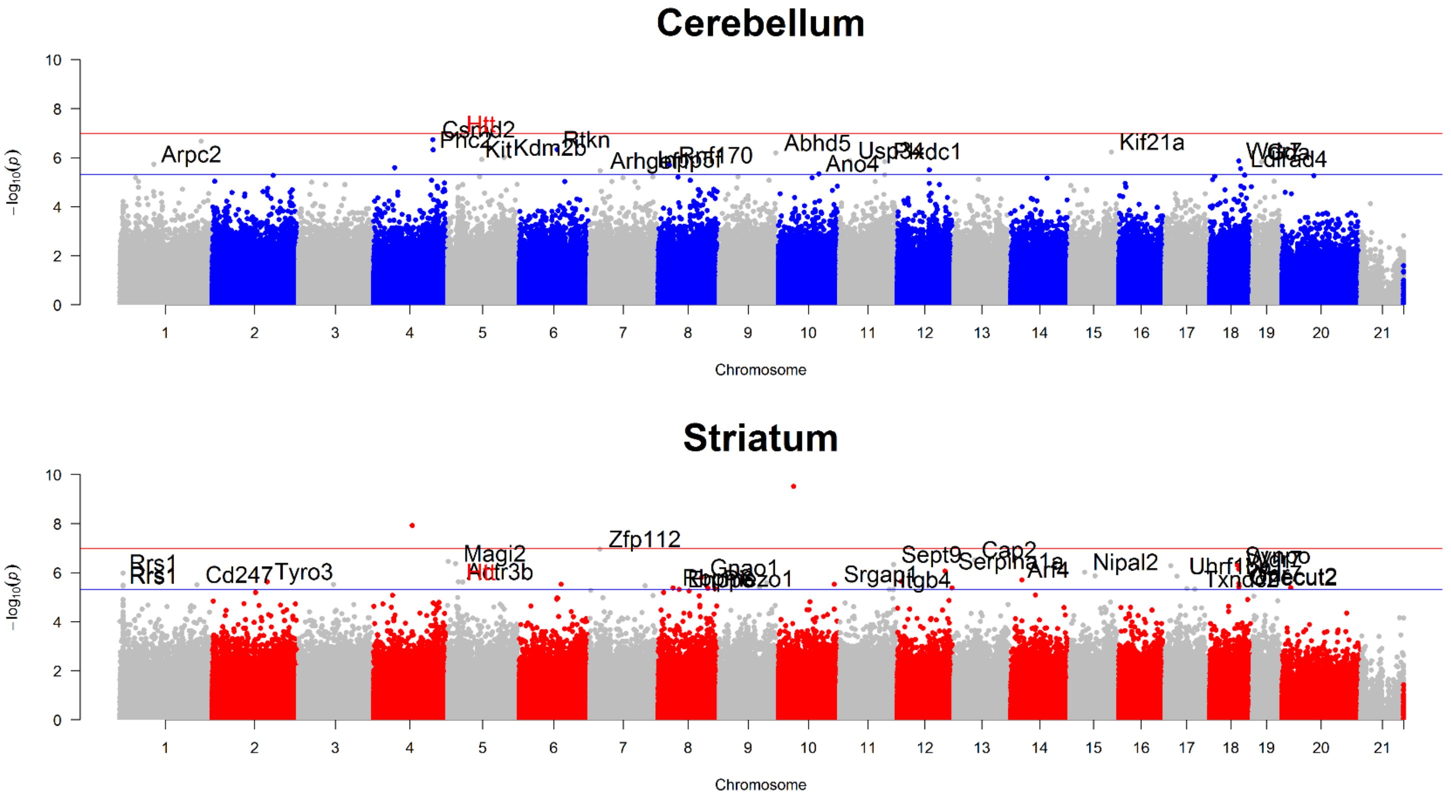

Supplementary Fig. 25 Mouse studies based on the custom methylation array.

We used the custom methylation array (HorvathMammalMethylChip) to study different mouse tissues. The top EWAS hit (probe cg12389415, in *HTT* exon 5, ENSMUST00000148953.7/15194, chromosome 15, mm10 starting coordinate 34795955) exhibits hypomethylation in A) the striatum, B) brain cortex, C) cerebellum, D) blood, and E) liver from six month old Q175 mice. The same statement applies to tail samples from F) six month old and G) 12 month old Q140 mice. The vertical grey numbers underneath each bar report the number of individuals per treatment group. The titles report nominal (unadjusted) two sided p-values from a two group comparison (Student T test). H) Mouse epigenome-wide association study (EWAS) in brain data 2. The Manhattan plots depict $-\log_{10}$--transformed p-values versus chromosomal location for association with HD status using mouse brain tissues. Methylation array profiles were based on results from a custom methylation array. We performed EWAS of HD status based on two-sided t-tests, using the CpG methylation levels profiled in blood, cerebellum, cortex, liver and striatum, respectively. DNA methylation arrays were profiled in a customized Illumina array. The Manhattan plot below present the meta-EWAS that combined the association results across cerebellum and striatum based on Stouffer's method. The blue lines correspond to genome-wide significant levels in each panel (p<1.0E-7); the red lines correspond to p<1.0E-8. The most significant association appears at *Htt* CpG 5:34795955 in mm10 assembly (meta p=3.2E-22). Integrating the EWAS results (by Stouffer's method) across all the available tissue types: the two brain regions, blood, cortex and liver, the strengthened the association result (p-value of 6.2E-45).

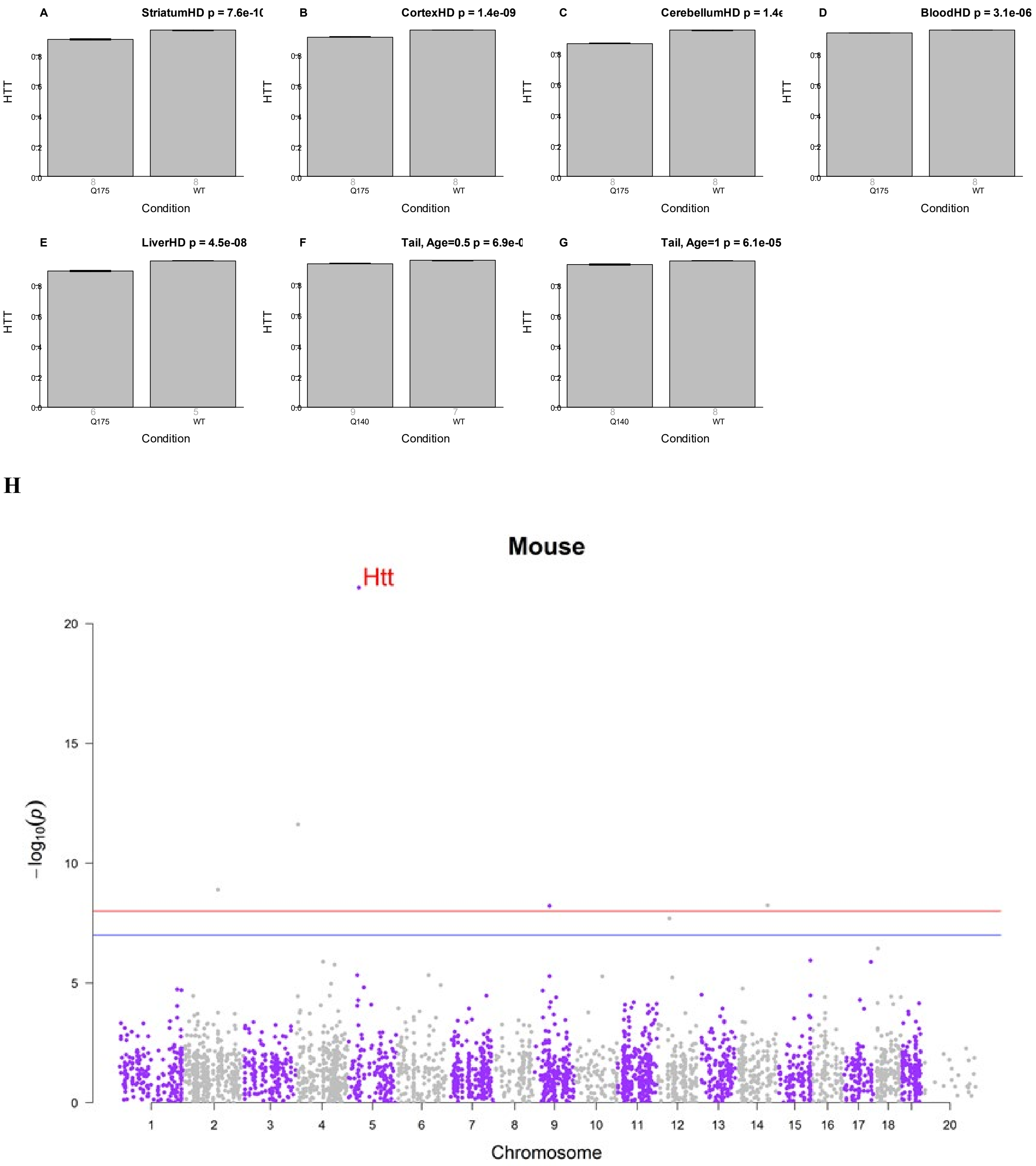
A
StriatumHD p = 7.6e-1(
B
CortexHD p = 1.4e-09
C
CerebellumHD p = 1.4ε
D
BloodHD p = 3.1e-06
E
LiverHD p = 4.5e-08
F
Tail, Age=0.5 p = 6.9e-(
G
Tail, Age=1 p = 6.1e-05
HTT
Q175
Q140
WT
Condition
H
Mouse
Htt
$-\log_{10}(p)$
Chromosome

Supplementary Tables

Supplementary Table 1. DNA Methylation samples mice and sheep

The table describes three DNA methylation datasets profiled in mice and sheep: 1) mouse data1 (N=32 samples) 1, 2) mouse data2 (N=32) and 3) sheep data (N=168). Each mouse dataset includes 8 mice from Htt KI lines Q175 and 8 mice from Q20 group, respectively. Brain tissues across cerebellum and striatum were profiled from each mouse in both datasets, yielding a total of 32 tissues. EWAS was performed in each tissue type using data1 and replicated in data2. In mouse data2, DNA methylation array analysis was also profiled in blood, liver and cortex tissues for each mouse, totaling 80 samples. In the sheep data, blood DNA methylation analysis was carried out in 84 HD transgenic sheep, age matched with 84 control sheep. HD transgenic sheep were generated by microinjection of a full-length human HTT cDNA containing 73 polyglutamine repeats under the control of the human promotor[15].

**Parameter**		**Mouse data1 (N=32)**		**Mouse data2 (N=80**)**		**Sheep (N=168)**
**DNA source**		Cerebellum	Striatum	Cerebellum	Striatum	blood
**No. Samples**	HD	8	8	8	8	84
	control	8	8	8	8	84
**Age***	HD	6 months	6 months	6 months	6 months	4.1 years [2.0, 7.0]
	control	6 months	6 months	6 months	6 months	4.1 years [2.0, 7.0]
**DNA methylation**	Array type	RRBS	RRBS	Mammalian array	Mammalian array	Mammalian array
	Normalization method	Not applicable	Not applicable	Sesame [16]	Sesame [16]	Sesame [16]

*age at dissection in mice; age parameter presented in the format of mean [min, max] in sheep.
**In Mouse data2, DNA methylation arrays were also profiled in blood, cortex and liver in addition to cerebellum and striatum, totaling 80 tissues.

Supplementary Table 2. Data used for estimating motor progression in the Enroll-HD study

This table describes the cases used for measuring motor progression in HD mutation carriers. The upper part of the table describes all the currently available observations in Enroll HD which could be used for estimating HD progression (using a mixed effects model). The lower panel describes the subset of cases for whom we had DNA methylation data available. We restricted our attention to two groups: manifest HD cases and pre-manifest HD mutation carriers. Before calculating the motor progression, we removed individuals with only a single visit and samples from manifest HD samples whose motor score was less than 5. We used all the observations from the manifest group and from the visits after conversion to manifest from the pre-manifest group for our downstream HD progression analysis.

**Parameter**	**Manifest**	**Pre-manifest**
Number of total observations	14,447	6,132
Number of individuals	4,963	2,088
Number of conversion* (%)	--	312 (15%)
Age at baseline (yrs)	53±12.5 [17, 91]	40±12.2 [17, 84]
Follow-up period (yrs)**	2.1±1.03 [1, 3]	2.1±1.08 [1, 3]
CAG length	44±4.07 [36, 71]	42.4±2.75 [36, 57]
Female	51%	60%
Limited to individuals with methylation array		
Number of individuals	318	194
Number of conversion* (%)	--	36 (18.5%)
Age at baseline (yr)	53±10.9 [20, 76]	41.3±11.6 [18, 73]
Follow-up period (yr)**	3.1±1.3 [2, 4]	3.5±1.05 [3, 4]
CAG length	44±2.6 [36, 51]	42±2.6 [37, 51]

*Number of conversion is in units of individuals.
continuous variables presented in the format of mean±SD [min, max] or mean±SD [5th, 95th] when marked with **.

Supplementary Table 3. Characteristics of Enroll-HD data 2 participants with longitudinal DNA methylation profiles

This table summarizes the characteristics of 357 cases from the Enroll-HD study. Each individual has two measures of DNA methylation array (Δage ~ 7.9 years apart). We stratified the analysis by disease status aligned with the first measure of DNA methylation. Cases with a motor score less than 5 were removed in the manifest group and the individuals with only one visit were entirely removed. We used all the observations from the manifest group and the visits after conversion to manifest from the pre-manifest group for our downstream HD progression analysis.

**Parameter**	**Manifest**	**Pre-manifest**
**Number of observations**	1577	1087
**Number of subjects**	204	153
**Number of conversion**	--	73
**Age* (yr)**	48.±11.8 [30, 70]	40±11.4 [23, 58]
**Follow-up period (yr)****	7.9±2.25 [3, 11]	7.7±2.27 [4, 11]
**CAG length**	44±3.8 [40, 52]	42±2.4 [39, 47]
**Female**	51%	55%

Follow-up period: years between the first visit where DNA methylation was measured and the last visit.
*mean±[min, max];
** mean± [5th, 95th]

Supplementary Table 4. Linear mixed and regression analysis for HD motor progression using Enroll-HD data 2

The table presents the results from 1) linear mixed model analysis of 1867 longitudinal motor scores across 278 manifest HD patients from Enroll data 2 and 2) linear regression model analysis of the random slopes of the 278 individuals estimated from the model in 1). The average follow-up was 7.8 years. The linear mixed models included a random intercept to account for intra subject variation, a random slope with respect to visit and gender, age aligned at the first profile of DNA methylation array, CAG repeat length, age at motor onset, education attainment, and visit as fixed effects. The empirical Bayes estimates of random slopes (N=278) were used as measures of HD motor progression. We adjusted the random slopes for all the confounders except visit for downstream analysis. The columns report the covariate name, regression coefficient, standard error, Student T-statistic, and two-sided Wald test p-value.

	Linear mixed				Linear regression			
**Parameter**	**Coef.**	**SE**	**T statistic**	**P**	**Coef.**	**SE**	**T statistic**	**P**
Intercept	-82.670	21.801	-3.792	1.55E-04	-16.782	3.517	-4.772	2.99E-06
Female	3.421	1.763	1.940	5.34E-02	0.080	0.279	0.287	7.74E-01
Age	2.468	0.160	15.436	4.90E-39	0.052	0.024	2.202	2.85E-02
CAG length	1.841	0.384	4.797	2.66E-06	0.338	0.062	5.436	1.22E-07
Age at motor onset	-2.212	0.181	-12.210	1.24E-27	-0.014	0.028	-0.516	6.06E-01
Education	0.007	0.715	0.009	9.93E-01	0.023	0.113	0.206	8.37E-01
Visit	4.380	0.198	22.086	1.78E-94				

Supplementary Table 5. Linear regression analysis for HD motor progression using Registry-HD data

The table presents the results from linear regression model analysis of the motor scores at the last visit using the Registry-HD manifest patients. The regression analysis was adjusted for gender, CAG repeat length, age at last visit, age at motor onset, age aligned at the first DNA methylation array analysis and education attainment. We used the residuals as measures of HD motor progression for downstream analysis. The columns report the covariate name, regression coefficient, standard error, Student T-statistic, and two-sided Wald test p-value.

Parameter	Coef.	SE	T statistic	P
Intercept	-86.141	31.812	-2.708	7.19E-03
Female	6.931	2.552	2.716	7.02E-03
CAG length	2.219	0.533	4.161	4.22E-05
Age	3.208	0.718	4.470	1.14E-05
Age at motor onset	-1.138	0.230	-4.950	1.28E-06
Age at DNA methylation	-1.612	0.700	-2.303	2.20E-02
education	-0.181	0.390	-0.464	6.43E-01

Supplementary Table 6. Multivariate linear models of DNAm age in Enroll-HD.

The table presents two multivariate models for regressing DNAm age (dependent variable) on chronological age, disease status and potential confounders including blood cell count estimates. Unlike model 1, model 2 contains 10 principal components based on the DNA methylation data. The columns report the covariate name, regression coefficient, standard error, Student T-statistic, and two-sided Wald test p-value. According to model 1, manifest HD cases exhibit an age acceleration of 1.4 years (=1.23/0.882) compared to controls. Model 2 reveals that manifest HD is associated with a significant increase in DNAm age ($p=2.9x10^{-3}$) even after adjusting for potential hidden confounders (captured by the 10 principal components).

**Outcome: DNAm Age**	**Model 1: No principal components**				**Model 2: With principal components**			
**Variable**	**Coef.**	**SE**	**T statistic**	**P**	**Coef.**	**SE**	**T statistics**	**P**
**Age**	0.882	0.014	64.281	**$<10^{-22}$**	0.767	0.016	48.48	**$<10^{-22}$**
**Pre-manifest HD vs control**	-0.086	0.418	-0.206	0.84	0.036	0.438	0.081	0.94
**Manifest HD vs control**	1.235	0.359	3.443	**$6.0x10^{-4}$**	1.063	0.356	2.986	**$2.9x10^{-3}$**
**Female**	-0.469	0.310	-1.510	0.13	0.083	0.291	0.286	0.77
**Education (Numeric)**	-0.071	0.148	-0.477	0.63	0.135	0.138	0.981	0.33
**BMI**	0.076	0.026	2.971	**$3.1x10^{-3}$**	0.052	0.024	2.187	**$2.9x10^{-2}$**
**Smoking pack years**	0.007	0.010	0.715	0.47	0.002	0.009	0.245	0.81
**CD4+T cell**	-15.16	2.616	-5.795	**$9.5x10^{-9}$**	11.082	4.210	2.633	**$8.6x10^{-3}$**
**CD8+T cell**	10.07	3.667	2.746	**$6.2x10^{-3}$**	2.564	4.798	0.534	0.59
**Granulocyte**	-2.52	2.159	-1.167	0.24	-2.485	3.614	-0.688	0.49
**B-cell**	-1.053	4.936	-0.213	0.83	3.799	4.754	0.799	0.42
**PC1**	--	--	--	--	-4091	1038	-3.941	**$8.8x10^{-5}$**
**PC2**	--	--	--	--	-65.16	29.598	-2.201	**$2.8x10^{-2}$**
**PC3**	--	--	--	--	-40.191	4.849	-8.288	**$4.3x10^{-16}$**
**PC4**	--	--	--	--	109.5	11.290	9.700	**$3.4x10^{-21}$**
**PC5**	--	--	--	--	23.77	8.450	2.814	**$5.0x10^{-3}$**
**PC6**	--	--	--	--	-4.531	5.037	-0.900	0.37
**PC7**	--	--	--	--	-13.06	4.203	-3.108	**$1.9x10^{-3}$**
**PC8**	--	--	--	--	-5.915	4.126	-1.434	0.15
**PC9**	--	--	--	--	-3.857	4.392	-0.878	0.38
**PC10**	--	--	--	--	-4.244	4.263	-0.996	0.32

P-values < 0.05 marked in bold.

Supplementary Table 7. Multivariate linear model of DNAm age in Registry-HD.

DNAm age (dependent variable) was regressed on chronological age, disease status and potential confounders including blood cell count estimates. The columns report the covariate name, regression coefficient, standard error, Student T-statistic, and two-sided Wald test p-value.

**Outcome: DNAmAge**		**Model Estimates**		
**Covariate**	**Coef**	**SE**	**T statistic**	**P-value**
**Age**	0.863	0.025	35.1	**8.9×10^{-84}**
**manifest HD**	-0.139	0.782	-0.2	0.86
**Female**	-1.005	0.655	-1.5	0.13
**Body mass index**	0.084	0.066	1.3	0.21
**Smoker (current)**	0.393	0.743	0.5	0.6
**CD4+ T cells**	-22.771	8.722	-2.6	**9.8×10^{-3}**
**CD8+ T cells**	2.653	12.921	0.2	0.84
**Granulocyte**	-19.206	7.570	-2.5	**1.2×10^{-2}**
**B cell**	-22.770	21.240	-1.1	0.29

P-values < 0.05 marked in bold

Supplementary Table 8. Association of Huntington disease motor progression with epigenetic age acceleration measures

The table below presents the associations of adjusted motor progression with age-adjusted epigenetic measures, based on robust correlation (biweight midcorrelation) analysis. We list the results for the analysis of 917 HD manifest patients from 1) Enroll HD data 1 (N=354) associated with short term follow-up (median ~ 3 years), 2) Enroll HD data 2 (N=275) associated with longer term follow-up (median ~8 years), and 3) Registry-HD cohort (N=288), respectively, and list the results (All, N=917) from the meta-analysis across the three studies using fixed effect models weighted by inverse variance. In Enroll-HD, motor progression measures were based on the random slopes in linear mixed model analysis, adjusted for age, CAG length, age onset of HD disease, and educational attainment. In Registry-HD, motor progression measures were based on the motor score evaluated in the last visit adjusted for age, CAG length, age onset of HD disease and educational attainment. Epigenetic measures were adjusted for age including 1) age adjusted DNAm age based on Horvath (AgeAccelerationResidual [AAR]) [3], 2) intrinsic epigenetic age acceleration (IEAA) derived on the basis of Horvath's DNAm age, 3) extrinsic epigenetic age acceleration derived on the basis Hannum's DNAm age [4], 4) age adjusted DNAmAgeSkinClock (AgeAccelSkinClock [AgeAccelSkin]) [5], 5) age-adjusted DNAm PhenoAge (AgeAccelPheno) [6], and 6) age-adjusted DNAm GrimAge [7] (AgeAccelGrim). The last three columns list the statistics associated bicor analysis (bicor correlation coefficient, standard error of the mean and $-\text{Log}_{10}$ transformed unadjusted two-sided p-value).

Data	Age-adjusted epigenetic biomarkers	bicor	SE	P
Enroll-HD data 1	AAR	0.13	0.05	**1.3E-02**
	IEAA	0.12	0.05	**2.9E-02**
	EEAA	0.08	0.05	1.5E-01
	AgeAccelSkin	0.03	0.05	5.7E-01
	AgeAccelPheno	0.08	0.05	1.5E-01
	AgeAccelGrim	0.08	0.05	1.5E-01
Enroll-HD data 2	AAR	0.02	0.06	7.1E-01
	IEAA	0.03	0.06	6.6E-01
	EEAA	0.08	0.06	2.1E-01
	AgeAccelSkin	0.08	0.06	1.8E-01
	AgeAccelPheno	0.07	0.06	2.5E-01
	AgeAccelGrim	0.03	0.06	6.0E-01
Registry	AAR	0.07	0.06	2.4E-01
	IEAA	0.06	0.06	2.8E-01
	EEAA	0.09	0.06	1.4E-01
	AgeAccelSkin	0.03	0.06	5.6E-01
	AgeAccelPheno	0.09	0.06	1.1E-01
	AgeAccelGrim	0.11	0.06	7.2E-02
All	AAR	0.08	0.03	**1.6E-02**
	IEAA	0.07	0.03	**2.7E-02**
	EEAA	0.08	0.03	**1.6E-02**
	AgeAccelSkin	0.05	0.03	1.6E-01
	AgeAccelPheno	0.08	0.03	**1.5E-02**
	AgeAccelGrim	0.07	0.03	**2.8E-02**
p-values < 0.05 marked in bold				

Supplementary Table 9. Characteristics of aggregated Enroll-HD data

The "aggregated Enroll-HD" dataset was generated from a subset of Enroll-HD data 1 cases (N=76) by averaging DNA methylation levels across chips, which effectively removed the chip effects. Row reports sample sizes, age at the time of the sample collection, and CAG length in HD cases.

	**Enroll-HD aggregated by chip**
**DNA source**	Buffy coat
**No. of samples**<br>**manifest HD, pre-manifest HD, controls**	33, 19, 24
**Age of HD cases (mean, min, max)**	49<br>[34, 59]
**Age of controls (mean, min, max)**	51<br>[40, 59]
**CAG length in HD cases (mean, min, max)**	43<br>[41, 45]
**CAG length in controls (mean, min, max)**	20<br>[18, 22]

Continuous parameters presented in the format of mean [min, max].

Supplementary Table 10 Associations of HTT methylations with CCG repeat length

Five multivariate linear regression models were performed to study the implications of CCG repeat length on *HTT* methylation, using the Registry-HD dataset upto 372 observations. Dependent variables included methylation levels of *HTT* cg22982173 in all the five models. Model 1 performed regression on two CCG length alleles and model 2 repeated the analysis adjusted two CAG length alleles. Model 3 repeated the analysis in model 1 limited to the 209 individuals with homozygosity for CCG_7 (where 7 denotes the length of CCG repeat). Model 4 performed regression analysis on two CAG length alleles plus HD disease status. Finally, we removed all participants carrying an atypical *HTT* structure as defined in our previous study [17] and re-examined the association of both CAG length alleles on methylation levels as presented in model 5 (N=350). We report nominal (i.e. not adjusted for multiple comparisons) two sided p values based on Student T-statistics.

First, although a rather modest association is observed in the Registry-HD population between *HTT* cg22982173 and the CCG repeat length on both the non-disease associated (allele 1, CCG1) and disease associated (allele 2, CCG2) and (r^2 = 0.033, P=0.0007), this association is not observed, after conditioning on CAG.short and CAG.long (p_{CCG1} = 0.92, p_{CCG2} = 0.37, N = 372, model 2). Second, sub-setting the Registry-HD cohort based on homozygosity for CCG_7 still reveals very strong associations between both CAG.short and CAG.long and *HTT* CpG methylation (r^2 = 0.30, p<2E-16, N = 209). Third, although HD status alone can predict *HTT* CpG methylation levels (r^2 = 0.12, P=7E-12), HD status is redundant to CAG.short and CAG.long in predicting *HTT* CpG methylation status ($p_{HDStatus}$ = 0.87, model 4), suggesting that CAG is the primary driver of methylation level differences between control and HD affected individuals. Fourth, removing all participants from the Registry-HD cohort carrying an atypical *HTT* repeat structure (**Methods**) [14], still reveals very strong associations between both CAG.long and CAG.short and *HTT* cg22982173 methylation (r^2 = 0.26, p E, 2.0E-16, model 5).

**Model 1**	**Multiple R-squared: 0.0383, Adjusted R-squared: 0.03309**			
	**Estimate**	**SE**	**T statistics**	**p-value**
CCG1	-3.5E-04	0.00016	-2.28	2.3E-02
CCG2	-6.3E-04	0.00022	-2.86	4.5E-03
**Model 2**	**Multiple R-squared: 0.2716, Adjusted R-squared: 0.2637**			
	**Estimate**	**SE**	**T statistics**	**p-value**
CAG.short	4.9E-04	6.04E-05	8.14	6.4E-15
CAG.long	1.2E-04	2.16E-05	5.52	6.3E-08
CCG1	-1.5E-05	1.41E-04	-0.11	9.2E-01
CCG2	2.0E-04	2.24E-04	0.90	3.7E-01
**Model 3 (CCG_7 only)**	**Multiple R-squared: 0.3066, Adjusted R-squared: 0.2999**			
	**Estimate**	**SE**	**T statistics**	**p-value**
CAG.short	5.6E-04	6.87E-05	8.15	3.5E-14
CAG.long	1.1E-04	3.15E-05	3.47	6.5E-04
**Model 4**	**Multiple R-squared: 0.2701, Adjusted R-squared: 0.2641 (164 observations deleted due to missing information)**			
	**Estimate**	**SE**	**T statistics**	**p-value**
CAG.short	4.9E-04	5.83E-05	8.42	8.8E-16
CAG.long	1.0E-04	4.69E-05	2.19	2.9E-02
HDStatusHD	1.9E-04	1.21E-03	0.16	8.7E-01
**Model 5**	**Multiple R-squared: 0.2583, Adjusted R-squared: 0.254**			
	**Estimate**	**SE**	**T statistics**	**p-value**
CAG.short	4.8E-04	6.00E-05	7.95	2.5E-14
CAG.long	1.1E-04	1.93E-05	5.78	1.7E-08

CCG1 denotes the number of CCG repeats on the CAG.short allele; CCG2 denotes the number of CCG repeats on the CAG.long allele.

Supplementary Table 11. Meta-analysis EWAS of HD progression

The table reports the top three genome-wide significant CpGs (meta-analysis p-value<10^{-7}). A positive (negative) value of the meta-analysis robust bi-weight correlation (bicor) indicated that the CpG was hypermethylated (hypomethylated) and associated with motor progression. Only manifest HD patients were used in this analysis. The EWAS results across Enroll-HD data 1(N=354), Enroll-HD data 2 (N=275), Registry-HD (N=288) were combined using fixed effect models weighted by inverse variance. Columns report chromosomal location (Chr), CpG name, gene symbol of nearest gene, bicor estimates (for Enroll-HD data 1, data 2, Registry-HD and combined (Meta) data, respectively), and corresponding nominal (unadjusted) two-sided P-values.

			bicor				P-value			
			Enroll HD		Reg.	Meta	Enroll HD		Reg.	Meta
Chr	CpG	Gene	Data1	Data2			Data1	Data2		
1	cg26919387	*PEX14*	-0.13	-0.25	-0.20	-0.19	1.8×10^{-2}	2.7×10^{-5}	5.5×10^{-4}	9.3×10^{-9}
11	cg12823408	*GRIK4**	-0.15	-0.22	-0.18	-0.18	5.2×10^{-3}	1.9×10^{-4}	1.9×10^{-3}	3.0×10^{-8}
20	cg21497164	*COX4I2*	-0.17	-0.09	-0.26	-0.18	9.9×10^{-4}	1.2×10^{-1}	1.0×10^{-5}	6.5×10^{-8}

*The CpG is located nearby the specified gene.

Supplementary Table 12. Enrichment analysis results of top 1000 HD related CpGs versus reference gene sets.

Selected results from enrichment analysis of a) top 1000 CpGs that are hypomethylated in HD, and b) the top 1000 CpGs that are hypermethylated in HD. Fisher's exact test (hypergeometric distribution) was used to assess the overlap with gene sets contained in the HDinHD online tool and the anRichment R package. Column "Gene set" indicates the gene set name. Columns "p-value", "Overlap", and "Gene set size", respectively refer to: the Fisher exact test p-value, number of the overlap genes between the top set and the gene set, and the number of genes in the gene set. Although, we investigated the top 1000 hyper and hypomethylated CpGs in HD, the effective gene sets were smaller: Only 684 CpGs hypomethylated CpGs in HD could be mapped to a neighboring gene. Only 540 CpGs hypermethylated CpGs in HD could be mapped to a neighboring gene.

**Gene set**	**p-value**	**Overlap**	**Gene set size**
**Enrichment of 684 hypomethylated CpGs**			
Soluble brain proteome of WT and HD R6/2 mice (Hosp via HDSigDB)	7.70E-28	430	8022
RNA polymerase II bound genes (Lee via Miller/HDSigDB)	2.90E-25	373	6696
Succinate dehydrogenase inhibitor 3-NP dependent expression in WT mouse striatal cells (Lee via HDSigDB)	2.50E-21	383	7261
Down-regulated, DE genes in STHdhQ111/Q111 cells upon heat shock (Riva via HDSigDB)	1.50E-20	355	6593
HD markers in mouse STHdh cells (Sadri-Vakili via HDSigDB)	7.60E-19	372	7180
Friedreich Ataxia iPSCs expression vs Miz4 stem cell line (Ku via HDSigDB)	3.30E-18	343	6480
DE genes in 15 wk R6/2 HD mice (Mielcarek via HDSigDB)	3.40E-18	446	9264
DE genes in 9 wk R6/2 HD mice (Mielcarek via HDSigDB)	2.40E-15	477	10440
Downregulated (FDR<0.1) in Stage2 vs. Stage 1 disease-associated microglia (DAM) (Keren-Shaul)	2.50E-15	208	3443
Myotonic Dystrophy 1 markers in human muscles (Welle via HDSigDB)	2.80E-15	523	11830
Myotonic Dystrophy 2 markers in human skeletal muscle (Krahe via HDSigDB)	1.30E-14	295	5584
Neuro2a Htt72Q-i vs Htt25Q-ni (Moily via HDSigDB)	4.30E-14	229	4025
DE genes in striatal cells of HdhQ111 HD mice (Lee via HDSigDB)	5.70E-14	307	5947

Enrichment of 540 hypermethylated CpGs			
Down-regulated, DE genes in STHdhQ111/Q111 cells upon heat shock (Riva via HDSigDB)	1.10E-25	306	6593
Down-regulated in the heart of mice inducibly expressing Celf1 for 12 h (Wang via HDSigDB)	2.70E-22	191	3420
Down-regulated genes in hippocampus of 6 mon HD Q175 mice vs Q20 (Aaronson via HDSigDB)	1.60E-18	138	2271
Up-regulated, Myotonic Dystrophy 1 markers in human muscles (Welle via HDSigDB)	8.00E-14	283	6972

Supplementary Table 13. Enrichment analysis results of top 1000 HD related CpGs versus reference gene sets.

Selected results from enrichment analysis of a) top 1000 CpGs that are hypomethylated in HD, and b) the top 1000 CpGs that are hypermethylated in HD. Fisher's exact test (hypergeometric distribution) was used to assess the overlap with gene sets contained in the HDinHD online tool and the anRichment R package. Column "Gene set origin" indicates the origin of the reference gene set (GO: Gene Ontology; User list enrichment: gene sets from the "userListEnrichment" function in the WGCNA R package; WGCNA of public HD expression data: Modules from in-house WGCNA analyses of publicly available data from Huntington's disease patients and mouse models; Literature gene sets: gene sets collected from the literature). Column "Gene set" indicates the gene set name; where the gene set is a WGCNA module, the name indicates the module label, highest enriched terms, and the dataset in which the module was identified. Columns "p-value", "Overlap", "Effective EWAS set size", and "Gene set size" give, respectively refer to, the Fisher exact test p-value, number of genes in the overlap of the CpG module and the gene set, the number of genes in the CpG module, and the number of genes in the gene set. Although, we investigated the top 1000 hyper and hypomethylated CpGs in HD, the effective gene sets were smaller: Only 684 CpGs hypomethylated CpGs in HD could be mapped to a neighboring gene. Only 540 CpGs hypermethylated CpGs in HD could be mapped to a neighboring gene.

Gene set origin	Gene set	p-value	Overlap	Effective EWAS set size	Gene set size
**Hypomethylated in HD: top 1000**					
User list enrichment	PolyQ_Combined_PPI_1139	$3.6x10^{-13}$	90	684	1111
WGCNA of public HD data	Coexp. Module 11: Astrocytes; Astrocyte probable; central glial substance IN Myelencephalon; regulation of macromolecule metabolic process [Consensus WGCNA across human caudate nucleus and cortex]	$8.7x10^{-11}$	82	684	1073
WGCNA of public HD data	Coexp. Module 14: nucleus [Consensus WGCNA of 2-, 6-, 10-month mouse HD knock-in model striatum [18]]	$2.9x10^{-10}$	63	684	744
WGCNA of public HD data	Coexp. Module 15: nucleus [WGCNA of data from visual cortex of HD patients and controls, data at ww.synapse.org, accession syn4505]	$1.2x10^{-9}$	72	684	939
WGCNA of public HD data	Coexp. Module 11: Astrocytes; Astrocyte probable; central glial substance IN Myelencephalon; biological regulation [Consensus WGCNA across human caudate nucleus, cerebellum and cortex]	$4.3x10^{-9}$	67	684	872
User list enrichment	Nucleolus_Localiz.741	$8.3x10^{-9}$	57	684	699
Literature gene sets	Significantly down with age in Thymus (AGEMAP [19])	$2.0x10^{-8}$	39	684	400
GO	RNA processing	$4.0x10^{-8}$	59	684	769
**Hypermethylated in HD: top 1000**					
WGCNA of hippocampus data from 2, 6, 10-month control mice	Coexp. Module 5: chr19p13; Up CD40 stimulation in MG AitGhezala; intracellular; Homeo.Synap.Plas All Combined	$7.8x10^{-54}$	241	542	3151
WGCNA of striatum data from 2, 6, 10-month control mice	Coexp. Module 2: synaptic transcriptome 2338; Neuron probable; Striatum; cell projection; Homeo.Synap.Plas All Combined	$3.6x10^{-22}$	112	542	1473
WGCNA of public HD data	Coexp. Module 19: GlutatmatergicSynapse; histone modification [WGCNA of data from visual cortex of HD patients and controls, data at ww.synapse.org, accession syn4505]	$8.3x10^{-21}$	71	542	699
WGCNA of striatum data from 2, 6, 10-month control mice	Coexp. Module 19: Cerebellum; Neuron probable; Cerebellar Cortex; neuron part	$7.5x10^{-20}$	48	542	345
WGCNA of public HD data	Coexp. Module 5: intracellular; Neuron probable [WGCNA of GSE32417, Q150 striatum [20]]	$2.4x10^{-18}$	103	542	1444

Supplementary Table 14. Intersection between top 1000 hypomethylated genes in HD and genes that were found from a protein-protein interaction network analysis of polyglutamine disorders

The top 1000 hypomethylated CpGs located near genes that were significantly enriched (hypergeometric $P=3.6x10^{-13}$) with genes that were also involved in a protein-protein interaction network analysis of polyglutamine disorders. The highly significant overlap is based on the following 90 overlapping genes

ACTN1, ADCY6, APBB1, ARHGDIA, ASNA1, CALM1, RUNX1, CBFA2T3, CD247, CDC25B, CDK5, CDKN2A, CNTFR, CREM, CRK, CRKL, CTBP1, DDB2, DLG1, DR1, E2F4, FDPS, FOS, GAPDH, GNB2, HTT, HDAC2, DNAJB2, HSPA1B, HSP90AB1, DNAJB1, HSPG2, IGF2R, ITGA5, ITPR3, LLGL1, NR3C2, NRL, OAZ1, PRDX1, RAF1, RAN, TRIM27, BRD2, RRM2, SRSF1, SMARCC1, SMARCD1, SNAP25, SP1, SSB, STAT3, STX5, TERF2, NR2C2, TRAF2, TSC1, UBE2G1, USP7, PRRC2A, BAG6, NRIP1, LTBP4, SLC4A4, SYNGAP1, PIAS2, USP10, QKI, TBPL1, TRAM2, PUM1, CKAP5, MED24, BAIAP2, SORBS1, KDM5B, ILVBL, NISCH, KIFAP3, PSME4, PHPT1, EFEMP2, QRICH1, NPLOC4, DHX37, ICE2, ARHGAP19, SGF29, C1QTNF1, TUBB.

Supplementary Table 15. GOMETH Enrichment analysis results of top 1000 HD related CpGs versus reference gene sets.

We listed the results with enrichment p-value (P<1.0eE-03) from GOMETH[21] of a) top 1000 CpGs that were hypomethylated in HD , and b) the top 1000 CpGs that were hypermethylated in HD. Wallenius' noncentral hypergeometric test was used to assess the overlap with all KEGG and GO terms, as performed in R 3.6 using the GOMETH function. The test takes into account the different number of probes per gene present on Illumina array (450k or Epic). The first three columns list the data base, GO ontology ("BP" - biological process, "CC" - cellular component, "MF" - molecular function) and gene set name. Columns "Gene set size", "DE", and "P" give, respectively refer to, the number of genes in the gene set, the number of genes that are differentiated methylated, and the enrichment p-value.

Data base	Ontology	Gene set	Gene set size	DE	P
**Enrichment of 1000 hypomethylated CpGs**					
GO:0070461	GO CC	SAGA-type complex	28	6.00	1.5E-04
GO:0005089	GO MF	Rho guanyl-nucleotide exchange factor activity	79	14.00	3.5E-04
GO:0005088	GO MF	Ras guanyl-nucleotide exchange factor activity	137	18.00	4.4E-04
GO:0005085	GO MF	guanyl-nucleotide exchange factor activity	214	23.00	4.6E-04
GO:0033276	GO CC	transcription factor TFTC complex	14	4.00	6.5E-04
GO:0051020	GO MF	GTPase binding	541	40.00	8.1E-04
**Enrichment of 1000 hypermethylated CpGs**					
GO:0003723	GO MF	RNA binding	1950	131.88	3.4E-06
GO:0016071	GO BP	mRNA metabolic process	877	68.83	1.8E-04
GO:0006396	GO BP	RNA processing	1456	79.17	1.9E-04
GO:0022613	GO BP	ribonucleoprotein complex biogenesis	502	41.00	3.2E-04
GO:0044403	GO BP	symbiont process	884	70.50	3.7E-04
GO:0070064	GO MF	proline-rich region binding	18	6.00	5.2E-04
GO:0005654	GO CC	nucleoplasm	3512	228.50	6.5E-04
GO:0044419	GO BP	interspecies interaction between organisms	932	70.50	8.6E-04
GO:0038093	GO BP	Fc receptor signaling pathway	241	21.00	9.1E-04
GO:0006403	GO BP	RNA localization	230	24.17	9.8E-04

Supplementary Table 16. Enrichment analysis results of top 500 HD progression related CpGs versus reference gene sets.

Selected results from enrichment analysis of a) top 500 CpGs that were hypomethylated in HD progression, and b) the top 500 CpGs that were hypermethylated in HD progression. Fisher's exact test (hypergeometric distribution) was used to assess the overlap with gene sets contained in the HDinHD online tool and the anRichment R package. Column "Gene set" indicates the gene set name. Columns "p-value", "Overlap", and "Gene set size" give, respectively refer to, the Fisher exact test p-value, number of the overlap genes between the top set and the gene set, and the number of genes in the gene set. The 500 hypomethylated CpGs can be mapped to 314 nearby genes while the 500 hypermethylated CpGs can be mapped to 358 nearby genes.

Our results showed that hypomethylated HD progression related CpGs were adjacent to genes that were significantly enriched in known HD relevant gene sets[22], such as downregulated genes in striatum of HD Q175 mice (P=2.28E-08) and down-regulated CAG length-dependent genes in 10 month old mouse striatum (P=1.25E-06). The hypermethylated HD progression related CpGs were near genes that were enriched with certain HD related gene sets[22], such as differentially expressed genes in striatal cells of HdhQ111 HD mice (P=8.08E-08), down-regulated in 12 week old R6/2 HD mice (P=2.47E-08), and HD markers in human caudate nucleus (P=4.10E-07). The other enrichment gene sets for hypomethylated/hypermethylated progression-associated CpGs included genes associated with mental disorders (P=2.95E-09), glucose metabolism disorders (P=7.46×10^{-8}), and differentially expressed genes in Friedrich Ataxia (p=1.06E-07).

Gene set	p-value	Overlap	Gene set size
**Enrichment of 500 hypomethylated CpGs**			
Up-regulated, Methylation profiles of human cerebral cortex vs liver (De Souza via HDSigDB)	1.00E-17	215	8419
Genes associated with Mental Disorders (CDC Genopedia v5.6)	2.95E-09	152	6124
Genes associated with Nervous System Diseases (CDC Genopedia v5.6)	3.52E-09	123	4589
Genes associated with Brain Diseases (CDC Genopedia v5.6)	4.22E-09	96	3254
Genes associated with Central Nervous System Diseases (CDC Genopedia v5.6)	5.80E-09	99	3419
Down-regulated genes in striatum of HD Q175 mice (Langfelder via HDSigDB)	2.28E-08	79	2554

Down-regulated, Methylation profiles of human cerebral cortex vs liver (De Souza via HDSigDB)	5.48E-08	207	9607
Genes associated with Glucose Metabolism Disorders (CDC Genopedia v5.6)	7.46E-08	100	3641
immune system process	3.53E-07	78	2676
Down-regulated genes in striatum of 10 mon HD Q140 mice vs Q20 (Aaronson via HDSigDB)	3.82E-07	62	1942
Down-regulated, CAG length-dependent genes in 10 mon mouse striatum (Langfelder via HDSigDB)	1.25E-06	74	2570
**Enrichment of 500 hypermethylated CpGs**			
Down-regulated, Methylation profiles of human cerebral cortex vs liver (De Souza via HDSigDB)	6.60E-19	264	9607
Methylation profiles of human cerebral cortex vs liver (De Souza via HDSigDB)	1.10E-12	312	13478
DE genes in 13 wk TIF-IA KO HD mice (Parkitna via HDSigDB)	2.68E-12	150	4750
DE genes in HdhQ111 striatal cells vs HdhQ7 (Pirhaji via HDSigDB)	1.37E-09	171	6139
HD markers in mouse STHdh cells (Sadri-Vakili via HDSigDB)	2.31E-09	191	7166
Soluble brain proteome of WT and HD R6/2 mice (Hosp via HDSigDB)	3.01E-09	207	8003
DE genes in STHdhQ111/Q111 cells upon heat shock (Riva via HDSigDB)	3.99E-09	249	10281
Down-regulated, DE genes in STHdhQ111/Q111 cells upon heat shock (Riva via HDSigDB)	5.56E-09	178	6587
DE genes in striatal cells of HdhQ111 HD mice (Lee via HDSigDB)	8.08E-09	164	5933
Genes down-regulated in HdhQ111 striatal cells vs HdhQ7 (Pirhaji via HDSigDB)	1.00E-08	103	3170
Downregulated (FDR<0.1) in Stage2 vs. Stage 1 disease-associated microglia (DAM) (Keren-Shaul)	1.58E-08	108	3412
Down-regulated in 12 wk R6/2 HD mice (Set1) (Kuhn via HDSigDB)	2.47E-08	70	1873
Down-regulated, DE genes in STHdhQ7/Q7 cells upon heat shock (Riva via HDSigDB)	5.95E-08	137	4806
protein binding	6.18E-08	257	10970
Friedreich Ataxia iPSCs expression vs SC41-MSC cell line (Ku via HDSigDB)	1.06E-07	206	8255

ChEA 2016: EP300 20729851 ChIP-Seq FORBRAIN MIDBRAIN LIMB HEART Mouse	1.15E-07	56	1411
Up-regulated, Methylation profiles of human cerebral cortex vs liver (De Souza via HDSigDB)	1.15E-07	209	8419
Friedreich Ataxia iPSCs expression vs SC31-MSC cell line (Ku via HDSigDB)	3.25E-07	208	8463
Down-regulated, STHdhQ7/Q7 vs STHdhQ111/Q111 cells upon heat shock (Riva via HDSigDB)	4.01E-07	117	4031
HD markers in human caudate nucleus (Durrenberger via HDSigDB)	4.10E-07	131	4676
DE genes in normal human brain vs other fetal tissues (Soragni via HDSigDB)	5.13E-07	152	5690
Down-regulated in Hsp90 inhibitor treated R6/2 HD mice vs WT mice (Labbadia via HDSigDB)	5.66E-07	105	3518
STHdhQ7/Q7 vs STHdhQ111/Q111 cells upon heat shock (Riva via HDSigDB)	7.18E-07	154	5816
Up-regulated, Friedreich Ataxia iPSCs expression vs SC31-MSC cell line (Ku via HDSigDB)	1.12E-06	116	4064

Supplementary Table 17. GOMETH Enrichment analysis results of top 500 HD progression related CpGs versus reference gene sets.

We listed the selected results from GOMETH enrichment analysis[21] of a) the top 500 CpGs that were hypomethylated in HD progression (P < 1.0eE-04), and b) the top 500 CpGs that were hypermethylated in HD progression ($P<1.0E^{-03}$). Wallenius' noncentral hypergeometric test was used to assess the overlap with all KEGG and GO terms, as performed in R 3.6 using the GOMETH function. The test takes into account the different number of probes per gene present on Illumina array (450k or Epic). The first three columns list the data base, GO ontology ("BP" - biological process, "CC" - cellular component, "MF" - molecular function) and gene set name. Columns "Gene set size", "DE", and "P" give, respectively refer to, the number of genes in the gene set, the number of genes that are differentiated methylated, and the enrichment p-value.

Our results showed that hypomethylated HD progression related CPGs tend to be located near genes that play a role in immune cell activation such as myeloid leukocyte activation (P=6.9E-07) and granulocyte activation (P=6.8E-06).

Data base	Ontology	Gene set	Gene set size	DE	P
Enrichment of 500 hypomethylated CpGs					
GO:0030141	GO CC	secretory granule	833	37.5	1.4E-07
GO:0002274	GO BP	myeloid leukocyte activation	651	31.5	6.9E-07
GO:0099503	GO CC	secretory vesicle	986	40.5	1.4E-06
GO:0045055	GO BP	regulated exocytosis	794	35	2.4E-06
GO:0060205	GO CC	cytoplasmic vesicle lumen	338	19.5	3.4E-06
GO:0031983	GO CC	vesicle lumen	339	19.5	3.9E-06
GO:0043312	GO BP	neutrophil degranulation	485	24.5	4.0E-06
GO:0002283	GO BP	neutrophil activation involved in immune response	488	24.5	4.2E-06
GO:0042119	GO BP	neutrophil activation	498	24.5	6.0E-06
GO:0002446	GO BP	neutrophil mediated immunity	499	24.5	6.1E-06
GO:0036230	GO BP	granulocyte activation	504	24.5	6.8E-06
GO:0043299	GO BP	leukocyte degranulation	534	25.5	6.9E-06
GO:0034774	GO CC	secretory granule lumen	321	18.5	7.3E-06
GO:0006887	GO BP	exocytosis	903	37.5	9.6E-06
GO:0002275	GO BP	myeloid cell activation involved in immune response	544	25.5	1.0E-05
GO:0002444	GO BP	myeloid leukocyte mediated immunity	551	25.5	1.2E-05
GO:0031410	GO CC	cytoplasmic vesicle	2314	72	2.0E-05
GO:0097708	GO CC	intracellular vesicle	2317	72	2.1E-05
GO:0045730	GO BP	respiratory burst	37	6	2.4E-05
GO:0044433	GO CC	cytoplasmic vesicle part	1504	51.5	2.7E-05
GO:0002376	GO BP	immune system process	3193	82	4.3E-05

GO:0006959	GO BP	humoral immune response	356	13	5.8E-05
GO:0001775	GO BP	cell activation	1440	47.5	6.9E-05
GO:0019730	GO BP	antimicrobial humoral response	122	8	9.5E-05
**Enrichment of 500 hypermethylated CpGs**					
GO:0007005	GO BP	mitochondrion organization	530	28	1.0E-04
path:hsa04662	KEGG	B cell receptor signaling pathway	82	9	2.5E-04
GO:0070201	GO BP	regulation of establishment of protein localization	746	36	2.6E-04
GO:0042826	GO MF	histone deacetylase binding	111	11.5	3.0E-04
GO:0017004	GO BP	cytochrome complex assembly	34	5	5.4E-04
GO:0034329	GO BP	cell junction assembly	241	18	9.7E-04

Supplementary References

1. Unified Huntington's Disease Rating Scale: reliability and consistency. Huntington Study Group. *Mov Disord* **11**, 136-42 (1996).
2. Losekoot, M. *et al.* EMQN/CMGS best practice guidelines for the molecular genetic testing of Huntington disease. *Eur J Hum Genet* **21**, 480-6 (2013).
3. Horvath, S. DNA methylation age of human tissues and cell types. *Genome Biol* **14**, R115 (2013).
4. Hannum, G. *et al.* Genome-wide methylation profiles reveal quantitative views of human aging rates. *Mol Cell* **49**, 359-367 (2013).
5. Horvath, S. *et al.* Epigenetic clock for skin and blood cells applied to Hutchinson Gilford Progeria Syndrome and ex vivo studies. *Aging (Albany NY)* **10**, 1758-1775 (2018).
6. Levine, M.E. *et al.* An epigenetic biomarker of aging for lifespan and healthspan. *Aging (Albany NY)* **10**, 573-591 (2018).
7. Lu, A.T., Quach, A., Ferrucci, L., Assimes, T. & Horvath, S. DNA methylation GrimAge strongly predicts lifespan and healthspan. *accepted by Aging* (2019).
8. al., L.e. DNA methylation-based estimator of telomere length. *reviewed under Nat Commun* (2019).
9. Martin, T.C., Yet, I., Tsai, P.C. & Bell, J.T. coMET: visualisation of regional epigenome-wide association scan results and DNA co-methylation patterns. *BMC Bioinformatics* **16**, 131 (2015).
10. Horvath, S. *et al.* Huntington's disease accelerates epigenetic aging of human brain and disrupts DNA methylation levels. *Aging* **8**, 1485-1512 (2016).
11. Horvath, S. *et al.* Huntington's disease accelerates epigenetic aging of human brain and disrupts DNA methylation levels. *Aging (Albany NY)* **8**, 1485-512 (2016).
12. Wright, G.E.B. *et al.* Length of uninterrupted CAG, independent of polyglutamine size, results in increased somatic instability, hastening onset of Huntington disease. *American Journal of Human Genetics* **104**, 1116–1126 (2019).
13. Genetic Modifiers of Huntington’s Disease Consortium *et al.* CAG repeat not polyglutamine length determines timing of Huntington’s disease onset. *Cell* **178**, 887–900 (2019).
14. Ciosi, M. *et al.* A genetic association study of glutamine-encoding DNA sequence structures, somatic CAG expansion, and DNA repair gene variants, with Huntington disease clinical outcomes. *EBioMedicine* (2019).
15. Jacobsen, J.C. *et al.* An ovine transgenic Huntington's disease model. *Hum Mol Genet* **19**, 1873-82 (2010).
16. Zhou, W., Triche, T.J., Jr, Laird, P.W. & Shen, H. SeSAMe: reducing artifactual detection of DNA methylation by Infinium BeadChips in genomic deletions. *Nucleic Acids Research* **46**, e123-e123 (2018).
17. Ciosi, M. *et al.* A genetic association study of glutamine-encoding DNA sequence structures, somatic CAG expansion, and DNA repair gene variants, with Huntington disease clinical outcomes. *EBioMedicine* **48**, 568-580 (2019).
18. Langfelder, P. *et al.* Integrated genomics and proteomics define huntingtin CAG length-dependent networks in mice. *Nat Neurosci* **19**, 623-33 (2016).
19. Zahn, J. *et al.* AGEMAP: a gene expression database for aging in mice. *PLoS Genet* **3**, e201 (2007).
20. Giles, P. *et al.* Longitudinal analysis of gene expression and behaviour in the HdhQ150 mouse model of Huntington's disease. *Brain Res Bull* **88**, 199-209 (2012).
21. Phipson, B., Maksimovic, J. & Oshlack, A. missMethyl: an R package for analyzing data from Illumina’s HumanMethylation450 platform. *Bioinformatics* **32**, 286-288 (2015).
22. Langfelder, P. *et al.* Integrated genomics and proteomics define huntingtin CAG length-dependent networks in mice. *Nature neuroscience* **19**, 623-633 (2016).
