# Supplementary material for: DNA methylation study of Huntington’s disease and motor progression in patients and in animal models
Source: Nat Commun. 2020 Sep 10;11:4529. doi: 10.1038/s41467-020-18255-5 (PMC7484780; doi:10.1038/s41467-020-18255-5)
Supplement: Supplementary file 2 — Description of Additional Supplementary Files [file 41467_2020_18255_MOESM2_ESM.pdf]

Description of Additional Supplementary Files

File Name: Supplementary Data 1
Description: Top hits EWAS of HD disease status across human, mouse and sheep

File Name: Supplementary Data 2
Description: Series of Multivariate regression analysis of joint effect of CAG length alleles on HTT methylation

File Name: Supplementary Data 3
Description: Top hits EWAS of HD motor progression across Enroll-HD and Registry-HD

File Name: Supplementary Data 4
Description: CpGs close to the CAG expansion are hypermethylated in HD

File Name: Supplementary Data 5
Description: Contribution to the collection of the Enroll-HD data. Individuals who contributed to the collection of the Enroll-HD data are listed in the pdf downloaded from https://www.enroll-hd.org/acknowledgments/
