# Supplementary material for: DNA methylation study of Huntington’s disease and motor progression in patients and in animal models
Source: Nat Commun. 2020 Sep 10;11:4529. doi: 10.1038/s41467-020-18255-5 (PMC7484780; doi:10.1038/s41467-020-18255-5)
Supplement: Supplementary file 7 — Supplementary Data 5 [file 41467_2020_18255_MOESM7_ESM.pdf]

PDS4 | 2018-10-R1

Periodic Dataset 4

Acknowledgement list

Version 2018-10-R1

Enroll-HD

A worldwide observational study for Huntington's disease families

A CHDI Foundation Project

Site	Country	Name
AarhusUnivHosp	Denmark	Anette Torvin Møller
AarhusUnivHosp	Denmark	Charlotte Germann Odgaard
AlbanyMedCol	USA	Era Hanspal
AlbanyMedCol	USA	Sharon Evans
AssistPubHospParis	France	Anne-Catherine Bachoud-Levi
AssistPubHospParis	France	Celine Joannet
AucklandCityHosp	New Zealand	Richard Roxburgh
AucklandCityHosp	New Zealand	Virginia Hogg
AucklandCityHosp	New Zealand	Lisa Fraser
AugustaUniv	USA	John Morgan
AugustaUniv	USA	Paula Jackson
AvonWiltMenHeaPartTr	UK	Lesley Gowers
AvonWiltMenHeaPartTr	UK	Carol Hall
AyrshireHealthBoard	UK	Margo Henry
AyrshireHealthBoard	UK	Tim Johnston
AziendaOspedSanAndre	Italy	Michela Ferraldeschi.
AziendaOspedSanAndre	Italy	Giovanni Ristori
AziendaOspedSanAndre	Italy	Silvia Romano
BaylorCollMed	USA	Danielle Peacock
BaylorCollMed	USA	Christine Hunter, RN
BaylorCollMed	USA	Joseph Jankovic, MD
BeaumontHosp	Ireland	Ms Fiona O'Donovan
BeaumontHosp	Ireland	Orla Hardiman
BeaumontHosp	Ireland	Sinead Maguire
BeaumontHosp	Ireland	Samira Bouazzaoui
BeaumontHosp	Ireland	Niall Pender
BeauVallon	Belgium	Lamia Guettat
BeauVallon	Belgium	Jean Marie Warzee
BirmSolNHSFounTrust	UK	Ellice Parkinson
BirmSolNHSFounTrust	UK	Hugh Rickards
BordeauxUnivHosp	France	Cyril Goizet
BordeauxUnivHosp	France	Marie-Pierre Baudier
BostonMedCtr	USA	Raymond James
BostonMedCtr	USA	Marie Saint-Hilaire
BurgosFoun	Spain	Esther Cubo
BurgosFoun	Spain	Natividad Marisccal
CardiffUniv	UK	Anne Rosser
CardiffUniv	UK	Rebecca Cousins
CardiffUniv	UK	Thomas Massey
CardiffUniv	UK	Duncan McLauchlan
CardiffUniv	UK	Monica Busse
CCFLouRuvo	USA	Zoltan Mari, MD
CCFLouRuvo	USA	Hooman Honar

Site	Country	Name
CenterMovDis	Canada	Teena Kailasanathan
CenterMovDis	Canada	Kimberly Thompson
CenterMovDis	Canada	Mark Guttman
CentHospUnivMontreal	Canada	Lyne Jean
CentHospUnivMontreal	Canada	Sylvain Chouinard
CentManHospFounTrust	UK	Helen Chadwick
CentManHospFounTrust	UK	David Craufurd
CentManHospFounTrust	UK	Dawn Rogers
CentManHospFounTrust	UK	Iris Trender-Gerhard
CentManHospFounTrust	UK	Liz Howard
CETRAM	Chile	Maria Consuelo Moos
CETRAM	Chile	Pedro Chana
CETRAM	Chile	Natalia Rojas
ClermontFerrandHosp	France	Ana-Raquel Marques
ClermontFerrandHosp	France	Dihya Benghadid
ClevelandClinicFoun	USA	Chris Firestone
ClevelandClinicFoun	USA	Anwar Ahmed
ClinTriCtrMaastricht	Netherlands	Mayke Oosterloo
ClinTriCtrMaastricht	Netherlands	Joelle Sondeijker
ColoSpringsNeuroAssoc	USA	Christen Kutz
ColoSpringsNeuroAssoc	USA	Alicia (Lisa) Deschaine
ColumbiaUniv	USA	Hiral Shah
ColumbiaUniv	USA	Ashwini Rao
ColumbiaUniv	USA	Lori Quinn
ColumbiaUniv	USA	Ronda Clouse
ColumbiaUniv	USA	Massood Manoochehri
ColumbiaUniv	USA	Karen Marder
ColumbiaUniv	USA	Paula Wasserman
CooperHealth	USA	Amy Colcher
CooperHealth	USA	Justin Frisby
CopernicusPodLec	Poland	Agnieszka Konkel
CopernicusPodLec	Poland	Witold Soltan
CrucesHosp	Spain	Juan Carlos Gómez Esteban
CrucesHosp	Spain	Maria Angeles Acera Gil
CtrHospUnivAmiens	France	Pierre Krystkowiak
CtrHospUnivAmiens	France	Stéphanie Blin
CtrHospUnivAngers	France	Christophe Verny
CtrHospUnivAngers	France	Audrey Olivier
CtrHospUnivMontpellier	France	Cecilia Marelli
CtrHospUnivMontpellier	France	Diana Ban
DukeUniv	USA	Lisa Gauger
DukeUniv	USA	Burton Scott
EmoryUniv	USA	Elaine Sperin

Site	Country	Name
EmoryUniv	USA	Jaime Hatcher-Martin
EmoryUniv	USA	Stewart Factor
Evergreen Health	USA	Pinky Agarwal
Evergreen Health	USA	Anna Fierro
FifeHealthBoard	UK	Michael Armanyous
FifeHealthBoard	UK	Nicola Johns
FundHospUnivLaFe	Spain	Carmen Peiro
FundHospUnivLaFe	Spain	Francisco Castera Brugada
GeorgeHuntingtonInst	Germany	Herwig Lange
GeorgeHuntingtonInst	Germany	Ralf Reilmann
GeorgeHuntingtonInst	Germany	Stefan Bohlen
GeorgeHuntingtonInst	Germany	Anja Kletsch
GeorgetownUniv	USA	Karen Anderson
GeorgetownUniv	USA	Natasha Scott
GreatGlasgowHealthBoard	UK	Murray Sutherland
GreatGlasgowHealthBoard	UK	Stuart Ritchie
GuyandStThomFounTrust	UK	Deborah Ruddy
GuyandStThomFounTrust	UK	Dene Robertson
GuyandStThomFounTrust	UK	Elizabeth White
GuyandStThomFounTrust	UK	Thomasin Andrews
Hennepin County Medical Center	USA	Dawn Radtke
Hennepin County Medical Center	USA	Martha Nance
HNDC	USA	Gregory Suter
HNDC	USA	William M Mallonee
HospCreuSantPau	Spain	Andrea Horta
HospCreuSantPau	Spain	Jaime Kulisevsky
HospInfantChrisBadaj	Spain	Carmen Durán Herrera
HospInfantChrisBadaj	Spain	Patrocinio García Moreno
HospMareMerce	Spain	Elvira Roca Goma
HospMareMerce	Spain	Jesús Miguel Ruíz Idiago
HospMarseille	France	Jean-Philippe Azulay
HospMarseille	France	Laura Mundler
HospUnivBellvitge	Spain	Matilde Calopa
HospUnivBellvitge	Spain	Jordi Bas
IndianaUniv	USA	Chris James
IndianaUniv	USA	Andrea Hurt
InstCervMoelle	France	Alexandra Durr
InstCervMoelle	France	Marie Biet
InstNeuroBuenoAires	Argentina	Martin Cesarini
InstNeuroBuenoAires	Argentina	Emilia Gatto
InstPathGenCharleroi	Belgium	Christine Verellen-Dumoulin
InstPathGenCharleroi	Belgium	Cécile Minet
InstPsychandNeuro	Poland	Grzegorz Witkowski

Site	Country	Name
InstPsychandNeuro	Poland	Iwona Stepniak
JimenDiazFoun	Spain	Pedro J Garcia Ruiz
JimenDiazFoun	Spain	Asunción Martinez
JohnsHopkinsUniv	USA	Frederick C. Nucifora Jr.
JohnsHopkinsUniv	USA	Christopher Ross
JohnsHopkinsUniv	USA	Mollie Jenckes
KboIsarAmpKlinTauf	Germany	Matthias Dose
KboIsarAmpKlinTauf	Germany	Michael Bachmaier
KboIsarAmpKlinTauf	Germany	Ralf Marquard
KrakowskaAkademiaNeuro	Poland	Monica Rudzinska
KrakowskaAkademiaNeuro	Poland	Marta Golosz
LeedsTeachHospTrust	UK	Alison Kraus
LeedsTeachHospTrust	UK	Stuart Jamieson
LeedsTeachHospTrust	UK	Ivana Markova
LeedsTeachHospTrust	UK	Emma Hobson
LeedsTeachHospTrust	UK	Callum Schofield
LegaItalRiceHunt	Italy	Simone Migliore
LegaItalRiceHunt	Italy	Sabrina Maffi
LegaItalRiceHunt	Italy	Maria Giovanna Dema
LegaItalRiceHunt	Italy	Barbara D'Alessio
LegaItalRiceHunt	Italy	Ferdinando Squitieri
Leicestershire	UK	Dawn Freire-Patino
Leicestershire	UK	Caroline Hallam
Leicestershire	UK	Reza Kiani
LeidenUniv	Netherlands	Raymund Roos
LeidenUniv	Netherlands	Marye Hogenboom
LilleUnivHosp	France	Clémence Simonin
LilleUnivHosp	France	Eric Decorte
Lisbon-Central	Portugal	Ana Calado
Lisbon-Instituto de Medicina Molecular	Portugal	Joaquim J Ferreira
LomaLindaUniv	USA	Dharmaseeli Moses
LothianHealthBoard	UK	Philip Greene
LothianHealthBoard	UK	Marie McGill
LothianHealthBoard	UK	Mary Porteous
MilanGenetic	Italy	Anna Castaldo
MilanGenetic	Italy	Caterina Mariotti
MilanGenetic	Italy	Lorenzo Nanetti
MilanNeuro	Italy	Dominga Paridi
MilanNeuro	Italy	Paola Soliveri
MilanNeuro	Italy	Simona Castagliuolo
MonashUniv	Australia	Nellie Georgious-Karistianis
MonashUniv	Australia	Lisa Mottram
MonashUniv	Australia	Katie Fitzgerald

Site	Country	Name
MonashUniv	Australia	Julie Stout
NHSForthValley	UK	David Thomson
NorStaffCombHeaTrust	UK	George El-Nimr
NorStaffCombHeaTrust	UK	Karen Kennedy
NorthBristolTrust	UK	Catherine Pennington
NorthBristolTrust	UK	Serena Dillon
NorthBristolTrust	UK	Elizabeth Coulthard
NorthBristolTrust	UK	Louise Gethin
NorthMetroHlthServ	Australia	Jacenta Abbott
NorthMetroHlthServ	Australia	Karen Clunies-Ross
NorthMetroHlthServ	Australia	Peter Panegyres
NorthumbTyneFreeman	UK	Jill Davison
NorthumbTyneFreeman	UK	Suresh Komati
NorthumbTyneFreeman	UK	Sarah Edwards
NorthYorkGen	Canada	Wai Lun Alan Fung
NorthYorkGen	Canada	Clare Gibbons
OhioStateUniv	USA	Allison Daley
OhioStateUniv	USA	Sandra Kostyk
OhioStateUniv	USA	Katherine Ambrogi
OhioStateUniv	USA	Areej Tariq
OxfordUnivHospTrust	UK	Andrea H Nemeth
OxfordUnivHospTrust	UK	Sarsha Wilson
ParaElenaKlinikKassel	Germany	Katrin Bürk
ParaElenaKlinikKassel	Germany	Claudia Trenkwalder
ParaElenaKlinikKassel	Germany	Grit Langhans
PlyHospNHSTrust	UK	Julie Frost
PlyHospNHSTrust	UK	Rupert Noad
PlyHospNHSTrust	UK	Jemma Inches
PooleHospFounTrust	UK	Annemieke Fox
PooleHospFounTrust	UK	John Burn
PoznanUniv	Poland	Daniel Zielonka
PoznanUniv	Poland	Elżbieta Alicja Puch
RamonCajalUnivHosp	Spain	José Luis López-Sendón Moreno
RamonCajalUnivHosp	Spain	Verónica Mañanes Barral
RockyMtnMovDis	USA	Liza Heap
RockyMtnMovDis	USA	Rajeev Kumar
RoyalDevExetFounTrst	UK	Sarah Irvine
RoyalDevExetFounTrst	UK	Timothy Harrower
RoyBerkNHSFounTrust	UK	Anita Foster
RoyBerkNHSFounTrust	UK	Richard Armstrong
RushUniv	USA	Alice Negron
RushUniv	USA	Jennifer Goldman
RutgersUniv	USA	Daniel Schneider
RutgersUniv	USA	Sheila Redding

Site	Country	Name
SanfordResearch	USA	Tish Skarloken
SanfordResearch	USA	Tanya Harlow
SanfordResearch	USA	Destini Spaeth
SchleswigHolsteinHosp	Germany	Alexander Münchau
SchleswigHolsteinHosp	Germany	Vera Tadic
SchleswigHolsteinHosp	Germany	Klaus Gehring
SheffieldChildFouTru	UK	Anya Kholkina
SheffieldChildFouTru	UK	Oliver Quarrell
SilesianMedUnivKatowice	Poland	Klaudia Plinta
SiloahAG	Switzerland	Jean-Marc Burgunder
SiloahAG	Switzerland	Jessica Koehli
SonEspasesHosp	Spain	Penélope Navas Arques
SonEspasesHosp	Spain	Ines Legarda
SouthamptonUnivHospTrust	UK	Christopher Kipps
SouthamptonUnivHospTrust	UK	Veena Agarwal
StAndrewsHealth	UK	Elvina Chu
StGeorgeHealthTrust	UK	Nayana Lahiri
StGeorgeHealthTrust	UK	Uruj Anjum
StJosefAndElisabethHosp	Germany	Carsten Saft
StJosefAndElisabethHosp	Germany	Rainer Hoffmann
StJosefAndElisabethHosp	Germany	Sarah von Hein
StJosefAndElisabethHosp	Germany	Barbara Daniela Kaminski
StJosefAndElisabethHosp	Germany	Jannis Achenbach
STJosephHosp	USA	Ryan Heeney
STJosephHosp	USA	Srivadee Oravivattanakul
StrasbourgUnivHosp	France	Christine Tranchant
StrasbourgUnivHosp	France	Fanny Huselstein
Tayside	UK	Alison Tonner
Tayside	UK	Lindsay Wilson
Tayside	UK	David Goudie
Tayside	UK	Paula McFadyen
TechUnivMunich	Germany	Adolf Weindl
TechUnivMunich	Germany	Antje Lüsebrink
ToulouseUnivHosp	France	Fabienne Calvas
ToulouseUnivHosp	France	Brigitte Pouzet
UnivAberdeen	UK	Daniela Rae
UnivAberdeen	UK	Alisdair Ross
UnivAberdeen	UK	Stella Sihlabela
UnivAberdeen	UK	Zosia Miedzybrodzka
UnivAlaBirm	USA	Victor Sung
UnivAlaBirm	USA	Jenna Smith
UnivAlberta	Canada	Oksana Suchowersky
UnivAlberta	Canada	Paul McCann

Site	Country	Name
UnivAlbertaGlenrose	Canada	Pam King
UnivAlbertaGlenrose	Canada	Wayne Martin
UnivBari	Italy	Marina de Tommaso
UnivBari	Italy	Marianna Delussi
UnivBologna	Italy	Cesa Scaglione
UnivBologna	Italy	Pietro Cortelli
UnivBritishCol	Canada	Tuan Le
UnivBritishCol	Canada	Lynn Raymond
UnivBritishCol	Canada	Blair Leavitt
UnivCalDavis	USA	Alexandra (Sasha) Duffy
UnivCalDavis	USA	Amanda Martin
UnivCalDavis	USA	Ashok Joshua Dayananthan
UnivCalDavis	USA	Vicki Wheelock
UnivCalgary	Canada	Lorelei Tainsh (Derwent)
UnivCalgary	Canada	Justyna Sarna
UnivCalIrvine	USA	Nicolas Phielipp
UnivCalIrvine	USA	Breana Chew
UnivCalLosAngeles	USA	Susan Perlman
UnivCalLosAngeles	USA	Jeffrey Carpio
UnivCalSanDiego	USA	Jody Corey-Bloom
UnivCalSanDiego	USA	Chase Snell
UnivCalSanFran	USA	Michael Geschwind
UnivCalSanFran	USA	Nancy Cai
UnivCalSanFran	USA	Alexandra Nelson
UnivCambridge	UK	Sarah Mason
UnivCambridge	UK	Caroline Williams-Gray
UnivCambridge	UK	Laura Sherlock
UnivCambridge	UK	Roger Barker
UnivCattolicaSacrCur	Italy	Flavia Torlizzi
UnivCattolicaSacrCur	Italy	Anna Rita Bentivoglio
UnivCattolicaSacrCur	Italy	Marcella Solito
UnivCharite	Germany	Josef Priller
UnivCharite	Germany	Stefanie Kehrer
UnivChicago	USA	Joan Young
UnivChicago	USA	Tao Xie
UnivCincinnatiPhysCo	USA	Andrew Duker
UnivCincinnatiPhysCo	USA	Jessica Doak
UnivCollLondon	UK	Ed Wild
UnivCollLondon	UK	Monica Lewis
UnivCollLondon	UK	Kate Fayer
UnivCollLondon	UK	Sarah Tabrizi
UnivConnHealthCtr	USA	Robin Zingales-Brown
UnivConnHealthCtr	USA	Glenn Konopaske

Site	Country	Name
UniverMedCtrFreiburg	Germany	Gerit Kammel
UniverMedCtrFreiburg	Germany	Stephan Klebe
UniverMedCtrFreiburg	Germany	Michel Rijntjes
UnivFlorida	USA	Nikolaus McFarland
UnivFlorida	USA	Erin Monari
UnivGenova	Italy	Paola Mandich
UnivGenova	Italy	Roberta Marchese
UnivGroningen	Netherlands	H.P.H. Kremer
UnivGroningen	Netherlands	Maxime Vos
UnivHosGustavDresden	Germany	Andreas Hermann
UnivHosGustavDresden	Germany	Simone Schmidt
UnivHospAachen	Germany	Beate Schumann
UnivHospAachen	Germany	Kathrin Reetz
UnivHospCopenhagen	Denmark	Christina Vangsted Hansen
UnivHospCopenhagen	Denmark	Jørgen Nielsen
UnivHospCopenhagen	Denmark	Lena E. Hjermind
UnivHospCopenhagen	Denmark	Suzanne Granhøj Lindquist
UnivHospCopenhagen	Denmark	Marie Nathalie Nickelsen Hellem
UnivHospErlangen	Germany	Susanne Seifert
UnivHospErlangen	Germany	Zacharias Kohl
UnivHospErlangen	Germany	Jürgen Winkler
UnivHospGiessenMarburg	Germany	Katrin Bürk
UnivHospLeuven	Belgium	Wim Vandenberghe
UnivHospLeuven	Belgium	Petra Weckx
UnivHospOdense	Denmark	Lene Wermuth
UnivHospOdense	Denmark	Marianne Dybro Lundsgaard
UnivHospUlm	Germany	Hela Jerbi
UnivHospUlm	Germany	Jan Lewerenz
UnivHospUlm	Germany	Michael Orth
UnivHospUlm	Germany	Sonja Trautmann
UnivHospUlm	Germany	Katrin Lindenberg
UnivHospWuerzburg	Germany	Kerstin Nöth
UnivHospWuerzburg	Germany	Christine Leypold
UnivHospWuerzburg	Germany	Kai Boelmans
UnivIllinois	USA	Mitch King
UnivIllinois	USA	Sadie Foster
UnivInnsbruck	Austria	Klaus Seppi
UnivInnsbruck	Austria	Dora Valent
UnivIowa	USA	Amanda Heinzerling
UnivIowa	USA	Jane S Paulsen
UnivKansasMedCtrResInst	USA	Carolyn Gray
UnivKansasMedCtrResInst	USA	Richard Dubinsky
UnivLouisville	USA	Kathrin LaFaver

Site	Country	Name
UnivLouisville	USA	Annette Robinson
UnivMaryland	USA	Terra Hill
UnivMaryland	USA	William Keller
UnivMelbourne	Australia	Anita Goh
UnivMelbourne	Australia	Stephanie Perin
UnivMich	USA	Jenna Russell
UnivNaples	Italy	Luigi di Maio
UnivNaples	Italy	Alessandro Roca
UnivNaples	Italy	Silvio Peluso
UnivNaples	Italy	Elena Salvatore
UnivNaples	Italy	Giuseppe De Michele
UnivNebMedCtr	USA	Nick Miller
UnivNebMedCtr	USA	Amy Hellman, MD
UnivOtago	New Zealand	Laura Paermentier
UnivOtago	New Zealand	Tim Anderson
UnivPenn	USA	Pedro Gonzalez-Alegre
UnivPenn	USA	Jennifer Klapper
UnivPitt	USA	Patricia Conlon
UnivPitt	USA	Valerie Suski
UnivRochester	USA	Amy Chesire
UnivRochester	USA	Frederick Marshall
UnivRochester	USA	Justin Alves
UnivSoCar	USA	Miroslav Cuturic
UnivSoCar	USA	Alyson Grant
UnivSouthFlorida	USA	Danielle Hergert
UnivSouthFlorida	USA	Patricia Johnson
UnivSouthFlorida	USA	Emily Kellogg
UnivSouthFlorida	USA	Juan Sanchez-Ramos
UnivSouthFlorida	USA	Kelly (Kollen) Elliott
UnivTenn	USA	Mark LeDoux
UnivTenn	USA	Amanda Nolte
UnivTexasHlthCntrHous	USA	Erin Furr Stimming
UnivTexasHlthCntrHous	USA	Jamie Sims
UnivUtah	USA	Meghan Zorn
UnivUtah	USA	Matthew Halverson
UnivUtah	USA	Stefan Pulst
UnivUtah	USA	Paola Wall
UnivVermont	USA	Roman Pettigrew
UnivVermont	USA	James Boyd
UnivVirginia	USA	Madeline B. Harrison
UnivVirginia	USA	Dana Morrissey
UnivWarsaw	Poland	Piotr Janik
UnivWarsaw	Poland	Natalia Szejko

Site	Country	Name
UnivWash	USA	Ali Samii
UnivWash	USA	Debra Del Castillo
VanderbiltUniv	USA	Elizabeth Huitz
VanderbiltUniv	USA	Daniel O. Claassen
VirgenCaminoHosp	Spain	Maite Lizarraga Rojas
VirgenCaminoHosp	Spain	María Antonia Ramos Arroyo
VirginiaCommUniv	USA	Claudia Testa
VirginiaCommUniv	USA	Ginger Norris
WakeForestUniv	USA	Christine O'Neill
WakeForestUniv	USA	Francis Walker
WaltonCtrFounTrust	UK	Louise Pate
WaltonCtrFounTrust	UK	Rhys Davies
WashingtonUniv	USA	Joel S. Perlmutter
WashingtonUniv	USA	Stacey Barton
WashingtonUniv	USA	Elaine Most
WestSydneyHlthDist	Australia	Clement Loy
WestSydneyHlthDist	Australia	Jillian McMillan
WestSydneyHlthDist	Australia	Therese Alting

Note: An Acknowledgement list with email addresses is available to qualified researchers
CLICK HERE to become a qualified researcher
