# Supplementary material for: DNA methylation study of Huntington’s disease and motor progression in patients and in animal models
Source: Nat Commun. 2020 Sep 10;11:4529. doi: 10.1038/s41467-020-18255-5 (PMC7484780; doi:10.1038/s41467-020-18255-5)
Supplement: Supplementary file 8 — Reporting Summary [file 41467_2020_18255_MOESM8_ESM.pdf]

natureresearch

Corresponding author(s): Steve Horvath

Last updated by author(s): 03/09/2010

Reporting Summary

Nature Research wishes to improve the reproducibility of the work that we publish. This form provides structure for consistency and transparency in reporting. For further information on Nature Research policies, seeAuthors & Referees and theEditorial Policy Checklist .

Statistics

For all statistical analyses, confirm that the following items are present in the figure legend, table legend, main text, or Methods section.

n/a	Confirmed	
☐	☒	The exact sample size (*n*) for each experimental group/condition, given as a discrete number and unit of measurement
☐	☒	A statement on whether measurements were taken from distinct samples or whether the same sample was measured repeatedly
☐	☒	The statistical test(s) used AND whether they are one- or two-sided<br>*Only common tests should be described solely by name; describe more complex techniques in the Methods section.*
☐	☒	A description of all covariates tested
☐	☒	A description of any assumptions or corrections, such as tests of normality and adjustment for multiple comparisons
☐	☒	A full description of the statistical parameters including central tendency (e.g. means) or other basic estimates (e.g. regression coefficient) AND variation (e.g. standard deviation) or associated estimates of uncertainty (e.g. confidence intervals)
☐	☒	For null hypothesis testing, the test statistic (e.g. *F*, *t*, *r*) with confidence intervals, effect sizes, degrees of freedom and *P* value noted<br>*Give P values as exact values whenever suitable.*
☒	☐	For Bayesian analysis, information on the choice of priors and Markov chain Monte Carlo settings
☐	☒	For hierarchical and complex designs, identification of the appropriate level for tests and full reporting of outcomes
☐	☒	Estimates of effect sizes (e.g. Cohen's *d*, Pearson's *r*), indicating how they were calculated

Our web collection on statistics for biologists contains articles on many of the points above.

Software and code

Policy information about availability of computer code

Data collection

MTA agreements with the epidemiological cohort (Enroll HD) prohibit us from sharing the protected health information of these human participants with anybody. Rather, the owner of the data (Enroll HD) has to be contacted. These are highly sensitive data involving rare genetic mutations and clinical information in humans. Fortunately, the cohort Enroll-HD will make these data available to all qualified users as detailed. Details: the human data were collected by two cohort studies: Enroll-HD and Registry HD. The data contain sensitive genetic information regarding mutation status and sensitive clinical information. No software was used for data collection. While all of this information will be made available to everybody, users have to request access to these data from the ENROLL HD cohort. We will insert the following paragraph on data availability. The data used in this publication will be made available upon request. Please direct inquiries to info@chdifoundation.org with the words "Enroll-HD Methylation data" in the subject line."

Data analysis

The software code underlying the epigenetic clock measurements can be found in the Supplements of the respective scientific papers. In addition, we provide a userfriendly online tool, https://dnamage.genetics.ucla.edu/home. The entire analysis was conducted in R 3.6.2 software . R packages and R functions (anRichment (3.6), coMET (3.1), minfi/gaphunter (3.6),nlme(3.1-148),WGCNA (3.6.3)) have been specified in methods and the corresponding versions are dependent to our R current version (3.6.2). The other software packages used in our data analysis was Metal (version released on 2011-03-25). Souce of data is clearly stated.

For manuscripts utilizing custom algorithms or software that are central to the research but not yet described in published literature, software must be made available to editors/reviewers. We strongly encourage code deposition in a community repository (e.g. GitHub). See the Nature Research guidelines for submitting code & software for further information.

Data

Policy information about availability of data

All manuscripts must include a data availability statement. This statement should provide the following information, where applicable:

- Accession codes, unique identifiers, or web links for publicly available datasets
- A list of figures that have associated raw data
- A description of any restrictions on data availability

Please see the following section.
Data Availability
Our data are available from two data repositories. First, Gene Expression Omnibus (Superseries GSE147004 and subseries GSE146917, GSE147002, GSE147003, GSE72778 (human brain methylation). Second, from Enroll HD https://www.enroll-hd.org/. Please direct inquiries to info@chdifoundation.org with the words "Enroll-HD Methylation data" in the subject line.

Field-specific reporting

Please select the one below that is the best fit for your research. If you are not sure, read the appropriate sections before making your selection.

☒ Life sciences ☐ Behavioural & social sciences ☐ Ecological, evolutionary & environmental sciences

For a reference copy of the document with all sections, see nature.com/documents/nr-reporting-summary-flat.pdf

Life sciences study design

All studies must disclose on these points even when the disclosure is negative.

Sample size	All sample sizes are reported in Table 1, Supplementary Tables 1-3 and/or Methods section. Please see the details added in the end of page 5
Data exclusions	Data were not excluded. We report all data that were evaluated irrrespective of the outcome.
Replication	All attempts at replication were successful. In Enroll-HD, genotyping of CAG expansion was performed in a research lab. Andependent Data Safety Monitoring Committee (DSMC). More details are listed in Supplementary Note 2: Study Population from Enroll-HD./ Research Genotyping.
Randomization	Not applicable. Our study is an observational study that combines results from large epidemiological cohorts.
Blinding	Not applicable. Our study is an observational study that combines results from large epidemiological cohorts.

Replication for mouse data:All mouse studies were performed with N=8 mice (4 males and 4 females) per tissue per genotype, so they are biological replicates. DNA methylation data were profiled in with two different methods of array (RRBS and customized methylation array) using different cohorts of mice. Replication results were observed. Replicaiton for sheep: A total of 153 cases were analyzed, 39 cases were run in duplicate.

Reporting for specific materials, systems and methods

We require information from authors about some types of materials, experimental systems and methods used in many studies. Here, indicate whether each material, system or method listed is relevant to your study. If you are not sure if a list item applies to your research, read the appropriate section before selecting a response.

Materials & experimental systems

n/a	Involved in the study	
☒	☐	Antibodies
☒	☐	Eukaryotic cell lines
☒	☐	Palaeontology
☐	☒	Animals and other organisms
☐	☒	Human research participants
☐	☒	Clinical data

Methods

n/a	Involved in the study	
☒	☐	ChIP-seq
☒	☐	Flow cytometry
☒	☐	MRI-based neuroimaging

Antibodies

Antibodies used	Not applicable
Validation	Not applicable

Eukaryotic cell lines

Policy information about cell lines

Cell line source(s)	Not applicable
Authentication	Not applicable
Mycoplasma contamination	Not applicable
Commonly misidentified lines (See ICLAC register)	Not applicable

Palaeontology

Specimen provenance	*Provide provenance information for specimens and describe permits that were obtained for the work (including the name of the issuing authority, the date of issue, and any identifying information).*
Specimen deposition	*Indicate where the specimens have been deposited to permit free access by other researchers.*
Dating methods	*If new dates are provided, describe how they were obtained (e.g. collection, storage, sample pretreatment and measurement), where they were obtained (i.e. lab name), the calibration program and the protocol for quality assurance OR state that no new dates are provided.*

☐ Tick this box to confirm that the raw and calibrated dates are available in the paper or in Supplementary Information.

Animals and other organisms

Policy information about studies involving animals; ARRIVE guidelines recommended for reporting animal research

Laboratory animals	Mouse: For mouse RRBS assay, 6m heterozygous Htt KI Q175 and Q20 mice at C57BL/6j background were used (with N=8 per genotype, as 4 males and 4 females). For mouse methylation array, 6m heterozygous Htt KI Q175 mice and their littermate wildtype control mice at C57BL/6j background were used (also with N=8 per gentoype, as 4 males and 4 females).<br>Sheep: We analyzed a total of 168 sheep (57% females): 84 HD transgenic sheep age matched with 84 sheep as controls. The age of sheep ranged from 2.9 to 7.0 years with mean±SD = 4.1±0.8. The species of sheep is Ovis aries Linneaus (Breed: South Australian Merino)
Wild animals	The study did not involve wild animals
Field-collected samples	The study did not involve samples collected from the field
Ethics oversight	Human data from Enroll HD and Registry:All sites are required to obtain and maintain local Ethics Committee approvals as detailed in Methods.<br>For mouse samples:The protocols were approved by the Institutional Animal Care and Use Committee of PsychoGenics, Inc., an AAALAC International accredited institution (Unit #001213).Mouse methylation array study was approved by UCLA Institutional Animal Care and Use Committees.<br>For sheep samples:All protocols used were approved by the University of Auckland Animal Ethics Committee (New Zealand) and the SARDI/PIRSA Animal Ethics Committee (Approval number 19/02). All work involving OVT73 Sheep was approved by Primary Industries and Regions South Australia (PIRSA) Animal Ethics Committee with oversite from the University of Auckland Animal Ethics Committee.

Note that full information on the approval of the study protocol must also be provided in the manuscript.

Human research participants

Policy information about studies involving human research participants

Population characteristics	The study involves participants in Enroll-HD cohort and Registry-HD cohort. The Enroll-HD cohort involves HD mutation carriers and controls. The mean ages at baseline was 51 (years) in the control group and 49 in the HD mutation group, respectively. The Registry cohort involves manifest HD patients and controls. At baseline, the mean ages were 49 years in the control group and 53 years in the HD group, respectively. Both gender participants were recruited in both the study cohorts. The detailed characters of study participants are listed in Table 1, Supplementary Tables 1-3 and Methods.
Recruitment	Patients with HD and their family members are recruited from specialty clinics (Human Genetics, Neurology, Psychiatry) that advise and treat people affected by HD. In addition, in some areas community clinics and neurologists who see HD patients will recruit participants for this study. More details for the study population including inclusion and exclusion criteria are listed in Supplementary Note 2, Participants may also receive information about the study through a website, clinical practices, support groups, advocacy newsletters, etc. and place a direct request to be considered for participation in the study. Community conrols are identified, using advertisements, flyers and newsletters, by study site staff with the support of the Enroll-HD operational staff.<br>Inclusion Criteria:<br>Carriers: This group comprises the primary study population and consists of individuals who carry the HD gene expansion mutation.<br>Controls: This group comprises the comparator study population and consists of individuals who do not carry the HD expansion mutation.

These two major categories can be further subdivided into six different subgroups of eligible individuals:
• Manifest/Motor-manifest HD: Carriers with clinical features that are regarded in the opinion of the investigator as diagnostic of HD.
• Pre-Manifest/-Motor-manifest HD: Carriers without clinical features regarded as diagnostic of HD.
Genotype Unknown: This group includes a first or second degree relative, i.e., related by blood to a carrier, who has not undergone predictive testing for HD and therefore has an undetermined carrier status.
Genotype Negative: This group includes a first or second degree relative, i.e., related by blood to a carrier, who has undergone predictive testing for HD and is known not to carry the HD expansion mutation.
Family Control: Family members or individuals not related by blood to carriers (e.g., spouses, partners, caregivers).
Community Controls: Individuals unrelated to HD carriers who did not grow up in a family affected by HD. Data collected from community controls are used for generation of normative data for sub-studies.
Participant status is captured in the study database using 2 variables: 1) Investigator Determined Status: this will be based on clinical signs and symptoms and genotyping performed as part of medical care, and is updated at every visit and 2) Research Genotyping Status: this is based on genotyping conducted as part of Enroll-HD study procedures. Based on research genotyping, participants are reclassified under this variable from Genotype Unknown to 'Carriers' or 'Controls'. Investigators and participants are blinded to this reclassification.
Exclusion Criteria: Individuals with chorea movement disorders in the context of a negative test for the HD gene mutation. For Community Controls: those individuals with a major central nervous system disorder are excluded (e.g. stroke, Parkinson disease, Multiple Sclerosis, etc.).

Ethics oversight

Enroll-HD: The ethics oversight is listed in Enroll-HD protocol, https://www.enroll-hd.org/enrollhd_documents/Enroll-HD-Protocol-1.0.pdf. The IRB is approved by the Scientific Review Committee (SRC). Ethical review for substudy protocols listed below will be performed concurrently with the main Enroll-HD study protocol; future sub-study protocols will be submitted as minor protocol amendments. Informed consent for participation in sub-studies will be obtained within the informed consent forms for the main Enroll-HD study.
Proposed sub-studies include:

a. Pre-motor manifest HD - to further develop and validate outcome measures to detect and track alterations in prodromal stages of HD
b. Advanced stage HD-to validate the “Advanced-stage UHDRS” for use in clinical and research practices
c. Juvenile-onset HD-to develop and validate new scales and/or modify existing scales for outcomes of interest in this patient population
d. Frontal behaviors-to validate existing measures of frontal behaviors (e.g. Apathy scales, FrSBe) in HD populations and develop sub-scales as outcome measures for trials
e. Linguistic abilities- to characterize language impairment and assess syntactic abilities using the Sentence-Picture Matching Task
f. General cognitive impairment- to validate the Montreal Cognitive Assessment for use in HD
g. Tapping as outcome measure
h. HD Quality of Life outcome measure-to develop and validate a patient-centered prototype health-related quality of life questionnaire specific for HD
i. Physiotherapy outcome measures- to develop physiotherapy related outcome measures for use in future interventional studies
j. Lifestyle factors- to examine the link between lifestyle factors.
#=================
The European HD Network (EHDN) provided us with whole DNA samples from the REGISTRY core research project. The information regarding the ethnic insight can be found in NIH clinical trial database,https://clinicaltrials.gov/ct2/show/record/NCT01590589.
REGISTRY integrates prospectively and systematically collected clinical research data (e.g. phenotypical clinical features, family history, demographical characteristics) with access to biological specimens (e.g. blood, urine) obtained from individuals with manifest HD, unaffected individuals known to carry the HD mutation or at risk of carrying the HD mutation, and control research participants (e.g. spouses, siblings or offspring of HD mutation carriers known not to carry the HD mutation).
REGISTRY is an open-ended study and eligible subjects are assessed at annual study visits on the phenotypical characteristics of HD regardless of whether they display clinical symptoms and signs of the disease and of individuals who are part of an HD family (irrespective of their mutation carrier status). At each study visit, general clinical, motor function, behavior, cognitive, Health Economics, Quality of Life assessments are administered. In addition, participants are given the option to consent to the donation of biosamples for the purposes of mutation (CAG repeat length) testing and for research to identify biological modifiers and markers of HD. Biological specimens and phenotypical data are made available to qualified scientists whose projects are reviewed and approved by the Scientific and Bioethical Advisory Committee (SBAC) of EHDN. Successful applicants agree to accept the EHDN policies surrounding the use of the data/materials provided and publication of results (see data sharing and publication policies of EHDN, attached). Research projects should aim to advance scientific knowledge towards establishing

clinically effective treatments that delay onset and/or slow the progression of the disease.

Note that full information on the approval of the study protocol must also be provided in the manuscript.

Clinical data

Policy information about clinical studies

All manuscripts should comply with the ICMJEguidelines for publication of clinical research and a completedCONSORT checklist must be included with all submissions.

Clinical trial registration

Not applicable

Study protocol

The study protocol of EnrollHD cohort can be found in https://www.enroll-hd.org/enrollhd_documents/Enroll-HD-Protocol-1.0.pdf. The study protocol of REGISTRY cohort is listed in NIH ClinicalTrial database, https://clinicaltrials.gov/ct2/show/record/NCT01590589.

Data collection

Outcomes

In Methods:
Below we described our study population from Enroll-HD. Patients with HD and their family members are recruited from specialty clinics (Human Genetics, Neurology, Psychiatry) that advise and treat people affected by HD. In addition, in some areas community clinics and neurologists who see HD patients will recruit participants for this study. More details for the study population including inclusion and exclusion criteria are listed in Supplementary Note 2.

For outcomes:
Supplementary Note 1: The Unified Huntington's Disease Rating Scale
The Unified Huntington's Disease Rating Scale (UHDRS) was developed as a clinical rating scale to assess four domains of clinical performance and capacity in HD: motor function, cognitive function, behavioral abnormalities, and functional capacity, developed by Huntington Study Group (HSG) 1. The HSG has extensively assessed the internal consistency and the intercorrelations of the four domains and examined changes in ratings over time. The UHDRS is used to quantify the severity of disease in Huntington patients for clinical assessment. The UHDRS total motor score is a sum of 31 items across oculomotor function, dysarthria, chorea, dystonia, gait, and postural stability. Each item is a scale from 0 to 4, indicating no abnormalities to the most severe impairment. The possible range of the total motor score is zero to124.

Sample Size:
Enroll-HD is a cooperative effort to build a large linked dataset of clinical data and biosamples. While each project proposal defining a specific outcome or endpoint will include a sample size calculation and/or power analysis specific to the objectives of that particular study, in general, the sample size and open ended enrollment is planned a) to facilitate genetic modifier studies that require large numbers to reliably identify genes of interest and their modifiers, b) to identify distinct phenotypes that are infrequent and therefore require large numbers for detection, c) to explore a diversity of environmental modifiers and gene-environment interactions, and d) to build disease models to study prognostic factors and rates of progression. Since all questions will not necessarily require the entire sample of participants, only a subset of assessments are delineated as core assessments and required on all participants at all sites. In our study, most of the analysis involved the subjects with DNA methylation array profiles as an extended assessment. In general, extended assessments are to be collected on most participants as often as possible, but due to the planned large number of participants, it is anticipated that even with missing data, there will be sufficient number of participants with extended assessments, and therefore, sufficient power for proposed analyses.
